# Supplementary material for: Bacterial aetiology, antimicrobial susceptibility patterns, and factors associated with urinary tract infection among under-five children at primary health facility, North-Western Tanzania
Source: PLoS One. 2024 May 6;19(5):e0303369. doi: 10.1371/journal.pone.0303369 (PMC11073726; doi:10.1371/journal.pone.0303369)
Supplement: Attachment — Submitted filename: UTI-commented.pdf [file pone.0303369.s002.pdf]

PLOS ONE

Etiologic Profile, Antimicrobial Susceptibility Patterns, and Factors Associated with Urinary Tract Infection among Under-five Children at Primary Health Facility, North-Western Tanzania

--Manuscript Draft--

**Manuscript Number:**	PONE-D-23-32229
**Article Type:**	Research Article
**Full Title:**	Etiologic Profile, Antimicrobial Susceptibility Patterns, and Factors Associated with Urinary Tract Infection among Under-five Children at Primary Health Facility, North-Western Tanzania
**Short Title:**	Etiologic Profile, Antimicrobial Susceptibility Patterns, and Factors Associated with Urinary Tract Infection
**Corresponding Author:**	Roza Ernest<br>MUHAS: Muhimbili University of Health and Allied Sciences<br>Dar es Salaam, TANZANIA, UNITED REPUBLIC OF
**Keywords:**	Urinary Tract Infection; Antimicrobial susceptibility pattern; under-fives
**Abstract:**	Abstracts<br><br>Background<br><br>Urinary tract infection (UTI) is the second most common infection in the community after acute respiratory tract infections. The incidence of UTI varies with age, gender, health condition, and geographic location. The prevalence of bacteriuria in children under the age of five in rural settings is a topic that has received limited attention and research. This study determined the proportion of UTI, aetiology, antibiotic susceptibility profile, and factors associated with UTI among febrile under-five children.<br><br>Methodology<br><br>The cross-sectional analytical study was conducted at the District Hospital in Mwanza Region, North-Western Tanzania, between March and April 2023. A non-probability convenient sampling technique was used to recruit febrile under-five children. Participants' information was collected using a structured questionnaire. Urine was collected for urine culture and antimicrobial susceptibility testing. STATA version 15.0 was used for data analysis. Factors associated with UTI were determined by a modified Poisson model.<br><br>Result<br><br>The study recruited 368 under-five children; 194 (52.7%) were males. The study participants' median age (IQR) was 24 (13-36) months. Of all, 28.8% had laboratory-confirmed UTI. One hundred and six pathogens were isolated, the majority being Escherichia coli, 37 (34.9%), and Staphylococcus aureus, 26 (24.5%). Escherichia coli were highly susceptible to cefepime, piperacillin-tazobactam, nitrofurantoin, and meropenem by 81.1%, 86.5%, 86.5%, and 97.3%, respectively. Multidrug resistance was observed in 33.0% of isolates, Methicillin-Resistance Staphylococcus aureus in 23.1% of Staphylococcus aureus, and Extended Spectrum beta-lactamases in 25% of gram-negative bacteria. UTI infection was associated with vomiting, age, dysuria, abdominal pain, nappy use, and uncircumcision.<br><br>Conclusion<br><br>Our study found a relatively high proportion of UTI among under-five children. Escherichia coli and Staphylococcus aureus were the commonly isolated organisms highly susceptible to nitrofurantoin, ciprofloxacin, meropenem, cefepime, and piperacillin-tazobactam, and least susceptible to commonly used antibiotics. Empirical

	treatment of UTI should be reviewed periodically based on the antibiotic susceptibility pattern.
**Order of Authors:**	Roza Ernest
	Nsiande Lema
	Sued Yassin
	Agricola Joachim
	Mtebe Majigo
**Additional Information:**	
**Question**	**Response**
**Financial Disclosure**<br><br>Enter a financial disclosure statement that describes the sources of funding for the work included in this submission. Review the submission guidelines for detailed requirements. View published research articles from *PLOS ONE* for specific examples.<br><br>This statement is required for submission and **will appear in the published article** if the submission is accepted. Please make sure it is accurate.<br><br>**Funded studies**<br>Enter a statement with the following details:<br>• Initials of the authors who received each award<br>• Grant numbers awarded to each author<br>• The full name of each funder<br>• URL of each funder website<br>• Did the sponsors or funders play any role in the study design, data collection and analysis, decision to publish, or preparation of the manuscript?<br><br>Did you receive funding for this work?	Yes
Please add funding details.<br>as follow-up to "**Financial Disclosure**<br><br>Enter a financial disclosure statement that describes the sources of funding for the work included in this submission. Review the submission guidelines for detailed requirements. View published research articles from *PLOS ONE* for specific	This work is supported by the United States President's Emergency Plan for AIDS Relief (PEPFAR) through the Centre of Excellence in Health Monitoring and Evaluation, Mzumbe University under the U.S Centers for Disease Control and Prevention (CDC), Project Cooperative Agreement No: NU2GGH002292 which supports Tanzania Field Epidemiology and Laboratory Training Program (TFELTP), however, the funder did not contribute to the study's design, data collection, analysis, interpretation, or manuscript writing

examples.<br><br>This statement is required for submission and **will appear in the published article** if the submission is accepted. Please make sure it is accurate.<br><br>**Funded studies**<br>Enter a statement with the following details:<br>• Initials of the authors who received each award<br>• Grant numbers awarded to each author<br>• The full name of each funder<br>• URL of each funder website<br>• Did the sponsors or funders play any role in the study design, data collection and analysis, decision to publish, or preparation of the manuscript?<br><br>Did you receive funding for this work?"	
Please select the country of your main research funder (please select carefully as in some cases this is used in fee calculation).<br>as follow-up to "**Financial Disclosure**<br><br>Enter a financial disclosure statement that describes the sources of funding for the work included in this submission. Review the submission guidelines for detailed requirements. View published research articles from *PLOS ONE* for specific examples.<br><br>This statement is required for submission and **will appear in the published article** if the submission is accepted. Please make sure it is accurate.	UNITED STATES - US

**Funded studies**<br>Enter a statement with the following details:<br>• Initials of the authors who received each award<br>• Grant numbers awarded to each author<br>• The full name of each funder<br>• URL of each funder website<br>• Did the sponsors or funders play any role in the study design, data collection and analysis, decision to publish, or preparation of the manuscript?<br><br>Did you receive funding for this work?"	
**Competing Interests**<br><br>Use the instructions below to enter a competing interest statement for this submission. On behalf of all authors, disclose any competing interests that could be perceived to bias this work—acknowledging all financial support and any other relevant financial or non-financial competing interests.<br><br>This statement is required for submission and **will appear in the published article** if the submission is accepted. Please make sure it is accurate and that any funding sources listed in your Funding Information later in the submission form are also declared in your Financial Disclosure statement.<br><br>View published research articles from *PLOS ONE* for specific examples.	The authors of this research have explicitly stated that there are no existing conflicts of interest related to the publication of their work.

**NO authors have competing interests**<br><br>Enter: *The authors have declared that no competing interests exist.*<br><br>**Authors with competing interests**<br><br>Enter competing interest details beginning with this statement:<br><br>*I have read the journal's policy and the authors of this manuscript have the following competing interests: [insert competing interests here]*<br><br>* typeset	
**Ethics Statement**<br><br>Enter an ethics statement for this submission. This statement is required if the study involved:<br><br>• Human participants<br>• Human specimens or tissue<br>• Vertebrate animals or cephalopods<br>• Vertebrate embryos or tissues<br>• Field research<br><br>Write "N/A" if the submission does not require an ethics statement.<br><br>General guidance is provided below. Consult the submission guidelines for detailed instructions. **Make sure that all information entered here is included in the Methods section of the manuscript.**	The study obtained ethical clearance (MUHAS-REC-12-2022-1484) from the Muhimbili University of Health and Allied Sciences Research and Ethics Committee. In addition, we got permission for data collection from the offices of the Regional and District Medical Officers. Parents or guardians were informed about the study objectives and consented on behalf of their children before inclusion in the study. Results of urine culture and antimicrobial susceptibility tests were timely communicated to the attending clinician for patient management. Participant identification numbers were used in the study instead of names to ensure confidentiality.

Powered by Editorial Manager® and ProduXion Manager® from Aries Systems Corporation

**Format for specific study types**<br><br>**Human Subject Research (involving human participants and/or tissue)**<br>• Give the name of the institutional review board or ethics committee that approved the study<br>• Include the approval number and/or a statement indicating approval of this research<br>• Indicate the form of consent obtained (written/oral) or the reason that consent was not obtained (e.g. the data were analyzed anonymously)<br><br>**Animal Research (involving vertebrate animals, embryos or tissues)**<br>• Provide the name of the Institutional Animal Care and Use Committee (IACUC) or other relevant ethics board that reviewed the study protocol, and indicate whether they approved this research or granted a formal waiver of ethical approval<br>• Include an approval number if one was obtained<br>• If the study involved *non-human primates*, add *additional details* about animal welfare and steps taken to ameliorate suffering<br>• If anesthesia, euthanasia, or any kind of animal sacrifice is part of the study, include briefly which substances and/or methods were applied<br><br>**Field Research**<br><br>Include the following details if this study involves the collection of plant, animal, or other materials from a natural setting:<br>• Field permit number<br>• Name of the institution or relevant body that granted permission	
**Data Availability**<br><br>Authors are required to make all data underlying the findings described fully available, without restriction, and from the time of publication. PLOS allows rare exceptions to address legal and ethical concerns. See the PLOS Data Policy and FAQ for detailed information.	No - some restrictions will apply

A Data Availability Statement describing where the data can be found is required at submission. Your answers to this question constitute the Data Availability Statement and **will be published in the article**, if accepted.<br><br>**Important:** Stating 'data available on request from the author' is not sufficient. If your data are only available upon request, select 'No' for the first question and explain your exceptional situation in the text box.<br><br>Do the authors confirm that all data underlying the findings described in their manuscript are fully available without restriction?	
**Describe where the data may be found in full sentences. If you are copying our sample text, replace any instances of XXX with the appropriate details.**<br><br>• If the data are **held or will be held in a public repository**, include URLs, accession numbers or DOIs. If this information will only be available after acceptance, indicate this by ticking the box below. For example: *All XXX files are available from the XXX database (accession number(s) XXX, XXX.).*<br>• If the data are all contained **within the manuscript and/or Supporting Information files**, enter the following: *All relevant data are within the manuscript and its Supporting Information files.*<br>• If neither of these applies but you are able to provide **details of access elsewhere**, with or without limitations, please do so. For example:<br><br>*Data cannot be shared publicly because of [XXX]. Data are available from the XXX Institutional Data Access / Ethics Committee (contact via XXX) for researchers who meet the criteria for access to confidential data.*<br><br>*The data underlying the results presented in the study are available from (include the name of the third party*	The datasets used in this study are publicly available and can be acquired upon request from the corresponding author.

and contact information or URL).<br>• This text is appropriate if the data are owned by a third party and authors do not have permission to share the data.<br><br>* typeset	
Additional data availability information:	

Powered by Editorial Manager® and ProduXion Manager® from Aries Systems Corporation

Manuscript
Click here to access/download;Manuscript;Manuscript_UTI Underfive_ Roza et al_Final.docx

Etiologic Profile, Antimicrobial Susceptibility Patterns, and Factors Associated with Urinary Tract Infection among Under-five Children at Primary Health Facility, North-Western Tanzania

Roza Ernest[1,2*], Nsiande Lema[1], Sued Yassin[3], Agricola Joachim[4], Mtebe Majigo[4].

[1] Tanzania Field Epidemiology and Laboratory Training Program, Dar es Salaam, Tanzania
[2] Department of Epidemiology and Biostatistics, Muhimbili University of Health and Allied Sciences, Dar es Salaam, Tanzania
[3] Research, Training, and Consultancy Unit, Chato Zonal Referral Hospital, Geita, Tanzania
[4]Department of Microbiology and Immunology, Muhimbili University of Health and Allied Sciences, Dar es Salaam, Tanzania

***Email:** roza.ernest@yahoo.com (RE)

Abstracts

Background: Urinary tract infection (UTI) is the second most common infection in the community after acute respiratory tract infections. The incidence of UTI varies with age, gender, health condition, and geographic location. The prevalence of bacteriuria in children under the age of five in rural settings is a topic that has received limited attention and research. This study determined the proportion of UTI, aetiology, antibiotic susceptibility patterns, and factors associated with UTI among febrile under-five children.

Methodology: The cross-sectional analytical study was conducted at the District Hospital in Mwanza Region, North-Western Tanzania, between March and April 2023. A non-probability convenient sampling technique was used to recruit febrile under-five children. Participants' information was collected using a structured questionnaire. Urine was collected for urine culture and antimicrobial susceptibility testing. STATA version 15.0 was used for data analysis. Factors associated with UTI were determined using a modified Poisson model.

Result: The study recruited 368 under-five children; 194 (52.7%) were males. The median age (IQR) was 24 (13-36) months. Of all, 28.8% had laboratory-confirmed UTI. One hundred and six pathogens were isolated, the majority being *Escherichia coli*, 37 (34.9%), and

Staphylococcus aureus, 26 (24.5%). *Escherichia coli* were highly susceptible to cefepime, piperacillin-tazobactam, nitrofurantoin, and meropenem, ranging from 81.1% to 97.3%. Multidrug resistance was observed in 33.0% of isolates, Methicillin-Resistance *Staphylococcus aureus* in 23.1% of Staphylococcus *aureus*, and Extended Spectrum beta-lactamases in 25% of gram-negative bacteria. UTI infection was associated with vomiting, age, dysuria, abdominal pain, nappy use, and uncircumcision.

Conclusion: Our study found a relatively high proportion of UTIs among under-five children. *Escherichia coli* and *Staphylococcus aureus* were the commonly isolated organisms highly susceptible to nitrofurantoin, ciprofloxacin, meropenem, cefepime, and piperacillin-tazobactam, and least susceptible to commonly used antibiotics. Empirical treatment of UTI should be reviewed periodically based on the antibiotic susceptibility pattern.

Keywords: Urinary Tract Infection, Antimicrobial susceptibility pattern, under-fives

Introduction

Urinary tract infection (UTI) is common in under-five children presenting with fever with significant consequences leading to septicemia, dehydration, kidney scarring, and renal failure (1). Early diagnosis is essential to preserve renal functions of the kidney (2,3). After acute respiratory infections, UTI has been reported as the second most typical site of infection in the community, and incidence varies with age, gender, and health condition. The prevalence of UTI among under-five children ranges from 7.4 % to 43.6% (1,4,5). Several gram-negative and gram-positive bacteria have been reported as causative agents of UTI in children in different geographical locations (4,6,7). These agents have varied antibiotic susceptibility patterns, most resistant to commonly used antibiotics (4,8).

The risk factors for acquiring UTI in under-five children are multiple and differ with geographical locations, settings, and seasons. The commonly reported factors include female gender, poor hygiene, poverty, malnutrition, and recurrent infections (9,10). Other predisposing factors include age, uncircumcision (11), genitourinary anomalies, indwelling urinary catheters, and previous antibiotic use (12).

Urine culture is rarely performed as part of patient management in rural areas with inadequate diagnostic resources. Treatment is empirical, which could encourage the development and spread of antimicrobial-resistant bacteria (13). Antimicrobial resistance (AMR) has become a

significant concern worldwide. Recent research has shown a high prevalence of resistance to commonly used antibiotics (13–15). Antimicrobial resistance is also prevalent in Tanzania, with reports indicating multi-drug resistance among urinary pathogens associated with UTIs (16).

Extended-spectrum beta-lactamases (ESBL) producers contribute a high resistance rate to all beta-lactam antibiotics and are more common in hospitals than in the community or outpatient settings (16,17). ESBL-producing bacteria prevalence of infection or colonization among children under five years ranges from 17% to 35% (17–20). Studies in Tanzania reported that ESBL producers' prevalence ranges from 18% to 45%; Carbapenems were reported as the drugs to treat ESBL-producing bacteria (16,21). However, they are expensive and not widely available in Tanzania.

Therefore, increasing knowledge of the aetiologic profile and antimicrobial susceptibility patterns of pathogens associated with UTI in under-fives is essential in medical practices. Understanding the associated factors also helps identify at-risk groups and institute preventive measures. This research paper explored the etiologic profile, antimicrobial susceptibility pattern, and factors associated with UTI among under-five children in Magu District Hospital, Mwanza-Tanzania. The study findings will contribute to designing more targeted interventions to reduce the incidence of UTIs in Tanzania's children under five years old and review treatment guidelines.

Materials and Methods

Study design, setting, and population

We conducted a cross-sectional analytical study design between 8th March and 14th April 2023 at Magu District Hospital in Mwanza Region, North-Western Tanzania. Magu district is located between 33° and 34° East of Greenwich and between 2°10′ and 2°50′ South of the Equator. Its total area is 3075 km2, of which Lake Victoria waters cover 1725 km2 (56.1%). The average annual rainfall is between 700 and 1000 mm. As of 2012, the population of Magu district was 299,759 people. Magu District Hospital is a referral hospital in the district. Over 40% of the patient population seeking services is under-fives children. The laboratory procedures were conducted at Sekou Toure Regional Referral Hospital Laboratory. The study included under-five children with a fever seeking healthcare services. The study did not include

children who used catheters within 72 hours because catheter insertion may directly inoculate microorganisms into the urinary bladder.

Sample Size and Sampling Technique

We estimated the minimum sample size using the Kish Leslie formula considering the 11.4% previously reported prevalence of UTI among under-five children (22). A non-probability convenient sampling technique was used where all febrile under-five children attending Magu District Hospital were recruited in the study after obtaining consent from the parent/guardian.

Data collection

The participants' socio-demographic (age, sex, and place of residence), clinical characteristics (vomiting, dysuria, body temperature, abdominal pain, and chronic disease), and risk factors (nutrition status, history of recurrent UTI, male circumcision, duration of fever, and nappy use) were collected using a structured and pre-tested questionnaire. Axillary temperature was measured using a digital thermometer and recorded. The children's body weight was measured using a digital weighing scale. The nutrition status of the children was determined by calculating their weight-for-age z-scores (WAZ), which represent how many standard deviations their weight is from the median weight of a healthy reference population of the same age and sex. To assess their nutritional status, a WAZ below -3 standard deviations was used to indicate malnutrition, while a WAZ between -2 and +2 standard deviations was used to indicate a normal nutritional status (well-nourished) (23).

Sample collection

A urine specimen for each participant was collected. After receiving adequate instruction, the study participant's parents or guardians assisted with the urine collection. Approximately 10ml of urine specimens were collected into a sterile, wide-mouth, leak-proof, screw-capped plastic container pre-labelled with the participant’s identification number, age, date, and time. The specimen was transported daily to Sekou Toure Regional Referral Hospital laboratory for processing within two hours and kept in a cool box. Immediate inoculation of samples was done on arrival at the laboratory. If a delay was inevitable, specimens were refrigerated at 4°C until time for processing.

Urine Culture

We performed a quantitative urine culture to determine the significant bacteriuria. Urine samples were inoculated into Cysteine Lactose-Electrolyte Deficient (CLED) (Sigma Alorich, India) agar plates using a calibrated wire loop (0.001mL). Culture plates were incubated under aerobic conditions at 37°C for 24 hours. Bacterial growth of $\geq 10^5$ colony-forming unit (CFU)/mL of urine was considered significant bacteriuria.

Bacteria identification

We used conventional methods to Identify isolated bacteria, including colony characteristics, Gram staining ~~reaction,~~ and biochemical testing. Sulphur Indole Motility (SIM) (Himedia, India), Kligler Iron Agar test (Oxoid Ltd, UK), urease test (Oxoid Ltd, UK), and citrate utilisation test (Oxoid Ltd, UK) were used to identify Gram-negative bacteria. Catalase and coagulase tests were used to identify Gram-positive bacteria.

Antimicrobial susceptibility testing

Antimicrobial susceptibility testing was performed using the Kirby-Bauer disk diffusion method adhering to Clinical and Laboratory Standards Institute (CLSI) guidelines (24). Antibiotic disks for Gram-positive bacteria were penicillin G (10 μg), nitrofurantoin (300 μg), erythromycin (15 μg), ciprofloxacin (5μg), trimethoprim-sulfamethoxazole (1.25/23.75 μg), gentamicin (10 μg), cefoxitin (30 μg), and clindamycin (30 μg) (Himedia, India).

The antimicrobial disks for Enterobacteriaceae included piperacillin-tazobactam (100/10 μg), ampicillin (10 μg), nitrofurantoin (300 μg), amoxicillin-clavulanate (20/10 μg), gentamicin (10 μg), cefotaxime (30 μg), ciprofloxacin (30 μg), meropenem (10 μg), piperacillin (100 μg), cefepime (30 μg), amikacin (30 μg), trimethoprim-sulfamethoxazole (1.25/23.75 μg), ceftazidime (30 μg), and ceftriaxone (30 μg) (Himedia, India). Antimicrobial disks for *Pseudomonas* spp and *Acinetobacter* were gentamicin (10 μg), meropenem (10 μg), ceftazidime (30 μg), piperacillin-tazobactam (100/10 μg), ciprofloxacin (5 μg), and amikacin (30 μg) (Himedia, India).

Extended-Spectrum Beta-Lactamase Detection

Gram-negative bacteria producing ESBL was first suspected from the result of antimicrobial susceptibility testing based on the diameter of the zone of inhibition for one of the three third-

generation cephalosporins: cefotaxime (30μg), ceftriaxone (30μg) or ceftazidime (30μg). The following breakpoints were used to suspect ESBL production: ceftriaxone ≤ 25mm, ceftazidime ≤ 22mm, and cefotaxime ≤ 27mm.

We confirmed ESBL production using the Double Disk Synergy method (25). Ceftazidime (30μg), cefotaxime (30μg), and amoxicillin-clavulanic acid (20/10 μg) disks (Himedia, India) were placed in a straight line 20 mm apart on the plate, with amoxicillin-clavulanic acid in the centre. Any increase in the inhibition zone towards the amoxicillin-clavulanic acid disk was a positive result for ESBL enzyme production; the isolate was reported as an ESBL producer.

Detection of methicillin-resistant *Staphylococcus aureus*

Methicillin-resistant *Staphylococcus aureus* (MRSA) was phenotypically determined using a cefoxitin (30 μg) disk (Oxoid Ltd, UK) per CLSI guidelines (24). A cefoxitin disk was applied and incubated at 37^0C for 18 to 24 hours. A less or equal to 21 mm inhibition zone diameter around the cefoxitin disk indicated MRSA.

Quality assurance

Media and reagents were checked by verifying the expiration date, storage conditions, and visual inspection of any signs of contamination, such as discolouration, turbidity, or particulate matter, as indicated in the guidelines. The media quality was assessed using sterility and performance tests using reference strains. Positive reference strains for culture and susceptibility testing included *P. aeruginosa* (ATCC 27853), *S. aureus* (ATCC 25923), and *E. coli* (ATCC 25922). Additionally, *E. coli* (ATCC 25922) and *K. pneumonia* (ATCC 700603) ESBL-positive strains were used to control ESBL production. *S. aureus* (ATCC 43300) was used as a positive control for MRSA.

Data analysis

Data analysis was done using STATA version 15 StataCorp.2017. Frequency and proportions were used to summarise the characteristics of study populations for categorical variables, whereas median and interquartile range were used to summarise continuous variables. Pearson's chi-square test was used to compare a categorical variable to the status of UTI. The modified Poisson model was performed to determine factors associated with UTI. The multivariable analysis involved variables with a p-value ≤ 0.2 to determine the adjusted

prevalence ratio and 95% confidence intervals. A p-value < 0.05 was used in both models to acknowledge statistical significance.

Ethical approval

The study obtained ethical clearance from the Muhimbili University of Health and Allied Sciences Research and Ethics Committee. In addition, we got permission for data collection from the offices of the Regional and District Medical Officers. Parents or guardians were informed about the study objectives and consented on behalf of their children before inclusion in the study. Results of urine culture and antimicrobial susceptibility tests were timely communicated to the attending clinician for patient management. Participant identification numbers were used in the study instead of names to ensure confidentiality.

Results

Socio-demographic and clinical characteristics

A total of 368 under-five children were enrolled in the study. Males counted for more than half of the participants, 194 (52.7%) participants. The study participants' median age (IQR) was 24 (13-36) months. Most participants, 296 (80.4%), lived in rural areas. Malnutrition was observed in 27 (7.3%) and 29 (14.9%) males were already circumcised. Most participants, 201 (54.6%), had a fever for 3 to 7 days, and the majority, 348 (94.6%), had a 38-39 ^{0}C temperature. Of 368 participants, 153 (41.6%) had abdominal pain, while 61 (16.6%) had dysuria. Vomiting was observed in 84 (22.8%) participants, and 24 (6.5%) had a history of recurrent UTI (Table 1).

The proportion of UTI among under-five children

UTI was detected in 106 (28.8%), more in children below 24 months, 53 (35.1%), than in children aged 24 months and above, 53 (24.4%) (p =0.026). UTI was significantly higher in children with dysuria, 30 (49.2%) compared to those without dysuria, 76 (24.8%) (p < 0.001). The proportion of UTI was higher among children with a fever of 3-7 days 68 (33.8%) compared to those with a fever of 0-2 days 33 (21.3%) and greater than 7 days 5 (41.7%) (p=0.021). Children presented with abdominal pain were more diagnosed with UTI, 81 (52.9%), than those without abdominal pain, 25 (11.6%) (p< 0.001). The proportion of UTI was found to be higher among children who used nappies, 56 (34.8%), than in children who did not use nappies, 50 (24.2%) (p=0.026). Uncircumcised male children had more UTI, 55 (33.3%) than

circumcised children, 3 (10.3%) (p=0.030). The proportion of UTI was significantly higher in children presented with vomiting 37 (44.0%) than in those without vomiting 69 (24.3%) (p< 0.001). The proportion of UTI was higher in children with a body temperature above 39°c 11 (55.0%) than in children with a body temperature between 38-39 ^{O}C, 95 (27.3%) (p=0.008). There was no difference in UTI proportion based on sex, history of recurrent UTI, place of residence, nutrition status, or chronic disease, and the p-value was greater than 0.05 (Table 1).

Table 1: Proportion of UTI among the febrile under-five children attending Magu District Hospital in Mwanza region, March to April 2023

Variable	Description	Frequency N (%) *	UTI positive n (%) **	P value
**Overall**		368	106(28.8)	
**Age group (months)**	<24	151(41.0)	53 (35.1)	0.026
	≥24	217(59.0)	53 (24.4)	
**Sex**	Male	194 (52.7)	58 (29.9)	0.625
	Female	174 (47.3)	48 (27.6)	
**Duration of fever (days)**	0.2	155 (42.1)	33 (21.3)	0.021
	3-7	201 (54.6)	68 (33.8)	
	>7	12 (3.3)	5 (41.7)	
**Axillary Temperature**	38-39	348 (94.6)	95 (27.3)	0.008
	≥39	20 (5.4)	11 (55.0)	
**Dysuria**	Yes	61 (16.6)	30 (49.2)	< 0.001
	No	307 (83.4)	76 (24.8)	
**Vomiting**	Yes	84 (22.8)	37 (44.0)	< 0.001
	No	284 (77.2)	69 (24.3)	
**Abdominal pain**	Yes	153 (41.6)	81 (52.9)	< 0.001
	No	215 (58.4)	25 (11.6)	
**Recurrent UTI**	Yes	24 (6.6)	7 (29.2)	0.968
	No	344 (93.5)	99 (28.8)	
**Use of nappy**	Yes	161 (43.8)	56 (34.8)	0.026
	No	207 (56.2)	50 (24.2)	
**Chronic disease**	Yes	8 (2.2)	2 (25.0)	0.810
	No	360 (97.8)	104 (28.9)	
**Nutrition status**	Malnourished	27 (7.3)	10 (37.0)	0.326
	Well-nourished	341 (92.7)	96 (28.2)	
**Male circumcision (n = 194)**	Yes	29 (14.9)	3 (10.3)	0.030
	No	165 (85.1)	55 (33.3)	
**Place of residence**	Urban	72 (19.6)	20 (27.8)	0.830
	Rural	296 (80.4)	86 (29.1)	

*UTI=Urinary Tract Infection, *Frequency and percentage of participants calculated as number within the variable category divided by the total sample size of the variable,*

*** proportion of patients within the variable category with Urinary Tract Infection calculated as number of cases divided by the total number of individuals in the variable category*

Bacteria causing UTI among under-five children

A total of 106 pathogenic bacteria strains were isolated from 106 urine specimens of febrile under-five children. Gram-negative bacteria constituted 80 (75.48%), while Gram-positive bacteria counted 26 (24.52%). *E. coli* was the most common isolated pathogen 37 (35%), followed by *Staphylococcus aureus* 26/106 (25%) (Figure 1).

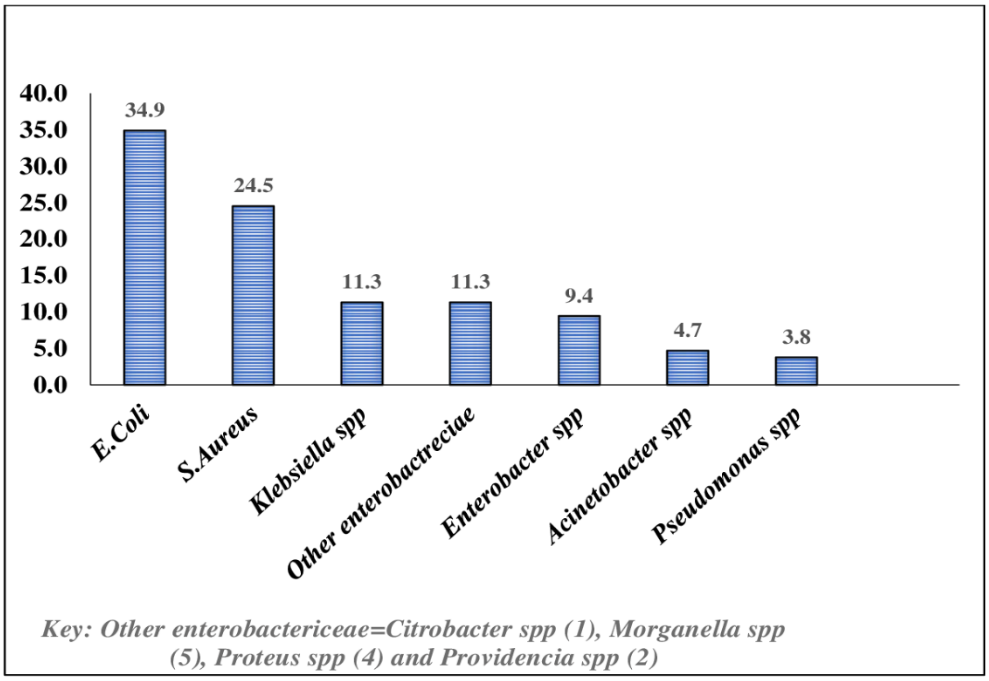

Figure 1: Proportion of bacteria isolates among febrile children under five years attending Magu District Hospital in Mwanza region, March to April 2023

Factors Associated with Urinary Tract Infection

In univariate analysis, age, vomiting, dysuria, body temperature, abdominal pain, use of nappy, and uncircumcision were associated with UTI. Febrile children below 24 months, used nappies, and those presented with vomiting, dysuria, and body temperature above 39°C were more likely to have UTI than their counterparts, with the prevalence ranging between 1.4 and 2.0, $p < 0.05$. Children with abdominal pain had 4.6 times more UTI than those without (CPR 4.6; 95%CI:3.1-6.8, $p < 0.001$). Uncircumcised children had 3.2 times more chances of acquiring UTI than circumcised (CPR 3.2; 95% CI: 1.1–9.6, P = 0.035). Sex, duration of fever, history of recurrent UTI, place of residence, nutrition status, and a history of having chronic disease were not associated with UTI.

Eight factors were included in the multivariable analysis. Age below 24 months, abdominal pain, vomiting, dysuria, body temperature above 39°C, use of nappy, and uncircumcision were independently associated with UTI. Children below 24 months had 1.5 times the prevalence of acquiring UTI compared to those aged 24 months and above (APR= 1.5, 95% CI = 1.1–2.1, P = 0.010). Furthermore, children with abdominal pain had 3.9 times the prevalence of having a UTI than those without abdominal pain (APR = 3.9, 95% CI = 2.6–5.9, P < 0.001). Having vomiting, dysuria, or a body temperature above 39°C increased the prevalence of having UTI by 1.6 to 1.7 times. Using a nappy increased the prevalence of acquiring UTI by 1.2 times more than not using a nappy (APR 1.2; 95% CI: 0.6–2.4, P = 0.003). Uncircumcised children had 2.5 times the prevalence of acquiring UTI than circumcised (APR 2.5; 95% CI: 0.4–8.2, P = 0.002) (Table 2).

Table 2: Factors associated with UTI among febrile children under five years in Magu District -Mwanza region, March to April 2023

**Characteristic**	**Total**	**UTI**	**Bivariate analysis**		**Multivariate analysis**	
		**n (%)**	**CPR (95% CI)**	**P value**	**APR (95%CI)**	**P value**
**Age group (months)**						
<24	151	53 (35.1)	1.4 (1.0-1.9)	0.030	1.5 (1.1-2.1)	0.010
≥24	217	53 (24.4)	1			
**Sex**						
Male	194	58 (29.9)	1.0 (0.8-1.5)	0.625		
Female	174	48 (27.6)	1			
**Duration of fever (days)**						
0.2	155	33 (21.3)	1			
3-7	201	68 (33.8)	1.5 (1.1-2.1)	0.020	1.4 (0.9-1.9)	0.057
>7	12	5 (41.7)	1.5 (0.7-2.9)	0.275	1.5 (0.7-2.9)	0.179
**Temperature**						
38-39	348	95 (27.3)	1			
≥39	20	11 (55.0)	2.0 (1.3-3.1)	0.002	1.7 (1.1-2.8)	0.020
**Dysuria**						
YES	61	30 (49.2)	1.9 (1.4-2.7)	< 0.001	1.6 (1.2-2.3)	0.010
NO	307	76 (24.8)	1			
**Vomiting**						
YES	84	37 (44.0)	1.8 (1.3-2.5)	< 0.001	1.6 (1.2-2.2)	0.002
NO	284	69 (24.3)	1			
**Abdominal pain**						
YES	153	81 (52.9)	4.6 (3.1-6.8)	< 0.001	3.9 (2.6-5.9)	< 0.001
NO	215	25 (11.6)	1			
**Recurrent UTI**						
YES	24	7 (29.2)	1.0 (0.5-1.9)	0.968		
NO	344	99 (28.8)	1			
**Use of nappy**						
YES	161	56 (34.8)	1.4 (1.0-1.9)	0.026	1.2 (0.6-2.4)	0.003
NO	207	50 (24.2)	1			
**Chronic disease**						
YES	8	2 (25.0)	0.9 (0.3-2.9)	0.815		
NO	360	104 (28.9)	1			

**Nutrition status**						
Malnourished	27	10 (37.0)	1.3 (0.8-2.2)	0.302		
Well-nourished	341	96 (28.2)	1			
**Male circumcision** (n = 194)						
YES	29	3 (10.3)	1			
NO	165	55 (33.3)	3.2 (1.1-9.6)	0.035	2.5 (0.4-8.2)	0.002
**Place of residence**						
Urban	72	20 (27.8)	1			
Rural	296	86 (29.1)	1.0 (0.7-1.6)	0.831		

UTI: urinary tract infection, CPR: Crude Prevalence Ratio, APR: Adjusted Prevalence Ratio

Antimicrobial susceptibility patterns of isolated bacteria

Escherichia coli were highly susceptible to cefepime, piperacillin-tazobactam, nitrofurantoin, and meropenem, ranging from 81.1% to 97.3%, while least susceptible to Trimethoprim-sulfamethoxazole (37.8%), Gentamycin (32.4%), and Ampicillin (18.9%). *Staphylococcus aureus* was highly susceptible to nitrofurantoin (96.2%) and ciprofloxacin (92.3%), and least susceptible to penicillin (11.5%) and erythromycin (19.2%). Susceptibility to ciprofloxacin ranges from 40% (*Acinetobacter* spp) to 100% *Pseudomonas aeruginosa*, *Citrobacter* spp, *Morganella morganii*, *Proteus* spp, and *Providencia* spp. Susceptibility to Meropenem ranges from 20% *Acinetobacter* to 100% *Enterobacter* species. SXT susceptibility ranges from (37.8% *E. coli)* to 70% (*S. aureus)* (Table 3).

Table 3: Antimicrobial Susceptibility patterns of bacteria causing UTI among children under five years attending Magu District Hospital in Mwanza region, March to April 2023

	Isolated organism N (%)						
**Antibiotics**	***E. coli=37***	***S. a=26***	***K. spp n=12***	***E.spp n=10***	***P. a=4***	***A.spp=5***	***OE=12***
Nitrofurantoin	32(86.5)	25(96.2)	11 (91.7)	8(80)	NA	NA	7 (58)
Ciprofloxacin	28(75.7)	24(92.3)	10 (83.3)	9(90)	4(100)	2(40)	12 (100)
Penicillin	NA	3(11.5)	NA	0(0)	NA	NA	NA
Erythromycin	NA	5(19.2)	NA	0(0)	NA	NA	NA
SXT	14(37.8)	14(70)	8 (66.7)	6(60)	NA	NA	7 (58)
Gentamycin	12(32.4)	19(73)	3 (25.0)	3(30)	0	1(20)	3 (25)
Ampicillin	7(18.9)	NA	0.0	1(10)	NA	NA	1 (8)
Cefoxitin	NA	20(76.9)	NA	0(0)	NA	NA	NA
Clindamycin	NA	20(76.9)	NA	0(0)	NA	NA	NA
AMC	25(67.6)	NA	7 (58.3)	9(90)	NA	NA	6 (50)
Cefotaxime	25(67.5)	NA	6 (50.0)	6(60)	NA	NA	4 (33)
Ceftriaxone	29(78.4)	NA	7 (58.4)	6(60)	NA	NA	7 (58)
Ceftazidime	27(72.9)	NA	8 (66.7)	9(90)	2(50)	1(20)	9 (75)
Meropenem	36(97.3)	NA	11 (91.7)	10(100)	1(25)	1(20)	12 (100)
Cefepime	30(81.1)	NA	10 (83.3)	8(80)	4(100)	1(20)	8 (67)

Piperacillin	23(62.2)	NA	5 (41.7)	8(80)	2(50)	3(60)	8 (67)
TZP	32(86.5)	NA	10 (83.4)	9(90)	2(50)	3(60)	12 (100)
Amikacin	28(75.7)	NA	8 (66.7)	9(90)	4(100)	4(80)	9 (75)

S. a = Staphylococcus aureus, K. Spp = Klebsiella species, E. Spp = Enterobacter species, P. a = Pseudomonas aeruginosa, A.spp = *Acinetobacter species*, SXT = Trimethoprim-sulfamethoxazole, TZP = Piperacillin Tazobactam, OE (Other Enterobacteriaceae) = (*Citrobacter* spp, *Morganella morganii, Proteus* spp, and *Providencia* spp), NA = Not Applicable

Multi-drug resistant bacterial strains causing UTI

MDR was observed in 35/106 (33.0%) isolated organisms. A high MDR above 50% was observed in *Acinetobacter* spp (80%), *Pseudomonas aeruginosa* (75.0%), and *Klebsiella* spp (58.3%). The MRSA was detected in 6/26 (23.1%) of the *S. aureus*. ESBL production was detected in 19/76 (25%) of Gram-negative bacteria. The proportion of ESBL production was highest in *Acinetobacter* spp (80.0%) and lowest in *Enterobacter* spp (10.0%) (Table 4).

Table 4: MDR, ESBL, and MRSA isolates recovered from the urine culture specimens of the febrile under-five children attending Magu District Hospital in Mwanza region, March to April 2023

Pathogen	Frequency	MDR, N (%)	ESBL, N (%)	MRSA, N (%)
S. aureus	26	11 (42.3)	NA	6 (23.1)
E. coli	37	7 (18.9)	7 (18.9)	NA
Klebsiella spp	12	7 (58.3)	5 (41.7)	NA
Enterobacter spp	10	1 (10.0)	1 (10.0)	NA
Acinetobacter spp	5	4 (80.0)	4 (80.0)	NA
Morganella Morganii	5	1 (20.0)	1 (20.0)	NA
Proteus spp	4	1 (25.0)	1 (25.0)	NA
P. aeruginosa	4	3 (75.0)	NA	NA
Overall		35/106 (33.0)	19/76(25.0)	6/26 (23.1)

MDR = Multi-Drug Resistant, ESBL = Extended Spectrum Beta Lactamase,
MRSA = Methicillin-Resistant *Staphylococcus aureus*

Discussions

Our study found that more children under five still acquire bacterial infections, with *S. aureus* and *E. coli* being the common etiological agents. Isolated bacteria were highly susceptible to nitrofurantoin, ciprofloxacin, meropenem, cefepime, and piperacillin-tazobactam. while commonly used antibiotics such as ampicillin, gentamycin, penicillin, and erythromycin were highly resistant. Our findings showed that a history of vomiting, dysuria, nappy use, uncircumcision, and abdominal pain were associated with Urinary Tract infection in febrile children under five years. This suggests that children under five are still susceptible to urinary tract infections, and various factors may increase the likelihood of UTI. Additionally,

commonly used antibiotics may not effectively treat the bacterial infections identified in this study.

Our finding on the proportion of UTI among febrile under-five children was lower than in previous studies conducted in Mwanza City (8), Bagamoyo (4), and Kilimanjaro (22) in Tanzania. However, this study reported a higher proportion of UTI than in Kenya (26), Nigeria (2), and Cameroon (6). Different risk factors can explain this variation between the current and previous studies among the population under the study, such as low hygienic standards and malnutrition (9). Additionally, only children with fever were included in this study, which may account for the higher prevalence than that conducted at Kilimanjaro, which included children with or without symptoms of UTI (22).

The common causative agent of UTI in under-five children was *E. coli,* followed by *Staphylococcus aureus*. Similar results were reported in previous studies conducted in Nigeria (2) and Cameroon (6). A study in Mwanza (8) on UTI in children also reported *E. coli* as the common isolate causing UTI. Another study at Muhimbili National Hospital, Dar es Salaam, Tanzania, reported *E. coli* as the common isolate, followed by *Klebsiella* spp, *and Staphylococcus aureus* (14). The bacterial spectrum obtained in this study is nearly identical to that conducted on the prevalence of UTI among children in Kilimanjaro, Tanzania, which identified *E. coli, Staphylococcus aureus, Klebsiella* spp, *Proteus*, and *Pseudomonas spp* as the common aetiology of UTI (22).

Most isolates were susceptible to nitrofurantoin, ciprofloxacin, meropenem, cefepime, and piperacillin tazobactam. These results agree with similar studies conducted in Mwanza (8), Kilimanjaro (22), and Dar es Salaam (14) in Tanzania and Nigeria (1) on the susceptibility patterns. The high susceptibility of isolated bacteria to meropenem may be attributed to the fact that the injectable beta-lactam antibiotic is not frequently available in Tanzanian pharmacies. As a result, meropenem is less routinely administered in Tanzania than other more widely available antibiotics. Ciprofloxacin, on the other hand, is not commonly used in children under the age of 12 years.

The current study shows that isolated bacteria were somewhat sensitive to ceftriaxone, amoxicillin-clavulanate, and ceftazidime, consistent with other studies conducted in Mwanza (8) and Dar es Salaam (14), Tanzania and Ethiopia (27). The findings suggest these commonly used antibiotics to treat various bacterial infections have variations of effectiveness against

certain bacteria. The susceptibility may vary depending on the specific strain and resistance mechanisms, particularly in the case of extended-spectrum beta-lactamase (ESBL)--producing organisms.

High resistance rates were observed to antibiotics commonly used in rural settings, including trimethoprim-sulfamethoxazole, piperacillin, ampicillin, gentamycin, penicillin, and erythromycin. These findings agree with results obtained from previous studies conducted in Mwanza (8) and Bagamoyo (4) in Tanzania and Kenya (26). However, different findings were reported in the study conducted at Muhimbili National Hospital that most *E. coli* were highly sensitive to gentamycin (14). The disparity in resistance rates may be attributed to the fact that these antibiotics are readily accessible at the primary healthcare level and are, therefore, being given empirically based on clinical features, accelerating antimicrobial resistance. The results of this study highlight the significance of performing a urine culture and an antimicrobial susceptibility test on febrile children.

The prevalence of ESBL-producing bacteria revealed in this study was lower than the other studies in Mwanza (28) and Dar es Salaam (21), Tanzania. The proportion of MRSA was also lower than in previous studies conducted in Nigeria (29) and India (30). Differences in geographical locations can explain lower proportions, as this study was conducted in rural areas where UTI is more likely to be community-acquired, whereas, in urban areas, UTI is more likely to be acquired at the hospital from possibly admitted patients.

The current study found that children under 24 months had a higher risk of acquiring a UTI than those older than 24 months. Similar findings were reported by previous studies conducted in Mwanza (8) and Kilimanjaro (22) in Tanzania. The finding may be explained by the fact that the age group is at risk due to underdeveloped immune systems, wearing of nappies, and urinary tract abnormalities (9). This study found that the risk of urinary tract infections (UTI) in non-circumcised children was high, consistent with previous studies conducted in Dhaka (9).

This study revealed that symptoms of vomiting, difficulty urination, body temperature greater or equal to 39 degrees centigrade, and abdominal pain were associated with UTI in febrile children under five years. These findings align with the previous studies conducted in Mwanza (8) and Nigeria (31,32). Similar study populations, risk factors, and environmental factors may have contributed to the consistency of these findings. This study also reported that a history of recurrent UTI, chronic disease, nutrition status, and duration of fever did not show a significant association with UTI in children under five years, which agrees with other reports from Nigeria

(1), England (2), and Kilimanjaro-Tanzania (22). This finding highlights the importance of considering additional risk factors and potential causes of UTI in febrile children.

We were unable to ascertain the prevalence of UTI and assess antimicrobial susceptibility patterns among children who had used antibiotics in the past two weeks due to challenges in recall bias.

Conclusion and recommendations

We observed a relatively high proportion of under-five children with UTI, mainly due to *Escherichia coli* and *Staphylococcus aureus*. The causative agents exhibited high susceptibility to simple drugs like nitrofurantoin but were least susceptible to commonly prescribed antibiotics for empirical treatment of UTI. Our study underscores the importance of periodic review of empirical treatment for UTIs based on the prevailing antibiotic susceptibility pattern**.**

Acknowledgements

We want to convey our sincere gratitude to the Management of Sekou-Toure Regional Referral Hospital and Magu District Hospital for allowing us to conduct our research under their purview. We thank Mr. Castory Dome, Mr. Philipo Shokolo, and Mr. Leoncy Kapinga for their technical assistance and directives during data and specimen collection. we express our gratitude to respondents who willingly participated and cooperated fully, thus facilitating the successful completion of this study.

Author Contributions

Conceptualization: RE, NL, MM

Data Curation: RE

Formal Analysis: RE, MM

Investigation: RE, NL, MM

Methodology: RE, NL, MM

Supervision: NL, SY, AJ, MM

Validation: RE, NL, SY, AJ, MM

Visualization: RE, NL, SY, AJ, MM

Writing – Original Draft Preparation: RE, NL, MM

Writing – Review & Editing: RE, NL, SY, AJ, MM

Abbreviations

ATCC: American Type Culture Collection, CFU: Colony-Forming Unit, CLSI: Clinical and Laboratory Standards Institute, ESBL: Extended Spectrum Beta Lactamase, MDR: Multi-Drug Resistant, MRSA: Methicillin-Resistant *Staphylococcus Aureus*, UTI: Urinary Tract Infection, WHO: World Health Organization.

References

1. Ibeneme CA, Oguonu T, Okafor HU, Ikefuna AN, Ozumba UC. Urinary tract infection in febrile under five children in Enugu , South Eastern Nigeria. 2014;17(5):624–8.
2. Westwood ME, Whiting PF, Cooper J, Watt IS, Kleijnen J. Further investigation of confirmed urinary tract infection (UTI) in children under five years : a systematic review. 2005;10:1–10.
3. Brien KO, Stanton N, Edwards A, Hood K, Butler CC. Prevalence of urinary tract infection (UTI) in sequential acutely unwell children presenting in primary care in South Wales (UK): Exploratory. 2011;3432.
4. Sangeda RZ, Paul F, Mtweve DM. Prevalence of urinary tract infections and antibiogram of uropathogens isolated from children under five attending Bagamoyo District Hospital in Tanzania : A cross-sectional study [version 1 ; peer review : 1 not approved]. 2022;1–13.
5. Alex Fortunatus, Magufwa. Epidemiology of Urinary Tract Infection among febrile Children under five years in morogoro municipality, Tanzania. 2016;
6. Francine D, Motse K, Ngaba GP, Christiane D, Pradel L, Foko K. Etiologic Profile and Sensitivity Pattern of Germs Responsible for Urinary Tract Infection Among Under-five Children in Douala , Cameroon : A Hospital-Based Study. 2019;6(2):49–56.
7. Dm K, Mosha F, Moyo S, Mi M. Etiology and Antimicrobial Susceptibility Patterns of Bacterial Agents Causing Urinary Tract Infection in Children under Five years , dar es Salaam . 2020;2(1).
8. Msaki BP, Mshana SE, Hokororo A, Mazigo HD, Morona D. Prevalence and predictors of urinary tract infection and severe malaria among febrile children attending Makongoro health centre in Mwanza city , North-Western Tanzania. 2012;1–8.
9. Ferdaus J, Moinuddin G, Islam MT, Rashid MHO, Shawly MR, Alim A. Risk Factors of urinary tract infection in preschool children in Dhaka, Bangladesh. Bangladesh Med J. 2017;45(3):134–7.
10. Mohamed F. Prevalence and factors associated with urinary tract infection among febrile children admitted at Dodoma regional referral hospital. 2019;
11. Nader Shaikh, Natalia E. Morone, James E and Max H. Farrell. Prevalence of Urinary Tract Infection in Childhood. 2008;27(4):302–8.
12. Mekonnen S, Tesfa T, Shume T, Tebeje F, Urgesa K, Weldegebreal F. Bacterial profile , their antibiotic susceptibility pattern , and associated factors of urinary tract infections in children at Hiwot Fana Specialized University Hospital , Eastern. 2023;(Ci):1–21.
13. Aust MJ, Intern A. Antimicrobial resistance in developing countries. 1998;647–50.

14. Fredrick F, Francis JM, Fataki M, Maselle SY. Aetiology , antimicrobial susceptibility and predictors of urinary tract infection among febrile under-fives at Muhimbili National Hospital , Dar es Salaam-Tanzania. 2013;7(12):1029–34.

15. Jiya N M, FWACP, Ibitoye PK, FWACP, Jiya F B M. Urinary tract infection and malaria co-morbidity in febrile children with sickle-cell anaemia in Sokoto, Nigeria 2012;20(1):299–305.

16. The GARP-Tanzania Working Group Said Aboud, MD, PhD, Chairman Robinson Mdegela, BVSc, PhD V. SITUATION ANALYSIS AND RECOMMENDATIONS Antibiotic Use and Resistance in Tanzania The Global Antibiotic Resistance Partnership—Tanzania Work Group 2015 2015;(March):1–83.

17. Alemu M, Id T, Abera NA, Gebeyehu YM. High prevalence of extended-spectrum beta-lactamase-producing Escherichia coli and Klebsiella pneumoniae fecal carriage among children under five years in Addis Ababa ,. 2021;1–16.

18. Letara N, Ngocho JS, Karami N, Msuya SE. Prevalence and patient related factors associated with Extended‐Spectrum Beta‐Lactamase producing Escherichia coli and Klebsiella pneumoniae carriage and infection among pediatric patients in Tanzania 2021;1–9.

19. Onduru OG, Mkakosya RS, Rumisha SF, Aboud S. Carriage Prevalence of Extended-Spectrum β -Lactamase Producing Enterobacterales in Outpatients Attending Community Health Centers in Blantyre , Malawi. 2021;

20. Birgy A, Cohen R, Levy C, Bidet P, Courroux C, Benani M. Community faecal carriage of extended-spectrum beta-lactamase-producing Enterobacteriaceae in french children. 2012;1–5.

21. Tellevik MG, Kommedal Ø, Maselle SY, Langeland N, Moyo SJ. High Prevalence of Faecal Carriage of ESBL- Producing Enterobacteriaceae among Children in Dar es Salaam , Tanzania. 2016;1–13.

22. Joshua G, Rune Philemon, Mohammed S Abdallah, Aliasgher M Saajan, Theresia Temu, Ipyana Kunjumu, Blandina T Mmbaga. Patterns of Urinary Tract Infection Among Children Admitted at Kilimanjaro Christian Medical Centre ,. 2017;1(1):53–61.

23. Assessment N. How to Calculate Weight-for-Height Z-Score (WHZ) in C hildren 0 − 59 Months of Age. :6–9.

24. CLSI. M100-ED32 CC:2022. Performance Standards for Antimicrobial Susceptibility Testing. 2022.

25. Abdelmoktader A, Talal A, Far E. Methods of ESBLs Detection in Clinical Microbiology Lab. 2019;1–8.

26. Masika WG, Meara WPO, Holland TL, Armstrong J. Contribution of urinary tract infection to the burden of febrile illnesses in young children in rural Kenya. 2017;1–13.

27. Marami D, Balakrishnan S, Seyoum B. Prevalence, Antimicrobial Susceptibility Pattern of Bacterial Isolates, and Associated Factors of Urinary Tract Infections among HIV-Positive Patients at Hiwot Fana Specialized University Hospital, Eastern Ethiopia. Can J Infect Dis Med Microbiol. 2019;2019.

28. Ahmed M, Moremi N, Mirambo MM, Hokororo A, Mushi MF, Seni J. Multi-resistant gram negative enteric bacteria causing urinary tract infection among malnourished underfives admitted at a tertiary hospital , northwestern , Tanzania. 2015;1–5.

29. Abubakar U, Usman Sulaiman, Syed AS. Prevalence, trend and antimicrobial

susceptibility of Methicillin Resistant Staphylococcus aureus in Nigeria 2018;11(6):763–70

30. Arora S, Devi P, Arora U, Devi B. Prevalence of Methicillin-resistant Staphylococcus Aureus (MRSA) in a Tertiary Care Hospital in Northern India. 2020;(2):2–5.

31. Okunola P, Ibadin M, Ofovwe G. Predicting Urinary Tract Infection in Malaria Co-morbidity among Under Fives. Niger Hosp Pract. 2011;7(3–4).

32. Estefanía J, Regalado A, Brito MO, Enrique L. Prevalence of urinary tract infection and associated factors in hospitalized patients aged 0 to 5 years . 2021;22(1):1–8.
