# Supplementary material for: Bacterial aetiology, antimicrobial susceptibility patterns, and factors associated with urinary tract infection among under-five children at primary health facility, North-Western Tanzania
Source: PLoS One. 2024 May 6;19(5):e0303369. doi: 10.1371/journal.pone.0303369 (PMC11073726; doi:10.1371/journal.pone.0303369)
Supplement: Attachment — Submitted filename: UTI second comment.pdf [file pone.0303369.s004.pdf]

PLOS ONE

Bacterial etiologic Profile, Antimicrobial Susceptibility Patterns, and Factors Associated with Urinary Tract Infection among Under-five Children at Primary Health Facility, North-Western Tanzania

--Manuscript Draft--

**Manuscript Number:**	PONE-D-23-32229R1
**Article Type:**	Research Article
**Full Title:**	Bacterial etiologic Profile, Antimicrobial Susceptibility Patterns, and Factors Associated with Urinary Tract Infection among Under-five Children at Primary Health Facility, North-Western Tanzania
**Short Title:**	Bacterial Profile, Antimicrobial Susceptibility Patterns, and Factors Associated with Urinary Tract Infection
**Corresponding Author:**	Roza Ernest<br>MUHAS: Muhimbili University of Health and Allied Sciences<br>Dar es Salaam, TANZANIA, UNITED REPUBLIC OF
**Keywords:**	urinary tract infection; Antimicrobial susceptibility pattern; under-fives
**Abstract:**	Abstracts<br><br>Background: Urinary tract infection is common in under-five children presenting with fever with significant consequences leading to bacteremia, dehydration, kidney scarring, and renal failure. The incidence of Urinary tract infection varies with age, gender, health condition, and geographic location. There is a lack of studies addressing these issues, particularly concerning children. This study determined the proportion of urinary tract infections, bacterial aetiology, antimicrobial susceptibility patterns, and factors associated with urinary tract infection among under-five children.<br><br>Materials and methods : The cross-sectional analytical study was conducted at the District Hospital in Mwanza Region, North-Western Tanzania, between March and April 2023. A convenient non-probability sampling technique was used to collect urine samples from children under five years old with signs and symptoms of urinary tract infection. Written informed consent was obtained from all parents or guardians. Participants' information was collected using a pretested structured questionnaire. Urine samples were processed at Sekou Toure Regional Referral Hospital. STATA version 15.0 was used for data analysis. Factors associated with Urinary tract infection were determined using a Modified Poisson Model.<br><br>Result: The study recruited 368 under-five children; 194 (52.7%) were males. The median age (interquartile range) was 24 (13-36) months. Of all, 28.8% (95% CI-24.3-33.6) had urinary tract infections. One hundred and six pathogens were isolated, the majority being Escherichia coli, 37 (34.9%), and Staphylococcus aureus, 26 (24.5%).Escherichia coli were susceptible to cefepime, piperacillin-tazobactam, nitrofurantoin, and meropenem, ranging from 81.1% to 97.3%. Staphylococcus aureus was susceptible to nitrofurantoin (96.2%) and ciprofloxacin (92.3%). Multidrug resistance was observed in 33.0% of isolates, Methicillin-Resistance Staphylococcus aureus in 23.1% of Staphylococcus aureus, and extended-spectrum beta-lactamases in 25% of gram-negative bacteria. Urinary tract infection was associated with vomiting, age, dysuria, abdominal pain, nappy use, and uncircumcision in males.<br>Conclusion: Our study found a relatively high proportion of urinary tract infections among under-five children. Escherichia coli and Staphylococcus aureus were the common isolated organisms susceptible to nitrofurantoin, ciprofloxacin, meropenem, cefepime, and piperacillin-tazobactam, and least susceptible to commonly used antibiotics. Empirical treatment of urinary tract infection should be reviewed periodically based on the antibiotic susceptibility pattern.
**Order of Authors:**	Roza Ernest
	Nsiande Lema
	Sued Yassin

Powered by Editorial Manager® and ProduXion Manager® from Aries Systems Corporation

	Agricola Joachim
	Mtebe Majigo

Response to Reviewers:

Editor's comments
When submitting your revision, we need you to address these additional requirements.
1. Please ensure that your manuscript meets PLOS ONE's style requirements, including those for file naming. The PLOS ONE style templates can be found at https://journals.plos.org/plosone/s/file?id=wjVg/PLOSOne_formatting_sample_main_body.pdf and https://journals.plos.org/plosone/s/file?id=ba62/PLOSOne_formatting_sample_title_authors_affiliations.pdf

Response: We have tried our best to write the manuscript following PLOS ONE's style

2. We suggest you thoroughly copyedit your manuscript for language usage, spelling, and grammar. If you do not know anyone who can help you do this, you may wish to consider employing a professional scientific editing service. Whilst you may use any professional scientific editing service of your choice, PLOS has partnered with both American Journal Experts (AJE) and Editage to provide discounted services to PLOS authors. Both organizations have experience helping authors meet PLOS guidelines and can provide language editing, translation, manuscript formatting, and figure formatting to ensure your manuscript meets our submission guidelines. To take advantage of our partnership with AJE, visit the AJE website (http://learn.aje.com/plos/) for a 15% discount off AJE services. To take advantage of our partnership with Editage, visit the Editage website (www.editage.com) and enter referral code PLOSEDIT for a 15% discount off Editage services. If the PLOS editorial team finds any language issues in text that either AJE or Editage has edited, the service provider will re-edit the text for free. Upon resubmission, please provide the following: The name of the colleague or the details of the professional service that edited your manuscript. A copy of your manuscript showing your changes by either highlighting them or using track changes (uploaded as a *supporting information* file). A clean copy of the edited manuscript (uploaded as the new *manuscript* file)"

Response: The manuscript has been thoroughly checked for language usage, spelling and grammar using Grammarly Premium

3. In your Data Availability statement, you have not specified where the minimal data set underlying the results described in your manuscript can be found. PLOS defines a study's minimal data set as the underlying data used to reach the conclusions drawn in the manuscript and any additional data required to replicate the reported study findings in their entirety. All PLOS journals require that the minimal data set be made fully available. For more information about our data policy, please see http://journals.plos.org/plosone/s/data-availability. Upon re-submitting your revised manuscript, please upload your study's minimal underlying data set as either Supporting Information files or to a stable, public repository and include the relevant URLs, DOIs, or accession numbers within your revised cover letter. For a list of acceptable repositories, please see http://journals.plos.org/plosone/s/data-availability#loc-recommended-repositories. Any potentially identifying patient information must be fully anonymized. Important: If there are ethical or legal restrictions to sharing your data publicly, please explain these restrictions in detail. Please see our guidelines for more information on what we consider unacceptable restrictions to publicly sharing data: http://journals.plos.org/plosone/s/data-availability#loc-unacceptable-data-access-restrictions. Note that it is not acceptable for the authors to be the sole named individuals responsible for ensuring data access. We will update your Data Availability statement to reflect the information you provide in your cover letter.

Response: The study's minimal underlying data set has been uploaded as a Supporting Information files

4. We note that you have indicated that data from this study are available upon request. PLOS only allows data to be available upon request if there are legal or ethical restrictions on sharing data publicly. For more information on unacceptable data access restrictions, please see http://journals.plos.org/plosone/s/data-availability#loc-

Powered by Editorial Manager® and ProduXion Manager® from Aries Systems Corporation

unacceptable-data-access-restrictions. In your revised cover letter, please address the following prompts:
a) If there are ethical or legal restrictions on sharing a de-identified data set, please explain them in detail (e.g., data contain potentially sensitive information, data are owned by a third-party organization, etc.) and who has imposed them (e.g., an ethics committee). Please also provide contact information for a data access committee, ethics committee, or other institutional body to which data requests may be sent.
b) If there are no restrictions, please upload the minimal anonymized data set necessary to replicate your study findings as either Supporting Information files or to a stable, public repository and provide us with the relevant URLs, DOIs, or accession numbers. For a list of acceptable repositories, please see http://journals.plos.org/plosone/s/data-availability#loc-recommended-repositories.
We will update your Data Availability statement on your behalf to reflect the information you provide.
Response: The study's minimal underlying data set has been uploaded as a Supporting Information files

5. Please include your full ethics statement in the 'Methods' section of your manuscript file. In your statement, please include the full name of the IRB or ethics committee who approved or waived your study, as well as whether or not you obtained informed written or verbal consent. If consent was waived for your study, please include this information in your statement as well.

Response: The study obtained ethical clearance number MUHAS-REC-12-2022-1484 from the Muhimbili University of Health and Allied Sciences Senate Research and Publication Committee. This has been addressed in lines 228 to 230.

Comments from reviewer 1
1.I am not sure how UTI often leads to septicemia, dehydration. Sometimes from kidney infection bacteria may have chance to enter blood stream and cause bacteremia. Line #47-48 "Urinary tract infection (UTI) is common in under-five children presenting with fever with significant consequences leading to septicemia, dehydration, kidney scarring"

Response: The sentence has been revised in line 55 of the introduction section.

2. I suggest authors to describe the source and study population including Eligibility criteria for selection of the study participants. In this study it was mentioned that under five children with fever who visited the health care setting. Fever can indicate server condition (Acute febrile illness). Were there any additional criteria to select? What other laboratory tests were performed? Were infant included?
Response: We involved children aged 2 months and above who met the inclusion criteria. We have to revise and write: We recruited children with fever who the attending clinician suspected to have UTI based on signs and symptoms such as fever of 38oC or above, pain or burning when urinating, frequency urination, discoloured urine, lower abdominal pain, or vomiting.. The eligibility criteria have been added; line 106 to 111

3. Was there multiple infections?
Response: No multiple infection was obtained with significant growth of bacteria counts of 10^5 CFU/ml. We have rephrased the result section to reflect this finding line 275 to 276.

4. The description of AST in the manuscript may need revision example 'highly susceptible' as far as I know interpretation of AST result is Resistant, Intermediate or susceptible based on cut point. "E. coli were highly susceptible to cefepime"
Response: This has been addressed in table 4 of the manuscript, interpretation of AST which includes resistant, intermediate and sensitive were added in the table.

5. Was the tool (questionnaire) used in the study validate? Briefly describe
Response: Yes, the questionnaire used for data collection was validated, and this is explained in the data collection section, line 124.

6. It was indicated that the urine specimens were stored at 4oC until they tested. For

Powered by Editorial Manager® and ProduXion Manager® from Aries Systems Corporation

how long were they stored? How far regional lab is from the hospital where study participants were recruited. The integrity of the specimen is key for recovery of bacteria.
Response: Samples were stored in the refrigerator for not more than 12 hours; we have indicated this in the sample collection section line 141.

7. Describe how antibiotics used in the study were selected
Response: Descriptions on how antibiotics used in the study were selected has been added in line 174 to 175.

8. Did authors use only CLED for identification of Gram-positive and Gram-negative bacteria? How S. aureus and Micrococcus was differentiated? Overall, the types of culture media used for AST were not indicated.
Response: We differentiated Staphylococcus aureus from Micrococcus species by observing colony characteristics and performing a gram stain. The type of culture used for AST has been indicated in lines 175-177

9. As the definition of multi-drug resistance may vary it advisable to include operational definition for multi-drug resistance.
Response: We have added in the materials and methods a subsection of the definition of terms. The definition of multi-drug resistance has been included in lines 143-153

10. The sample size for was not indicted in the main method section.
Response: The sample has been indicated in methodology line 114

11. Consider including 95% CI for culture confirmed UTI.
Response: 95% CI has been added to the culture confirmed UTI in the abstract, results and discussion sections

12. Figure 1, Y-axis is not labelled. Also include frequency and % on the top of each bar
Response: Figure one was changed to a table 2 which indicates frequency and percentage

13. It is difficult to understand the results presented in Table 3. Consider including R, I, and S
Response: Table 3 has been revised for clarity and changed to table 4 since we added another table

Minor comments
1. Line# 135 “…..Identify…..’ the first letter should not be capital
2. ‘Gram-positive’ and ‘gram-positive’ is interchangeably used. It is good to stick to one. ‘Gram’ or ‘gram’
Response: The first letter of "identify" has been written in lowercase. "Gram positive" and "gram positive" have been revised to "gram positive." However, when starting a sentence, "Gram" is written with a capital letter

Comments from Review 2
Comments for Reviewer #2 were on the sticky note on the PDF file. We have addressed all comments and modified the manuscripts
In title you said etiological profile, is it include fungi?
Response: The title has been revised to reflect the bacterial etiology; line 1

Abstrct background:Where UTI is common infection?. Avoid use of short form in abstract. Better to be consistence as per title or should modify the title accordingly. Foristance febrile underfive,antibiotic vs antimicrobial,proportion of UTI vs etiology profile
Response: We rephrased the sentences for clarity and to ensure consistency, and short-form words were omitted; lines 17-23

Abstract methodology: Why only febrile? Is fever the only criteria to be suspected as UTI for under five? Operationalize this. The method is not written well, rewrite again
Response: The materials and methods subsection in the abstract has been rewritten for clarity; Lines 25-32

Powered by Editorial Manager® and ProduXion Manager® from Aries Systems Corporation

Abstract results : rewrite the result very well. Like of all 28.8% had Lab, confirmed means? do you mean culture confirmed? write the organism name in italic. Is circumcision a common practice for both male and female or for only for male? is those for female taken as nor not applicable? Rewrite bacteria in italic. Is suceptibility pattern for overall? or E.. coli & S. aurues
Response: The result subsection of the abstract has been rewritten for clarity. Bacteria have been written in italics; the susceptibility patterns are for E.coli and S. aureus; Lines 34-43

Introduction
Add One paragraph for common cause of UTI from different literature.
Response: A paragraph detailing the common causes of UTI has been included; Line 62-69

what does the renal functions of the kidney mean?
Response: The sentence has been revised to reflex that, Early diagnosis is essential to preserve and maintain the proper functioning of the kidneys and prevent any further decline in their function. Line 56-57

uncircumcision in male?
Response: Yes uncircumcission in male, The sentence has been paraphrased; Line 43&73

The prevalence of UTI among under-five children ranges from 7.4 % to 43.6% where this studies conducted
Response: The reported prevalence was indicated from Nigeria and Tanzania; Line 59-60

The risk factors for acquiring UTI in under-five children are multiple and differ with geographical locations, settings, and seasons. Add reference
Response: Reference number 9 has been added; Line 71

Antimicrobial resistance (AMR) has become significant concern worldwide.add reference
Response: Reference number 14 was added; Line 77

Can you generalize with your specific hospital data about the treatment? or can leads you to change treatment guidelines?
Response: The sentence has been ommited, since the same information were discussed in line 89-94

Materials and methods
Line 91, Can you clarify on primary health care and district hospital? in your title it says primary health care whereas the study is conducted in district hospital? Besides this your study area description don't indicate as whether it is urban or rural but in reasoning or gab analysis your said that rural community lacks attention?
Response: Yes, in Tanzania, a district hospital is considered a primary healthcare facility. The Magu district, where our research was conducted, is situated in the rural parts of the region. However, when classifying the demographic data, we categorized urban as individuals from the town area of Magu, and rural as those from other areas outside the town; Line 103

why only children with fever?
Response: Fever is a common symptom of various illnesses, the aim of recruiting children with fever ensured that,study participants have a higher likelihood of having the targeted condition; Line 106-109

The total sample size should be indicated here.
Response: The total sample size was indicated; Line 114

Powered by Editorial Manager® and ProduXion Manager® from Aries Systems Corporation

In Data collection your sociodemographic is limited why?
Response: The scope and objectives of this study prioritized these sociodemographic variables; Line 121

Is Sekou Toure Regional Referral Hospital different from above hospital?
Response: Yes, the study was conducted at Magu District Hospital. However, the samples were analyzed at Sekou Toure Regional Referral Hospital since the District hospital lacked the capacity to process culture samples

Line 131- 132, write more how do you handle in case of mixed growth
Response: Handling of mixed growth of bacteria has been indicated; Line 161-162

Line 135, Identify should start with small letter
Response: The word Identify written starting with small letter Line 165

is this the only biochemical test? how do you identify like P. aerigenosa & Acitenobacter spp
Response: Oxidase test used to identify P. aerigenosa & Acitenobacter spp has been added Line 168-169

Antimicrobial susceptibility testing: Can we report erthromycin for organisms from UTI? this shows you haven't followed the CLSI properly.
Response: Erthromycin disk has been removed from this manuscript

Extended-Spectrum Beta-Lactamase Detection: Is this for the three or for one? if for one you have write cefotaxime or cftriaxone or ceftazidime. is the increase of zone of inhibition towards CAZ or CXT or both?
Response: The sentence has been rephrased Line 192-193, 199-201

Quality assurance: As indicated in the quideline, on which guidelines?
Response: The sentence" as indicated in the quideline was ommited since checking expiration date and assess reagents does not need any guideline.

Data analysis: ≤ 0.2, this cut of value most of the time is 0.25. Do you have some justification for it?
Response: The choice of a cut-off value in data analysis can depend on the specific field of study, the nature of the data, and the research question being addressed, This cut-off value allows for broader inclusion of variables that may have some potential association with the outcome of interest. Line 223

Ethical approve: include ethical number
Response: Ethical clearance number MUHAS-REC-12-2022-1484 has been added Page 228

Results:
Socio-demographic and clinical characteristics: so you you justify as this study is conducted in rural area: No need of repeating 368
Response: The study was conducted in Magu District, which is primarily a rural area. However, during the recruitment of participants, some individuals residing in the town were included, categorizing them as urban participants. Additionally, participants from villages near Magu town were also included, classifying them as rural area participants. The sentence has been paraphrased. Line 241.

The proportion of UTI among under-five children: why you classify the age only in two category? do you have justification?
Response: Dividing age into two categories was made in order to compare the usage of nappies between two distinct age groups: children under 24 months and those above 24 months. Line 247-248

I think the use of p-value is not important here as the title is the proportion of UTI
Response: The p-value is used in proportion tests to assess the statistical significance of the observed sample proportion compared to a hypothesized population proportion. It helps determine whether the difference between the observed proportion and the hypothesized proportion is statistically significant or simply due to random chance Line

Powered by Editorial Manager® and ProduXion Manager® from Aries Systems Corporation

248

what does it means 0-2 days? is there a zero day?
Response: The sentence has been rewritten as ≤2 days; Line 251

is there a means that you know a child of five days have abdominal pain or not? similarly for others
Response: The doctor reported this after obtaining the child's clinical history from the parents or guardian and conducting a thorough physical examination; Line 244

Put p in italic.
Response: P value has been written in italic Line 247-260

is UTI only expected only at high temp what about lower temp?
Response: According to the World Health Organization (WHO), a body temperature of 38 degrees Celsius is generally considered a threshold for fever in both adults and children. Therefore, our studies specifically recruited children under the age of five with a fever of 38 degrees Celsius and above. Line 258-259

The line with overall is not correct. put it at the end of the table and the table header should have a line on the top and bottom. why only positive? remove p-value from here? is the p-value from chi-square of logistic regression?. 0.2 should be written 0-2. Axillary temperature of ≥39 should be written well. Place of residence, why it comes last? why not on socio-demographic? font style and size should be similar
Response: The line with overall proportion has been removed ,The line was added on top and bottom of table header, Negative n (%) column has been added in the table. P value used is from chi square and it tells as if the difference in proportion is significant or not significant. Duration of fever 0.2 was written well as <2; An axillary temperature of ≥39 was written well; The place of residence variable was shifted to demographic factors; Font size was setted Table 1

Modify the title “Bacteria causing UTI among under-five children”. you can staphylococcus aureus write in short form once you write in long and short form then after.
Response: The title has been modified to” Bacterial uropathogens isolated from Urinary Tract Infection positive under five children. Staphylococcus aureus has been written in short form.

Figure : think over febrile children. modify the organism name on the figure. remove the key from main figure and indicate each organism separately. Give title for x-and y-axis. Indicate number and % on bar graph.
Response: The figure was presented in Table including frequency and per cent of all isolates, Table 2

Factors associated with UTI: This sections seems more of discussion. try to write it properly again. what is CPR, and APR? is this common? I know COR & AOR? where candidate or associated? include OR,95%CI & P-value for each cells which is candidate.
Response: CPR =Crude Prevalence Ratio, APR=Adjusted Prevalence Ratio. These are used when the prevalence of the outcome is greater than 15. Our prevalence in this study was 28.8; hence, we used CPR and APR instead of COR&AOR. CPR and APR have been included. Lines 343 - 349

Table 2: Characteristics should be variables, Duration of fever, 0.2 should be 0-2, why uppercase for some variable? check for all. Male circumcision, how do handle for those female here?
Response: Characteristics have been changed to variable, Duration of fever 0.2 changed to ≤2, Upper case was changed to lower case in YES and NO. Females were excluded from the data as analysis was done. Table 3

write uniformly for all antibiotics you write in lower case for some and uppercase here
Response: Antibiotic was written in lowercase; Line 312

Tabel 3: consistence issue. when we think susceptibility we have to think about

Powered by Editorial Manager® and ProduXion Manager® from Aries Systems Corporation

Sensitive, Intermediate and Resistance both your table doesn't indicate this one. S.a not scientific similarly for the others. Key, this should be out of table and as keynote:
Response: The table title has been revised as sesntivity pattern for consistency. The pathogens names has been written in scientific form and the Key shifted out of the table; Table 4

MDR, ESBL and MRSA Should be defined in your method
Response: Defination of MDR, ESBL and MRSA has been added in the methods new susbsection of definition of terms line 143-153

Table 4, your title lucks consistency. I think MRSA and the like is should not be presented in one table. present with other way like pie chart. MDR, This should be presented separately & should be shown how it comes.
Response: MRSA and ESBL producers bacteria have been presented in a histogram in Figure 1 and MDR has been presented separately in Table 5

Discussion
I think it better you start with the strength and weakness of the study. rather than summarization of your finding. Your discussion poorly organized doesn't discus about the most important part of the study, ESBL, MRSA & MDR. Abserve consistency
Response: Discussion has been revised

Better show the number for comparison and put your study in CI. Similarly for the others include. Is this the only reason? what about nitrofurantoin?
Response: The number for comparisons has been added, and CI has been added in Line 343 to 349. Nitrofurantoin has been included in the discussion, Line 371 to 373.

“High resistance rates were observed to antibiotics commonly used in rural settings”, what does it mean? Provide reference
Response: The sentence has been rewritten as ‘The antibiotics frequently prescribed for empirical treatment Line 381

Do you have scientific background or reference for this? “ as this study was conducted in rural areas where UTI is more likely to be community-acquired, whereas, in urban areas, UTI is more likely to be acquired at the hospital from possibly admitted patients.
Response: The sentence has been removed as there is no proper reference to support its validity

put reference at the end of the sentence?
Response: The reference has been moved to the end of the sentence Line 384 and 400

Indicate which age group? Wearing of nappies, you assessed for all age?
Response: Wearing nappies werer assessed in children under 24 months; Line 402

you have to discuss circumcision separately and give emphasis even if your study subjects is both male and females. compare and contrast with other finding.
Response: Circumcision has been discussed in separate paragraph; Line 404-410

what does it mean? is this limitation of your study?if yes then write in the limitation section
Response: Limitation section has been added; Line 419-422

Conclusion and recommendation: Write as conclusion or separately. following the journal guideline. Not clear it is miss-leading; what does it mean? why only on AMR? looking at your title
Response: The section conclusion and recommmednation has been written as conclusion and content revised for clarity; Lines 425-432

Acknowledgements: we should start with capital letter. Who do you refer in “gratitude to respondents who willingly participated”?
We’ has been written starting with capital letter and the sentence has been rephrased

	for clarity; Lines 438-439<br><br>Reference: follow the journal guideline for referencing<br>References has been reviewed as per journal guideline; Line 460-577
**Additional Information:**	
**Question**	**Response**
**Financial Disclosure**<br><br>Enter a financial disclosure statement that describes the sources of funding for the work included in this submission. Review the submission guidelines for detailed requirements. View published research articles from *PLOS ONE* for specific examples.<br><br>This statement is required for submission and **will appear in the published article** if the submission is accepted. Please make sure it is accurate.<br><br>**Funded studies**<br>Enter a statement with the following details:<br>• Initials of the authors who received each award<br>• Grant numbers awarded to each author<br>• The full name of each funder<br>• URL of each funder website<br>• Did the sponsors or funders play any role in the study design, data collection and analysis, decision to publish, or preparation of the manuscript?<br><br>Did you receive funding for this work?	Yes
Please add funding details.<br>as follow-up to "**Financial Disclosure**<br><br>Enter a financial disclosure statement that describes the sources of funding for the work included in this submission. Review the submission guidelines for detailed requirements. View published research articles from *PLOS ONE* for specific examples.<br><br>This statement is required for submission and **will appear in the published article** if	This work is supported by the United States President’s Emergency Plan for AIDS Relief (PEPFAR) through the Centre of Excellence in Health Monitoring and Evaluation, Mzumbe University under the U.S Centers for Disease Control and Prevention (CDC), Project Cooperative Agreement No: NU2GGH002292 which supports Tanzania Field Epidemiology and Laboratory Training Program (TFELTP), however, the funder did not contribute to the study's design, data collection, analysis, interpretation, or manuscript writing

the submission is accepted. Please make sure it is accurate.<br><br>**Funded studies**<br>Enter a statement with the following details:<br>• Initials of the authors who received each award<br>• Grant numbers awarded to each author<br>• The full name of each funder<br>• URL of each funder website<br>• Did the sponsors or funders play any role in the study design, data collection and analysis, decision to publish, or preparation of the manuscript?<br><br>Did you receive funding for this work?"	
Please select the country of your main research funder (please select carefully as in some cases this is used in fee calculation).<br>as follow-up to "**Financial Disclosure**<br><br>Enter a financial disclosure statement that describes the sources of funding for the work included in this submission. Review the submission guidelines for detailed requirements. View published research articles from *PLOS ONE* for specific examples.<br><br>This statement is required for submission and **will appear in the published article** if the submission is accepted. Please make sure it is accurate.<br><br>**Funded studies**<br>Enter a statement with the following details:<br>• Initials of the authors who received each award<br>• Grant numbers awarded to each author<br>• The full name of each funder<br>• URL of each funder website<br>• Did the sponsors or funders play any role in the study design, data collection and analysis, decision to publish, or preparation of the manuscript?	UNITED STATES - US

Powered by Editorial Manager® and ProduXion Manager® from Aries Systems Corporation

Did you receive funding for this work?"	
**Competing Interests**<br><br>Use the instructions below to enter a competing interest statement for this submission. On behalf of all authors, disclose any competing interests that could be perceived to bias this work—acknowledging all financial support and any other relevant financial or non-financial competing interests.<br><br>This statement is required for submission and **will appear in the published article** if the submission is accepted. Please make sure it is accurate and that any funding sources listed in your Funding Information later in the submission form are also declared in your Financial Disclosure statement.<br><br>View published research articles from *PLOS ONE* for specific examples.<br><br>**NO authors have competing interests**<br><br>Enter: *The authors have declared that no competing interests exist.*<br><br>**Authors with competing interests**<br><br>Enter competing interest details beginning with this statement:<br><br>*I have read the journal's policy and the authors of this manuscript have the following competing interests: [insert competing interests here]*<br><br>* typeset	The authors of this research have explicitly stated that there are no existing conflicts of interest related to the publication of their work.
**Ethics Statement**<br><br>Enter an ethics statement for this submission. This statement is required if the study involved:<br><br>• Human participants<br>• Human specimens or tissue	The study obtained ethical clearance (MUHAS-REC-12-2022-1484) from the Muhimbili University of Health and Allied Sciences Research and Ethics Committee. In addition, we got permission for data collection from the offices of the Regional and District Medical Officers. Parents or guardians were informed about the study objectives and consented on behalf of their children before inclusion in the study. Results of urine culture and antimicrobial susceptibility tests were timely communicated to the attending clinician for patient management. Participant identification numbers were used in the study instead of names to ensure confidentiality.

- Vertebrate animals or cephalopods
- Vertebrate embryos or tissues
- Field research

Write "N/A" if the submission does not require an ethics statement.

General guidance is provided below. Consult the submission guidelines for detailed instructions. **Make sure that all information entered here is included in the Methods section of the manuscript.**

Powered by Editorial Manager® and ProduXion Manager® from Aries Systems Corporation

**Format for specific study types**<br><br>**Human Subject Research (involving human participants and/or tissue)**<br>• Give the name of the institutional review board or ethics committee that approved the study<br>• Include the approval number and/or a statement indicating approval of this research<br>• Indicate the form of consent obtained (written/oral) or the reason that consent was not obtained (e.g. the data were analyzed anonymously)<br><br>**Animal Research (involving vertebrate animals, embryos or tissues)**<br>• Provide the name of the Institutional Animal Care and Use Committee (IACUC) or other relevant ethics board that reviewed the study protocol, and indicate whether they approved this research or granted a formal waiver of ethical approval<br>• Include an approval number if one was obtained<br>• If the study involved *non-human primates*, add *additional details* about animal welfare and steps taken to ameliorate suffering<br>• If anesthesia, euthanasia, or any kind of animal sacrifice is part of the study, include briefly which substances and/or methods were applied<br><br>**Field Research**<br><br>Include the following details if this study involves the collection of plant, animal, or other materials from a natural setting:<br>• Field permit number<br>• Name of the institution or relevant body that granted permission	
**Data Availability**<br><br>Authors are required to make all data underlying the findings described fully available, without restriction, and from the time of publication. PLOS allows rare exceptions to address legal and ethical concerns. See the PLOS Data Policy and FAQ for detailed information.	Yes - all data are fully available without restriction

A Data Availability Statement describing where the data can be found is required at submission. Your answers to this question constitute the Data Availability Statement and **will be published in the article**, if accepted.<br><br>**Important:** Stating 'data available on request from the author' is not sufficient. If your data are only available upon request, select 'No' for the first question and explain your exceptional situation in the text box.<br><br>Do the authors confirm that all data underlying the findings described in their manuscript are fully available without restriction?	
**Describe where the data may be found in full sentences. If you are copying our sample text, replace any instances of XXX with the appropriate details.**<br><br>• If the data are **held or will be held in a public repository**, include URLs, accession numbers or DOIs. If this information will only be available after acceptance, indicate this by ticking the box below. For example: *All XXX files are available from the XXX database (accession number(s) XXX, XXX.*).<br>• If the data are all contained **within the manuscript and/or Supporting Information files**, enter the following: *All relevant data are within the manuscript and its Supporting Information files.*<br>• If neither of these applies but you are able to provide **details of access elsewhere**, with or without limitations, please do so. For example:<br><br>*Data cannot be shared publicly because of [XXX]. Data are available from the XXX Institutional Data Access / Ethics Committee (contact via XXX) for researchers who meet the criteria for access to confidential data.*<br><br>*The data underlying the results presented in the study are available from (include the name of the third party*	The datasets used in this study are publicly available and can be acquired upon request from the corresponding author.

and contact information or URL).<br>• This text is appropriate if the data are owned by a third party and authors do not have permission to share the data.<br><br>* typeset	
Additional data availability information:	

Powered by Editorial Manager® and ProduXion Manager® from Aries Systems Corporation

Bacterial aetiology, Antimicrobial Susceptibility Patterns, and Factors Associated with Urinary Tract Infection among Under-five Children at Primary Health Facility, North-Western Tanzania

Roza Ernest[1,2*], Nsiande Lema[1], Sued Yassin[3], Agricola Joachim[4], Mtebe Majigo[4].

[1] Tanzania Field Epidemiology and Laboratory Training Program, Dar es Salaam, Tanzania
[2] Department of Epidemiology and Biostatistics, Muhimbili University of Health and Allied Sciences, Dar es Salaam, Tanzania
[3] Research, Training, and Consultancy Unit, Chato Zonal Referral Hospital, Geita, Tanzania
[4]Department of Microbiology and Immunology, Muhimbili University of Health and Allied Sciences, Dar es Salaam, Tanzania

***Email:** roza.ernest@yahoo.com (RE)

Abstracts

Background: Urinary tract infection is common in under-five children presenting with fever with significant consequences leading to bacteremia, dehydration, kidney scarring, and renal failure. The incidence of Urinary tract infection varies with age, gender, health condition, and geographic location. There is a lack of studies addressing these issues, particularly concerning children. This study determined the proportion of urinary tract infections, bacterial aetiology, antimicrobial susceptibility patterns, and factors associated with urinary tract infection among under-five children.

Materials and methods: The cross-sectional analytical study was conducted at the District Hospital in Mwanza Region, North-Western Tanzania, between March and April 2023. A convenient non-probability sampling technique was used to collect urine samples from children under five years old with signs and symptoms of urinary tract infection. Written informed consent was obtained from all parents or guardians. Participants' information was collected using a pretested structured questionnaire. Urine samples were processed at Sekou Toure Regional Referral Hospital. STATA version 15.0 was used for data analysis. Factors associated with Urinary tract infection were determined using a Modified Poisson Model.

Result: The study recruited 368 under-five children; 194 (52.7%) were males. The median age (interquartile range) was 24 (13-36) months. Of all, 28.8% (95% CI-24.3-33.6) had urinary tract infections. One hundred and six pathogens were isolated, the majority being *Escherichia coli*, 37 (34.9%), and *Staphylococcus aureus*, 26 (24.5%). *Escherichia coli* were susceptible to cefepime, piperacillin-tazobactam, nitrofurantoin, and meropenem, ranging from 81.1% to 97.3%. *Staphylococcus aureus* was susceptible to nitrofurantoin (96.2%) and ciprofloxacin (92.3%). Multidrug resistance was observed in 33.0% of isolates, Methicillin-Resistance *Staphylococcus aureus* in 23.1% of *Staphylococcus aureus*, and extended-spectrum beta-lactamases in 25% of gram-negative bacteria. Urinary tract infection was associated with vomiting, age, dysuria, abdominal pain, nappy use, and uncircumcision in males.

Conclusion: Our study found a relatively high proportion of urinary tract infections among under-five children. *Escherichia coli* and *Staphylococcus aureus* were the common isolated organisms susceptible to nitrofurantoin, ciprofloxacin, meropenem, cefepime, and piperacillin-tazobactam, and least susceptible to commonly used antibiotics. Empirical treatment of urinary tract infection should be reviewed periodically based on the antibiotic susceptibility pattern.

Keywords: Urinary Tract Infection, Antimicrobial susceptibility pattern, under-fives children

Introduction

Urinary tract infection (UTI) is common in under-five children presenting with fever with significant consequences leading to bacteremia, dehydration, kidney scarring, and renal failure (1). Early diagnosis is essential to preserve and maintain the proper functioning of the kidneys and prevent any further decline in their function (2,3). After acute respiratory infections, UTI has been reported as the second most typical site of infection in the community, and incidence varies with age, gender, and health condition. Studies conducted in Nigeria and Tanzania have reported the prevalence of UTI in under-five children ranging from 7.4% to 43.6% (1,4,5).

UTI in children is primarily caused by bacteria, but other pathogens such as viruses, fungi, and parasites can also be responsible. The most common bacteria associated with UTI in children include gram-negative pathogens such as *Escherichia coli, Klebsiella, Proteus, Enterobacter, Pseudomonas,* and *Serratia.* Gram-positive pathogens associated with UTI in children include

group B *streptococcus, enterococcus* spp, and *Staphylococcus aureus*. In addition to bacterial causes, non-bacterial causes include viruses causing viral haemorrhagic cystitis and Candida infection, particularly in individuals with compromised immune systems (4,6,7). The bacteria causing UTI have varied antibiotic susceptibility patterns, most resistant to commonly used antibiotics (4,8).

The risk factors for acquiring UTI in under-five children are multiple and differ with geographical locations, settings, and seasons (9). The commonly reported factors include female gender, poor hygiene, poverty, malnutrition, and recurrent infections (10,11). Other predisposing factors include age, uncircumcision in males (12), genitourinary anomalies, indwelling urinary catheters, and previous antibiotic use (13).

Urine culture is rarely performed as part of patient management in rural areas with inadequate diagnostic resources. Treatment is empirical, which could encourage the development and spread of antimicrobial-resistant bacteria (14). Antimicrobial resistance (AMR) has become a significant concern worldwide (14). Recent research has shown a high prevalence of resistance to commonly used antibiotics (14–16). Antimicrobial resistance is also prevalent in Tanzania, with reports indicating multi-drug resistance among urinary pathogens associated with UTIs (17).

Extended-spectrum beta-lactamases (ESBL) producers contribute a high resistance rate to all beta-lactam antibiotics and are more common in hospitals than in the community or outpatient settings (17,18). ESBL-producing bacteria prevalence of infection or colonisation among children under five years ranges from 17% to 35% (18–21). Studies in Tanzania reported that ESBL producers' prevalence ranges from 18% to 45%; Carbapenems were reported as the drugs to treat ESBL-producing bacteria (17,22). However, they are expensive and not widely available in Tanzania.

Therefore, increasing knowledge of the aetiologic profile and antimicrobial susceptibility patterns of pathogens associated with UTI in under-fives is essential in medical practices. Understanding the associated factors also helps identify at-risk groups and institute preventive measures. This research paper explored the etiologic profile, antimicrobial susceptibility pattern, and factors associated with UTI among under-five children in Magu District Hospital, Mwanza-Tanzania.

Materials and Methods

Study design, setting, and population

We conducted a cross-sectional analytical study design between 8th March and 14th April 2023 at Magu District Hospital in Mwanza Region, North-Western Tanzania. Magu district is located between 33° and 34° East of Greenwich and between 2°10′ and 2°50′ South of the Equator. Its total area is 3075 km2, of which Lake Victoria waters cover 1725 km2 (56.1%). The average annual rainfall is between 700 and 1000 mm. As of 2012, the population of Magu district was 299,759 people. Magu District Hospital is a referral hospital in the district. Over 40% of the patient population seeking services is under-fives children. The laboratory procedures were conducted at Sekou Toure Regional Referral Hospital Laboratory.

The study involved under-five children with a fever seeking healthcare services. We recruited children with fever who the attending clinician suspected to have UTI based on signs and symptoms such as fever of 38 degrees Celsius or above, pain or burning when urinating, frequent urination, discoloured urine, lower abdominal pain, or vomiting. The study did not include children who used catheters within 72 hours because catheter insertion may directly inoculate microorganisms into the urinary bladder.

Sample Size and Sampling Technique

We estimated the minimum sample size of 355 under five children using the Kish Leslie formula considering the 11.4% previously reported prevalence of UTI among under-five children (23). A non-probability convenient sampling technique was used where all febrile under-five children attending Magu District Hospital were recruited in the study after obtaining consent from the parent/guardian.

Data collection

The participants' socio-demographic (age, sex, and place of residence), clinical characteristics (vomiting, dysuria, body temperature, abdominal pain, and chronic disease), and risk factors (nutrition status, history of recurrent UTI, male circumcision, duration of fever, and nappy use) were collected using a structured and pre-tested questionnaire. Axillary temperature was measured using a digital thermometer. The children's body weight was measured using a digital weighing scale. The nutrition status of the children was determined by calculating their weight-for-age z-scores (WAZ), which represent how many standard deviations their weight is from the median weight of a healthy reference population of the same age and sex. To assess their

nutritional status, a WAZ below -3 standard deviations was used to indicate malnutrition, while a WAZ between -2 and +2 standard deviations was used to indicate a normal nutritional status (well-nourished) (24).

Sample collection

A urine specimen for each participant was collected. After receiving adequate instruction, the study participant's parents or guardians assisted with the urine collection. Approximately 10ml of urine specimens were collected into a sterile, wide-mouth, leak-proof, screw-capped plastic container pre-labelled with the participant's identification number, age, date, and time. The specimen was transported daily to Sekou Toure Regional Referral Hospital laboratory for processing within two hours and kept in a cool box. Immediate inoculation of samples was done on arrival at the laboratory. If a delay was inevitable, specimens were refrigerated at 4°C not more than 12 hours.

Definition of terms

For the purpose of the current study, multidrug resistance (MDR) was defined as resistance to at least one antimicrobial agent in three or more antimicrobial categories. Methicillin-resistant *Staphylococcus aureus* was defined as an isolate of staphylococcus aureus with an inhibition zone of less than or equal to 21mm diameter around the 30 µg cefoxitin disk on antimicrobial susceptibility disk diffusion method, and Extended-spectrum beta-lactamases producers was defined as gram negative bacteria that shows an increase in the inhibition zone towards the amoxicillin-clavulanic acid disk in either the cefotaxime disk or ceftazidime disk after placing. Ceftazidime (30µg), cefotaxime (30µg), and amoxicillin-clavulanic acid (20/10 µg) disks in a straight line 20 mm apart on the Muller Hinton plate, with amoxicillin-clavulanic acid in the center.

Urine Culture

We performed a quantitative urine culture to determine the significant bacteriuria. Urine samples were inoculated into Cysteine Lactose-Electrolyte Deficient (CLED) (Sigma Alorich, India) agar plates using a calibrated wire loop (0.001mL). Culture plates were incubated under aerobic conditions at 37°C for 24 hours. Bacterial growth of $\geq 10^5$ colony-forming unit (CFU)/mL of urine was considered significant bacteriuria. Specimens yielding fewer than 10^5

CFU/mL were considered insignificant or potentially contaminated. In instances of mixed bacterial growth, subculturing was conducted to isolate each organism separately.

Bacteria identification

We used conventional methods to identify isolated bacteria, including colony characteristics, gram staining reaction, and biochemical testing. Sulphur Indole Motility (SIM) (Himedia, India), Kligler Iron Agar test (Oxoid Ltd, UK), urease test (Oxoid Ltd, UK), and citrate utilisation test (Oxoid Ltd, UK) were used to identify gram-negative bacteria. Oxidase test (Himedia, India) was used to identify *pseudomonas* and *Acinetobacter*. Catalase and coagulase tests were used to identify gram-positive bacteria.

Antimicrobial susceptibility testing

Antimicrobial susceptibility testing was performed using the Kirby-Bauer disk diffusion method adhering to Clinical and Laboratory Standards Institute (CLSI) guidelines (25). The selection of antibacterial disks was based on CLSI 2022 recommendations and availability. The bacterial inoculum, equivalent to 0.5 McFarland standards, was uniformly spread onto Mueller–Hinton agar plates (Himedia India) using a sterile cotton swab applicator. Antibiotic disks for gram-positive bacteria were penicillin G (10 μg), nitrofurantoin (300 μg), ciprofloxacin (5μg), trimethoprim-sulfamethoxazole (1.25/23.75 μg), gentamicin (10 μg), cefoxitin (30 μg), and clindamycin (30 μg) (Himedia, India).

The antimicrobial disks for Enterobacteriaceae included piperacillin-tazobactam (100/10 μg), ampicillin (10 μg), nitrofurantoin (300 μg), amoxicillin-clavulanate (20/10 μg), gentamicin (10 μg), cefotaxime (30 μg), ciprofloxacin (30 μg), meropenem (10 μg), piperacillin (100 μg), cefepime (30 μg), amikacin (30 μg), trimethoprim-sulfamethoxazole (1.25/23.75 μg), ceftazidime (30 μg), and ceftriaxone (30 μg) (Himedia, India). Antimicrobial disks for *Pseudomonas* spp and *Acinetobacter* were gentamicin (10 μg), meropenem (10 μg), ceftazidime (30 μg), piperacillin-tazobactam (100/10 μg), ciprofloxacin (5 μg), and amikacin (30 μg) (Himedia, India).

Extended-Spectrum Beta-Lactamase Detection

Gram-negative bacteria producing ESBL was first suspected from the result of antimicrobial susceptibility testing based on the diameter of the zone of inhibition for the three third-generation cephalosporins: cefotaxime (30μg), ceftriaxone (30μg) and ceftazidime (30μg). The

following breakpoints were used to suspect ESBL production: ceftriaxone ≤ 25mm, ceftazidime ≤ 22mm, and cefotaxime ≤ 27mm.

We confirmed ESBL production using the Double Disk Synergy method (26). Ceftazidime (30µg), cefotaxime (30µg), and amoxicillin-clavulanic acid (20/10 µg) disks (Himedia, India) were placed in a straight line 20 mm apart on the plate, with amoxicillin-clavulanic acid in the center. An increase in the inhibition zone towards the amoxicillin-clavulanic acid disk in either the cefotaxime disk or ceftazidime disk was a positive result for ESBL enzyme production; the isolate was reported as an ESBL producer.

Detection of methicillin-resistant *Staphylococcus aureus*

Methicillin-resistant *Staphylococcus aureus* (MRSA) was phenotypically determined using a cefoxitin (30 µg) disk (Oxoid Ltd, UK) per CLSI guidelines (25). A cefoxitin disk was applied and incubated at 37^0C for 18 to 24 hours. A less or equal to 21 mm inhibition zone diameter around the cefoxitin disk indicated MRSA.

Quality assurance

Media and reagents were checked by verifying the expiration date, storage conditions, and visual inspection of any signs of contamination, such as discolouration, turbidity, or particulate matter. The media quality was assessed using sterility and performance tests using reference strains. Reference strains for culture and susceptibility testing included *P. aeruginosa* (ATCC 27853), *S. aureus* (ATCC 25923), and *E. coli* (ATCC 25922). Additionally, *E. coli* (ATCC 25922) and *K. pneumonia* (ATCC 700603) ESBL-positive strains were used to control ESBL production. *S. aureus* (ATCC 43300) was used as a positive control for MRSA.

Data analysis

Data analysis was done using STATA version 15 StataCorp.2017. Frequency and proportions were used to summarise the characteristics of study populations for categorical variables, whereas median and interquartile range were used to summarise continuous variables. Pearson's chi-square test was used to compare a categorical variable to the status of UTI. The modified Poisson model was performed to determine factors associated with UTI. The multivariable analysis involved variables with a p-value ≤ 0.2 to determine the adjusted prevalence ratio and 95% confidence intervals. A p-value < 0.05 was used in both models to acknowledge statistical significance.

Ethical approval

The study obtained ethical clearance number MUHAS-REC-12-2022-1484 from the Muhimbili University of Health and Allied Sciences Senate Research Committee and Publications Committee. In addition, we got permission for data collection from the offices of the Regional and District Medical Officers. Parents or guardians were informed about the study objectives and consented on behalf of their children before inclusion in the study. Results of urine culture and antimicrobial susceptibility tests were timely communicated to the attending clinician for patient management. Participant identification numbers were used in the study instead of names to ensure confidentiality.

Results

Socio-demographic and clinical characteristics

A total of 368 under-five children were enrolled in the study. Males counted for more than half of the participants, 194 (52.7%). The study participants' median age (interquartile range) was 24 (13-36) months. Most participants, 296 (80.4%), lived in rural areas. Malnutrition was observed in 27 (7.3%) and 29 (14.9%) males were already circumcised. Most participants, 201 (54.6%), had a fever for 3 to 7 days, and the majority, 348 (94.6%), had a 38-39 ^{0}C temperature. Abdominal pain was observed in 153 (41.6%), while 61 (16.6%) had dysuria. Vomiting was observed in 84 (22.8%) participants, and 24 (6.5%) had a history of recurrent UTI (Table 1).

The proportion of UTI among under-five children

UTI was detected in 28.8% (106/368, 95% CI -24.3-33.6), more in children below 24 months, 53 (35.1%), than in children aged 24 months and above, 53 (24.4%) (p = 0.026). UTI was significantly higher in children with dysuria, 30 (49.2%) compared to those without dysuria, 76 (24.8%) (p < 0.001). The proportion of UTI was higher among children with a fever of 3-7 days 68 (33.8%) compared to those with a fever of ≤ 2 days 33 (21.3%) and greater than 7 days 5 (41.7%) (p = 0.021). Children presented with abdominal pain were more diagnosed with UTI, 81 (52.9%), than those without abdominal pain, 25 (11.6%) (p < 0.001). The proportion of UTI was found to be higher among children who used nappies, 56 (34.8%), than in children who did not use nappies, 50 (24.2%) (p=0.026). Uncircumcised male children had more UTI, 55 (33.3%) than circumcised children, 3 (10.3%) (p = 0.030). The proportion of UTI was significantly higher in children presented with vomiting 37 (44.0%) than in those without vomiting 69 (24.3%) (p < 0.001). The proportion of UTI was higher in children with a body

temperature above 39°c, 11 (55.0%) than in children with a body temperature between 38-39 ^{O}C, 95 (27.3%) (*p*=0.008). There was no difference in UTI proportion based on sex, history of recurrent UTI, place of residence, nutrition status, or chronic disease, and the p-value was greater than 0.05 (Table 1).

Table 1: Proportion of UTI among the febrile under-five children attending Magu District Hospital in Mwanza region, March to April 2023

Variable	Description	Frequency N (%) *	UTI negative n (%)**	UTI positive n (%) ***	P value
**Age group (months)**	<24	151(41.0)	98 (64.9)	53 (35.1)	0.026
	≥24	217(59.0)	164 (75.6)	53 (24.4)	
**Sex**	Male	194 (52.7)	136 (70.1)	58 (29.9)	0.625
	Female	174 (47.3)	126 (72.4)	48 (27.6)	
**Place of residence**	Urban	72 (19.6)	52 (72.6)	20 (27.8)	0.830
	Rural	296 (80.4)	210 (70.9)	86 (29.1)	
**Duration of fever (days)**	≤2	155 (42.1)	122 (78.8)	33 (21.3)	0.021
	3-7	201 (54.6)	133 (66.2)	68 (33.8)	
	>7	12 (3.3)	7 (58.3)	5 (41.7)	
**Axillary Temperature**	38-39	348 (94.6)	253 (72.7)	95 (27.3)	0.008
	>39	20 (5.4)	9 (45)	11 (55.0)	
**Dysuria**	Yes	61 (16.6)	31 (50.8)	30 (49.2)	< 0.001
	No	307 (83.4)	231 (75.2)	76 (24.8)	
**Vomiting**	Yes	84 (22.8)	47 (56)	37 (44.0)	< 0.001
	No	284 (77.2)	215 (75.7)	69 (24.3)	
**Abdominal pain**	Yes	153 (41.6)	72 (47.1)	81 (52.9)	< 0.001
	No	215 (58.4)	190 (88.4)	25 (11.6)	
**Recurrent UTI**	Yes	24 (6.6)	17 (70.8)	7 (29.2)	0.968
	No	344 (93.5)	245 (71.2)	99 (28.8)	
**Use of nappy**	Yes	161 (43.8)	105 (65.2)	56 (34.8)	0.026
	No	207 (56.2)	157 (75.8)	50 (24.2)	
**Chronic disease**	Yes	8 (2.2)	6 (75)	2 (25.0)	0.810
	No	360 (97.8)	256 (71.1)	104 (28.9)	
**Nutrition status**	Malnourished	27 (7.3)	17 (63)	10 (37.0)	0.326
	Well-nourished	341 (92.7)	245 (71.8)	96 (28.2)	
**Male circumcision (n = 194)**	Yes	29 (14.9)	26 (89.7)	3 (10.3)	0.030
	No	165 (85.1)	110 (66.7)	55 (33.3)	

*UTI=Urinary Tract Infection, *Frequency and percentage of participants calculated as number within the variable category divided by the total sample size of the variable,*

*** proportion of patients within the variable category with no Urinary Tract Infection calculated as number of divided by the total number of individuals in the variable category*

**** proportion of patients within the variable category with Urinary Tract Infection calculated as number of cases divided by the total number of individuals in the variable category*

Uropathogens bacteria causing UTI

A total of 106 pathogenic bacteria strains were isolated from 106 urine specimens of febrile under-five children. No multiple infection was obtained with significant growth of bacteria counts of ≥10^5 CFU/ml. Gram-negative bacteria constituted 80 (75.48%), while gram-positive bacteria counted 26 (24.52%). *E. coli* was the most common isolated pathogen, 37 (35%), followed by *S.aureus* 26/106 (25%) (Table 2).

Table 2: Frequency of bacteria isolates among children under five years attending Magu District Hospital in Mwanza region, March to April 2023

Isolated bacteria	Frequency	Percentage
Escherichia coli	37	34.9
Staphylococcus aureus	26	24.5
Enterobacter spp	10	9.4
Klebsiella spp	12	11.3
Acinetobacter spp	5	4.7
Morganella morganii	5	4.7
Proteus spp	4	3.8
Pseudomonas aureginosa	4	3.8
Providencia spp	2	1.9
Citrobacter spp	1	0.9

Factors Associated with Urinary Tract Infection

In univariate analysis, age, vomiting, dysuria, body temperature, abdominal pain, use of nappy, and uncircumcision were associated with UTI. Children below 24 months (CPR 1.4; 95% CI: 1.0–1.9, $P = 0.03$), who used nappies (CPR 1.4; 95% CI: 1.0–1.9, $P = 0.026$), and those presented with vomiting (CPR 1.8; 95% CI: 1.3–2.5, $P < 0.001$), dysuria (CPR 1.9; 95% CI: 1.4–2.7, $P < 0.001$), and body temperature above 39°C (CPR 2.0; 95% CI: 1.4–2.7, $P = 0.02$) were more likely to have UTI than their counterparts, with the prevalence ranging between 1.4 and 2.0, $p < 0.05$. Children with abdominal pain had 4.6 times more UTI than those without (CPR 4.6; 95%CI:3.1-6.8, $p < 0.001$). Uncircumcised children had 3.2 times more chances of acquiring UTI than circumcised (CPR 3.2; 95% CI: 1.1–9.6, $P = 0.035$). Sex, duration of fever, history of recurrent UTI, place of residence, nutrition status, and a history of having chronic disease were not associated with UTI.

Eight factors were included in the multivariable analysis. Age below 24 months, abdominal pain, vomiting, dysuria, body temperature above 39°C, use of nappy, and uncircumcision were independently associated with UTI. Children below 24 months had 1.5 times the prevalence of

acquiring UTI compared to those aged 24 months and above (APR= 1.5, 95% CI = 1.1–2.1, *P* = 0.010). Furthermore, children with abdominal pain had 3.9 times the prevalence of having a UTI than those without abdominal pain (APR = 3.9, 95% CI = 2.6–5.9, *P* < 0.001). Having vomiting, dysuria, or a body temperature above 39°C increased the prevalence of having UTI by 1.6 to 1.7 times. Using a nappy increased the prevalence of acquiring UTI by 1.2 times more than not using a nappy (APR 1.2; 95% CI: 0.6–2.4, *P* = 0.003). Uncircumcised children had 2.5 times the prevalence of acquiring UTI than circumcised (APR 2.5; 95% CI: 0.4–8.2, *P* = 0.002) (Table 3).

Table 3: Factors associated with UTI among febrile children under five years in Magu District -Mwanza region, March to April 2023

Variable	UTI negative n (%)	UTI positive n (%)	Bivariate analysis		Multivariate analysis	
			CPR (95% CI)	*P* value	APR (95%CI)	*P* value
**Age group (months)**						
<24	98 (64.9)	53 (35.1)	1.4 (1.0-1.9)	0.030	1.5 (1.1-2.1)	0.010
≥24	164(75.6)	53 (24.4)	1			
**Sex**						
Male	136 (70.1)	58 (29.9)	1.0 (0.8-1.5)	0.625		
Female	126 (72.4)	48 (27.6)	1			
**Place of residence**						
Urban	72 (72.2)	20 (27.8)	1			
Rural	296 (78.9)	86 (29.1)	1.0 (0.7-1.6)	0.831		
**Duration of fever (days)**						
≤2	122 (78.8)	33 (21.3)	1			
3-7	133 (66.2)	68 (33.8)	1.5 (1.1-2.1)	0.020	1.4 (0.9-1.9)	0.057
>7	7 (58.3)	5 (41.7)	1.5 (0.7-2.9)	0.275	1.5 (0.7-2.9)	0.179
**Temperature**						
38-39	253 (72.7)	95 (27.3)	1			
>39	9 (45.0)	11 (55.0)	2.0 (1.3-3.1)	0.002	1.7 (1.1-2.8)	0.020
**Dysuria**						
Yes	31 (50.8)	30 (49.2)	1.9 (1.4-2.7)	< 0.001	1.6 (1.2-2.3)	0.010
No	231 (75.2)	76 (24.8)	1			
**Vomiting**						
Yes	47 (56.0)	37 (44.0)	1.8 (1.3-2.5)	< 0.001	1.6 (1.2-2.2)	0.002
No	215 (75.7)	69 (24.3)	1			
**Abdominal pain**						
Yes	72 (47.1)	81 (52.9)	4.6 (3.1-6.8)	< 0.001	3.9 (2.6-5.9)	< 0.001
No	190 (88.4)	25 (11.6)	1			
**Recurrent UTI**						
Yes	17 (70.8)	7 (29.2)	1.0 (0.5-1.9)	0.968		
No	245 (71.2)	99 (28.8)	1			
**Use of nappy**						
Yes	105 (65.2)	56 (34.8)	1.4 (1.0-1.9)	0.026	1.2 (0.6-2.4)	0.003
No	157 (75.8)	50 (24.2)	1			
**Chronic disease**						
Yes	6 (75)	2 (25.0)	0.9 (0.3-2.9)	0.815		

No	256 (71.1)	104 (28.9)	1			
**Nutrition status**						
Malnourished	17 (63)	10 (37.0)	1.3 (0.8-2.2)	0.302		
Well-nourished	245 (71.8)	96 (28.2)	1			
**Male circumcision** (n = 194)						
Yes	26 (89.7)	3 (10.3)	1			
No	110 (66.7)	55 (33.3)	3.2 (1.1-9.6)	0.035	2.5 (0.4-8.2)	0.002

UTI: urinary tract infection, CPR: Crude Prevalence Ratio, APR: Adjusted Prevalence Ratio

Antimicrobial susceptibility patterns of isolated bacteria

Escherichia coli were susceptible to cefepime, piperacillin-tazobactam, nitrofurantoin, and meropenem, ranging from 81.1% to 97.3%, while least susceptible to trimethoprim-sulfamethoxazole (37.8%), gentamycin (32.4%), and ampicillin (18.9%). *Staphylococcus aureus* was susceptible to nitrofurantoin (96.2%) and ciprofloxacin (92.3%), and least susceptible to penicillin (11.5%). Susceptibility to ciprofloxacin ranges from 40% (*Acinetobacter* spp) to 100% *Pseudomonas aeruginosa, Citrobacter* spp, *Morganella morganii, Proteus* spp, and *Providencia* spp. Susceptibility to meropenem ranges from 20% *Acinetobacter* to 100% *Enterobacter* species. Trimethoprim-sulfamethoxazole susceptibility ranges from (37.8% *E. coli)* to 70% (*S. aureus)* (Table 4).

Table 4: Antimicrobial Susceptibility patterns of bacteria causing UTI among children under five years attending Magu District Hospital in Mwanza region, March to April 2023.

**Bacteria isolates**	**N**	**Pattern**	**NIT**	**CIP**	**SXT**	**GEN**	**AMP**	**AMC**	**CTX**	**CRO**	**CAZ**	**MRP**	**FEF**	**TZP**	**AK**	**CL**
		S	32(86.5)	28(75.7)	14(37.8)	12(32.4)	7(18.9)	25(67.6)	25(67.5)	29(78.4)	27(72.9)	36(97.3)	30(81.1)	32(86.5)	28(75.7)	NA
	37	I	2(5.4)	5(13.5)	8(21.6)	1(2.7)	13(35.1)	6(16.2)	8(21.6)	4(10.8)	4(10.8)	0(0)	4(10.8)	4(10.8)	6(16.2)	NA
E. coli		R	3(8.1)	4(10.8)	15(40.5)	24(64.9)	17(45.9)	6(16.2)	4(10.8)	4(10.8)	6(16.2)	1(2.7)	3(8.1)	1(2.7)	3(8.1)	NA
Klebsiella spp	12	S	11(91.7)	10(83.3)	8(66.7)	3(25.0)	(0.0)	7(58.3)	6(50.0)	7(58.4)	8(66.7)	11(91.7)	10(83.3)	10(83.3)	8(66.7)	NA
		I	1(8.3)	1(8.3)	0(0)	3(25.0)	2(16.7)	3(25.0)	3(25.0)	1(8.3)	0(0)	0(0)	1(8.3)	1(8.3)	0(0)	NA
		R	0(0)	1(8.3)	4(33.3)	6(50.0)	10(83.3)	2(16.7)	3(25.0)	4(33.3)	4(33.3)	1(8.3)	0(0)	1(8.3)	4(33.3)	NA
Enterobacter spp	10	S	8(80.0)	9(90.0)	6(60.0)	3(30.0)	3(30.0)	10(90.0)	6(60.0)	7(70.0)	9(90.0)	10(100.0)	8(80.0)	9(90.0)	9(90.0)	NA
		I	1(10.0)	1(10.0)	1(10.0)	0()	1(10.0)	1(10.0)	4(40.0)	2(20.0)	0(0)	0(0)	2(20.0)	0(0)	0(0)	NA
		R	1(10.0)	0()	3(30.0)	7(70.0)	6(60.0)	0(0)	0(0)	1(10.0)	1(10.0)	0(0)	0(0)	1(10.0)	1(10.0)	NA
Pseudomonas aeruginosa	4	S	NA	4(100.0)	NA	0(0.0)	NA	NA	NA	NA	1(25.0)	4(100.0)	4(100.0)	4(100.0)	4(100.0)	NA
		I	NA	0(0.0)	NA	0(0.0)	NA	NA	NA	NA	2(50.0)	0(0.0)	0(0.0)	0(0.0)	0(0.0)	NA
		R	NA	0(0.0)	NA	4(100.0)	NA	NA	NA	NA	1(25.0)	0(0.0)	0(0.0)	0(0.0)	0(0.0)	NA
Acinetobacter spp	5	S	NA	5(100.0)	NA	1(20.0)	NA	NA	NA	NA	1(20.0)	5(100.0)	5(100.0)	4(80.0)	4(80.0)	NA
		I	NA	0(0)	NA	0(0)	NA	NA	NA	NA	1(20.0)	0(0)	0(0)	1(20.0)	0(0)	NA
		R	NA	0(0)	NA	4(80.0)	NA	NA	NA	NA	3(60.0)	0(0)	0(0)	0(0)	1(20.0)	NA
Morganella spp	5	S	3(60.0)	5(100)	4(80.0)	2(40.0)	1(20.0)	3(60.0)	3(60.0)	3(60.0)	4(80.0)	4(80.0)	5(100.0)	5(100.0)	5(100.0)	NA
		I	1(20)	0(0)	0(0)	0(0)	0(0)	1(20)	1(20)	0(0)	0(0)	1(20)	0(0)	0(0)	0(0)	NA
		R	1(20)	0(0)	1(20)	3(60)	4(80)	1(20)	1(20)	2(40.0)	1(20)	0(0)	0(0)	0(0)	0(0)	NA
Proteus spp	4	S	3(75.0)	4(100.0)	3(75.0)	1(25.0)	0.0	3(75.0)	2(50.0)	3(75.0)	4(100.0)	3(75.0)	4(100.0)	4(100.0)	3(75.0)	NA
		I	0(0)	0(0)	0(0)	0(0)	0(0)	0(0)	1(25.0)	0(0)	0(0)	0(0)	0(0)	0(0)	0(0)	NA
		R	1(25.0)	0(0)	1(25.0)	3(75.0)	4(100)	1(25.0)	1(25.0)	1(25.0)	0(0)	1(25.0)	0(0)	0(0)	1(25.0)	NA
Other bacteria spp	3	S	3(100.0)	3(100.0)	0(0)	1(33.3)	0(0.0)	2(66.7)	(66.7)	(66.7)	(100.0)	(100.0)	(100.0)	(100.O)	(100.0)	NA
		I	0(0)	0(0)	2(66.7)	1(33.3)	0(0)	0(0)	0(0)	1(33.3)	0(0)	0(0)	0(0)	0(0)	0(0)	NA
		R	0(0)	0(0)	1(33.3)	1(33.3)	3(100.0)	1(33.3)	1(33.3)	0(0)	0(0)	0(0)	0(0)	0(0)	0(0)	NA
Staphylococcus aureus	26	S	25(96.2)	24(92.3)	1(3.8)	19(73.1)	NA	NA	NA	NA	NA	NA	NA	NA	NA	20(76.9)
		I	0(0)	0(0)	2 (7.7)	0(0.0)	NA	NA	NA	NA	NA	NA	NA	NA	NA	0(0)
		R	1(3.8)	2(7.7)	22(84.5)	7(26.9)	NA	NA	NA	NA	NA	NA	NA	NA	NA	6(23.1)

spp=Species, NIT=Nitrofurantoin, CIP=Ciprofloxacin, SXT = Trimethoprim-sulfamethoxazole, GEN=Gentamycin, AMP=Ampicillin, AMC=Amoxy clavulanic acid, CXT=Cefotaxime, CRO=Ceftriaxone, CAZ=Ceftazidime, MRP=Meropenem, FEF=Cefepime, AK=Amikacin, TZP = Piperacillin Tazobactam, CL=clindamycin, NA=Not applicable, other bacteria spp=*Providencia* spp (2), and *Citrobacter* spp (1).

Multi-drug resistant bacterial strains causing UTI

MDR was observed in 35/106 (33.0%) isolated organisms. A high rate of MDR was observed in *S. aureus* 11 (31.4%), *E.coli* 7(20.0%), and *Klebsiella* spp (20.0%). A high MDR above 50% was observed in combination of Ampicillin, Gentamycin, and Trimethoprim-sulfamethoxazole (Table 5).

Table 5: Patterns of MDR bacteria among children under five years attending Magu District Hospital in Mwanza region, March to April 2023.

**Antimicrobial agent**	**n (%)**	***Acinetobacter* spp**	***E. coli***	***Enterobacter* spp**	***Klebsiella* spp**	***S. aureus***	***Pseudomonas aeruginosa***	***Proteus* spp**	***Morganella* spp**
AMP, GEN, SXT	18 (51.4)	2	3	1	3	8	0	0	1
AMP, GEN, CIP	5 (14.3)	0	2	0	0	3	0	0	0
AMP, CIP, NI, AMC	3 (8.6)	1	0	0	1	0	0	1	0
AMP, CIP, MERO	2 (5.7)	0	1	0	1	0	0	0	0
AMP, GEN, CIP, NI	2 (5.7)	1	0	0	1	0	0	0	0
TZP, GEN, CAZ	5 (14.3)	0	1	0	1	0	3	0	0
**MDR, n (%)**	35/106 (33.0)	4 (11.4)	7(20.0)	1 (2.9)	7 (20.0)	11 (31.4)	3 (8.6)	1 (2.9)	1 (2.9)

AMP=Ampicillin, GEN=Gentamycin, SXT= Trimethoprim-sulfamethoxazole, CIP= Ciprofloxacin, NI=Nitrofurantoin, MERO=Meropenem, TZP= Piperacillin Tazobactam, CAZ=Ceftazidime, AMC= Amoxy clavulanic acid.

The proportion of ESBL producers and MRSA strains

The MRSA was detected in 6/26 (23.1%) of the *S. aureus*. ESBL production was detected in 19/76 (25%) of gram-negative bacteria. Predominant ESBL-producing isolates were *E. coli, Klebsiella spp* and *Acinetobacter spp* spp (Figure 1).

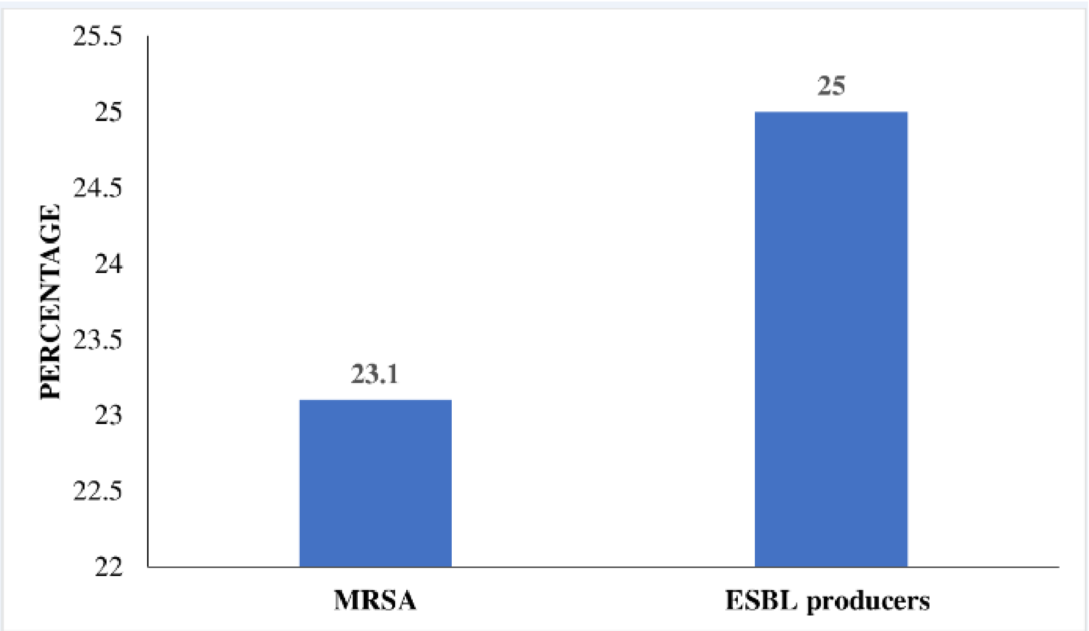

Fig 1: ESBL producers, and MRSA isolates recovered from the urine culture specimens of the febrile under-five children attending Magu District Hospital in Mwanza region, March to April 2023

Discussions

This study determined the proportion of UTI, bacterial aetiology, antimicrobial susceptibility patterns, and factors associated with UTI among under-five children. Our study found that 28.8% (95% CI -24.3-33.6) had culture-proven UTI. The proportion was lower than 39.7% reported by Festo et al. (2011) in a study conducted among under-fives in a consultant hospital in Mwanza, northwestern Tanzania (27), and 32.5% reported by Francine et al. in Cameroon (6). This study reported a higher proportion of UTI than in Kenya 11.9% (28), Mwanza Tanzania-16.8% (8), Bagamoyo-16.4% (4), Kilimanjaro-11.4% (23), and Nigeria 27.8% (16). Different risk factors can explain this variation between the current and previous studies among the population under the study, such as low hygienic standards and malnutrition (10). Additionally, only children with fever were included in this study, which may account for the higher prevalence than that conducted at Kilimanjaro, which included children with or without symptoms of UTI (23).

The common causative agent of UTI in under-five children was *E. coli*, followed by *Staphylococcus aureus*. Similar results were reported in previous studies conducted in Nigeria (2) and Cameroon (6). A study in Mwanza (8) on UTI in children also reported *E. coli* as the common isolate causing UTI. Another study at Muhimbili National Hospital, Dar es Salaam, Tanzania, reported *E. coli* as the common isolate, followed by *Klebsiella* sp*p, and Staphylococcus aureus* (15). The bacterial spectrum obtained in this study is nearly identical to that conducted on the prevalence of UTI among children in Kilimanjaro, Tanzania, which identified *E. coli, Staphylococcus aureus, Klebsiella* spp, *Proteus*, and *Pseudomonas spp* as the common aetiology of UTI (23).

Most isolates were sensitive to nitrofurantoin, ciprofloxacin, meropenem, cefepime, and piperacillin tazobactam. These results agree with similar studies conducted in Mwanza (8), Kilimanjaro (23), Dar es Salaam in Tanzania (15), and Nigeria (1) on the susceptibility patterns. The high susceptibility of isolated bacteria to meropenem may be attributed to the fact that the injectable beta-lactam antibiotic is not frequently available in Tanzanian pharmacies. As a result, meropenem is less routinely administered in Tanzania than other more widely available antibiotics. Ciprofloxacin, on the other hand, is not commonly used in children under the age of 12 years. In addition, nitrofurantoin is not recommended for use in infants younger than six weeks instead, a first-generation cephalosporin such as cephalexin may be given until the infant reaches six weeks of age.

The current study shows that isolated bacteria were somewhat sensitive to ceftriaxone, amoxicillin-clavulanate, and ceftazidime, consistent with other studies conducted in Mwanza (8) and Dar es Salaam Tanzania (15), and Ethiopia (29). The findings suggest these commonly used antibiotics to treat various bacterial infections have variations of effectiveness against certain bacteria. The susceptibility may vary depending on the specific strain and resistance mechanisms, particularly in the case of extended-spectrum beta-lactamase (ESBL)--producing organisms.

The antibiotics frequently prescribed for empirical treatment, including trimethoprim-sulfamethoxazole, piperacillin, ampicillin, gentamycin, and penicillin demonstrated resistance. These findings agree with results obtained from previous studies conducted in Mwanza (8) and Bagamoyo in Tanzania (4), and Kenya (28). However, different findings were reported in the study conducted at Muhimbili National Hospital that most *E. coli* were sensitive to gentamycin (15). The disparity in resistance rates may be attributed to the fact that these antibiotics are

readily accessible at the primary healthcare level and are, therefore, being given empirically based on clinical features, accelerating antimicrobial resistance. The results of this study highlight the significance of performing a urine culture and an antimicrobial susceptibility test on febrile children.

In this study, the overall prevalence of ESBL-producing bacteria was comparatively lower than what has been reported in previous studies conducted in Tanzania, Mwanza (30) and Dar es Salaam (22). Furthermore, the study identified the most predominant ESBL-producing isolates as *E. coli*, followed by *Klebsiella spp* and *Acinetobacter spp*. This pattern is consistent with findings from other studies conducted in Ethiopia (18). The proportion of MRSA was also lower than in previous studies conducted in Nigeria (31) and India (32). Differences in geographical locations can explain lower proportions.

The current study found that children under 24 months had a higher risk of acquiring a UTI than those older than 24 months. Similar findings were reported by previous studies conducted in Mwanza (8) and Kilimanjaro in Tanzania (23). The finding may be explained by the fact that, children under the age of 24 months are at risk of acquiring UTI due to underdeveloped immune systems, wearing of nappies for children under two years, and urinary tract abnormalities (10).

This study found that the risk of urinary tract infections in non-circumcised children was high, consistent with previous studies conducted in Dhaka (10). Another study conducted by Cason DL et al found that circumcision can help prevent recurrent urinary tract infections in hospitalized infants. A study conducted in Indonesia reported that, circumcision was associated with a decreased incidence of UTI in children (33). Additionally, a study conducted in Ethiopia found that uncircumcised male children have a higher risk of acquiring UTI compared to those who are circumcised (13).

This study revealed that symptoms of vomiting, difficulty urination, body temperature greater or equal to 39 degrees centigrade, and abdominal pain were associated with UTI in febrile children under five years. These findings align with the previous studies conducted in Mwanza (8) and Nigeria (34,35). Similar study populations, risk factors, and environmental factors may have contributed to the consistency of these findings. This study also reported that a history of recurrent UTI, chronic disease, nutrition status, and duration of fever did not show a significant association with UTI in children under five years, which agrees with other reports from Nigeria (1), England (2), and Kilimanjaro-Tanzania (23).

Limitations of the study

We were unable to ascertain the prevalence of UTI and assess antimicrobial susceptibility patterns among children who had used antibiotics in the past two weeks due to challenges in recall bias.

Conclusions

We observed a relatively high proportion of under-five children with UTI, mainly due to *Escherichia coli* and *Staphylococcus aureus*. Several factors, including a history of vomiting, dysuria, nappy use, uncircumcision, and abdominal pain could independently predict UTI in children. The isolated organisms showed high resistance to commonly prescribed antibiotics for the treatment of UTI. This highlights the growing resistance to commonly used antibiotics and emphasizes the need for continuous epidemiologic surveillance in primary healthcare facilities in Tanzania. In addition, periodic reviews of empirical treatment for UTI should be done based on the prevailing antibiotic susceptibility patterns.

Acknowledgements

We want to convey our sincere gratitude to the Management of Sekou-Toure Regional Referral Hospital and Magu District Hospital for allowing us to conduct our research under their purview. We thank Mr. Castory Dome, Mr. Philipo Shokolo, and Mr. Leoncy Kapinga for their technical assistance and directives during data and specimen collection. We express our gratitude to our study respondents who willingly participated and cooperated fully, thus facilitating the successful completion of this study.

Author Contributions

Conceptualization: RE, NL, MM

Data Curation: RE

Formal Analysis: RE, MM

Investigation: RE, NL, MM

Methodology: RE, NL, MM

Supervision: NL, SY, AJ, MM

Validation: RE, NL, SY, AJ, MM

Visualization: RE, NL, SY, AJ, MM

Writing – Original Draft Preparation: RE, NL, MM

Writing – Review & Editing: RE, NL, SY, AJ, MM

Abbreviations

ATCC: American Type Culture Collection, CFU: Colony-Forming Unit, CLSI: Clinical and Laboratory Standards Institute, ESBL: Extended Spectrum Beta Lactamase, MDR: Multi-Drug Resistant, MRSA: Methicillin-Resistant *Staphylococcus Aureus*, UTI: Urinary Tract Infection, WHO: World Health Organization.

References

1. Ibeneme C, Oguonu T, Okafor H, Ikefuna A, Ozumba U. Urinary tract infection in febrile under five children in Enugu, South Eastern Nigeria. *Nigerian Journal of Clinical Practice*, 2014; 17(5), p.624. doi:https://doi.org/10.4103/1119-3077.141430.
2. Westwood ME, Whiting PF, Cooper J, Watt IS, Kleijnen J. Further investigation of confirmed urinary tract infection (UTI) in children under five years: a systematic review. *BMC Pediatrics*, 2005; 5(1). doi:https://doi.org/10.1186/1471-2431-5-2.
3. Brien KM, Stanton N, Edwards A, Hood K, Butler CC. Prevalence of urinary tract infection (UTI) in sequential acutely unwell children presenting in primary care: exploratory study. 2011;554268; doi:https://doi.org/10.3109/02813432.
4. Sangeda RZ, Paul F, Mtweve DM. Prevalence of urinary tract infections and antibiogram of uropathogens isolated from children under five attending Bagamoyo District Hospital in Tanzania: A cross-sectional study. *F1000Research*, 2021;10, p.449. doi:https://doi.org/10.12688/f1000research.52652.1
5. Alex Fortunatus, Magufwa. Epidemiology of Urinary Tract Infection among febrile Children under five years in morogoro municipality, Tanzania. 2016.
6. Kouemo F, Guy Pascal Ngaba, Christiane D, Pradel L, Cécile Okalla Ebongue, Désiré Dieudonné Adiogo. Etiologic Profile and Sensitivity Pattern of Germs Responsible for Urinary Tract Infection Among Under-five Children in Douala, Cameroon: A Hospital-Based Study. *Avicenna Journal of Clinical Microbiology and Infection*, 2019;6(2), 10;pp.49–56. doi:https://doi.org/10.34172/ajcmi.
7. Dm K, Mosha F, Moyo S, Mi M. Etiology and Antimicrobial Susceptibility Patterns of Bacterial Agents Causing Urinary Tract Infection in Children under Five years , dar es Salaam . 2020;2(1).
8. Msaki BP, Mshana SE, Hokororo A, Mazigo HD, Morona D. Prevalence and predictors of urinary tract infection and severe malaria among febrile children attending Makongoro health centre in Mwanza city, North-Western Tanzania. *Archives of Public Health*, 2012;70(1). doi:https://doi.org/10.1186/0778-7367-70-4
9. Renko M, Salo J, Ekstrand M, Pokka T, Pieviläinen O, Uhari M *et al*. Meta-analysis of the Risk Factors for Urinary Tract Infection in Children. *Pediatric Infectious Disease Journal*, 41(10), pp.787–792. doi:https://doi.org/10.1097/inf.0000000000003628
10. Ferdaus J, Moinuddin G, Islam MT, Rashid MHO, Shawly MR, Alim A. Risk Factors of urinary tract infection in preschool children in Dhaka, Bangladesh. *Bangladesh Medical Journal*, 2017;45(3), pp.134–137. doi:https://doi.org/10.3329/bmj.v45i3.3312

11. Mohamed F. Prevalence and factors associated with urinary tract infection among febrile children admitted at Dodoma regional referral hospital. 2019.

12. Shaikh N, Morone NE, Bost JE, Farrell MH. Prevalence of urinary tract infection in childhood: a meta-analysis. *The Pediatric infectious disease journal*, [online] 2008;27(4), pp.302–8. doi:https://doi.org/10.1097/INF.0b013e31815e412,

13. Mekonnen S, Tesfa T, Tadesse Shume, Fikru Tebeje, Kedir Urgesa, Fitsum Weldegebreal. Bacterial profile, their antibiotic susceptibility pattern, and associated factors of urinary tract infections in children at Hiwot Fana Specialized University Hospital, Eastern Ethiopia. *PLOS ONE*, 2023;18(4), pp.e0283637–e0283637. doi:https://doi.org/10.1371/journal.pone.0283637.

14. Byarugaba DK. Antimicrobial resistance in developing countries and responsible risk factors. *International Journal of Antimicrobial Agents*, 2004;24(2), pp.105–110. doi:https://doi.org/10.1016/j.ijantimicag.

15. Fredrick F, Joel Msafiri Francis, Maulidi Fataki, Maselle SY. Aetiology, antimicrobial susceptibility and predictors of urinary tract infection among febrile under-fives at Muhimbili National Hospital, Dar es Salaam-Tanzania. *African Journal of Microbiology Research*,2013; 7(12), pp.1029–1034. doi:https://doi.org/10.5897/ajmr12.1866

16. Jiya N M, FWACP, Ibitoye PK, FWACP, Jiya F B M. Urinary tract infection and malaria co-morbidity in febrile children with sickle-cell anaemia in Sokoto, Nigeria 2012;20(1):299–305.

17. Said Aboud, Robinson Mdegela. Situation analysis and recommendations on Antibiotic Use and Resistance in Tanzania 2015;(March):1–83.

18. Tola MA, Abera NA, Gebeyehu YM, Dinku SF, Tullu KD. High prevalence of extended-spectrum beta-lactamase-producing Escherichia coli and Klebsiella pneumoniae fecal carriage among children under five years in Addis Ababa, Ethiopia. *PLOS ONE*, (2021), 16(10), p.e0258117. doi:https://doi.org/10.1371/journal.pone.0258117.

19. Nuru Letara, James Samwel Ngocho, Karami N, Msuya E, Balthazar Nyombi, Kassam, NA *et al*. Prevalence and patient related factors associated with Extended-Spectrum Beta-Lactamase producing Escherichia coli and Klebsiella pneumoniae carriage and infection among pediatric patients in Tanzania. *Scientific Reports*, 2021; 11(1). doi:https://doi.org/10.1038/s41598-021-02186-2

20. Onduru OG, Mkakosya RS, Rumisha SF, Aboud S. Carriage Prevalence of Extended-Spectrum β-Lactamase Producing Enterobacterales in Outpatients Attending Community Health Centers in Blantyre, Malawi. *Tropical Medicine and Infectious Disease*, 2021; 6(4) p.179. doi:https://doi.org/10.3390/tropicalmed6040179

21. Birgy A, Cohen R, Levy C, Bidet P, Courroux C, Benani M, *et al*. Community faecal carriage of extended-spectrum beta-lactamase-producing Enterobacteriaceaein french children. *BMC Infectious Diseases*, 2012;12(1). doi:https://doi.org/10.1186/1471-2334-12-315.

22. Tellevik MG, Blomberg B, Kommedal Ø, Maselle SY, Langeland N, Moyo S.J. High Prevalence of Faecal Carriage of ESBL-Producing Enterobacteriaceae among Children in Dar es Salaam, Tanzania. *PLOS ONE*, 2016;11(12), p.e0168024. doi:https://doi.org/10.1371/journal.pone.0168024

23. Gidabayda J, Philemon R, S Abdallah M, M Saajan A, Temu T, Kunjumu *et al*. Prevalence, Aetiology, and Antimicrobial Susceptibility Patterns of Urinary Tract

Infection Amongst Children Admitted at Kilimanjaro Christian Medical Centre, Moshi, Tanzania. *East African Health Research Journal*, 2017;1(1), pp.53–61. doi:https://doi.org/10.24248/eahrj.v1i1.388

24. Assessment on how to Calculate Weight-for-Height Z-Score (WHZ) in C hildren 0 − 59 Months of Age. :6–9

25. CLSI. Performance Standards for Antimicrobial Susceptibility Testing. M100, 30th ed. 2022; 40(1).

26. Abdelmoktader A, Talal A, Far E. Methods of ESBLs Detection in Clinical Microbiology Lab. 2019;1–8.

27. Festo E, Kidenya BR, Aldofina Hokororo, Mshana. Predictors of Urinary tract infection among febrile children attending at Bugando Medical Centre Northwestern, Tanzania. *Archives of Clinical Microbiology*, 2011;2(5).

28. Masika WG, Meara WP, Holland TL, Armstrong. Contribution of urinary tract infection to the burden of febrile illnesses in young children in rural Kenya. *PLOS ONE*, 2017;12(3), p.e0174199. doi:https://doi.org/10.1371/journal.pone.0174199

29. Marami D, Balakrishnan S, Seyoum B. Prevalence, Antimicrobial Susceptibility Pattern of Bacterial Isolates, and Associated Factors of Urinary Tract Infections among HIV-Positive Patients at Hiwot Fana Specialized University Hospital, Eastern Ethiopia. *Canadian Journal of Infectious Diseases and Medical Microbiology*, 2019, pp.1–8. doi:https://doi.org/10.1155/2019/6780354

30. Ahmed M, Moremi N, Mirambo MM, Hokororo A, Mushi MF, Seni J *et al*. Multi-resistant gram negative enteric bacteria causing urinary tract infection among malnourished underfives admitted at a tertiary hospital, northwestern, Tanzania. *Italian Journal of Pediatrics*, 2015;41(1). doi:https://doi.org/10.1186/s13052-015-0151-5

31. Abubakar U, Sulaiman SAS. Prevalence, trend and antimicrobial susceptibility of Methicillin Resistant Staphylococcus aureus in Nigeria: a systematic review. *Journal of Infection and Public Health*, 2018;pp.763–770. doi:https://doi.org/10.1016/j.jiph.

32. Arora S, Devi P, Arora U, Devi B. Prevalence of Methicillin-resistant Staphylococcus Aureus (MRSA) in a Tertiary Care Hospital in Northern India. *Journal of Laboratory Physicians*, 2010;2(02), pp.078–081. doi:https://doi.org/10.4103/0974-2727.72154.

33. Irfan Wahyudi, Putu Angga Risky Raharja, Gerhard Reinaldi Situmorang and Arry Rodjani. Circumcision reduces urinary tract infection in children with antenatal hydronephrosis: Systematic review and meta-analysis. *Journal of Pediatric Urology*, 2023;19(1), pp.66–74. doi:https://doi.org/10.1016/j.jpurol.2022.10.029

34. Okunola P, Ibadin M, Ofovwe G. Predicting Urinary Tract Infection in Malaria Co-morbidity among Under Fives. *Nigerian Hospital Practice*, 2011;7(3-4). doi:https://doi.org/10.4314/nhp.v7i3-4.67124

35. Estefanía J, Regalado A, Brito MO, Enrique L. Prevalence of urinary tract infection and associated factors in hospitalized patients aged 0 to 5 years . 2021;22(1):1–8.

Click here to access/download
Supporting Information
RESEARCH DATA-UTI in underfive children.xlsx

Bacterial aetiology, Antimicrobial Susceptibility Patterns, and Factors Associated with Urinary Tract Infection among Under-five Children at Primary Health Facility, North-Western Tanzania

Roza Ernest[1,2*], Nsiande Lema[1], Sued Yassin[3], Agricola Joachim[4], Mtebe Majigo[4].

[1] Tanzania Field Epidemiology and Laboratory Training Program, Dar es Salaam, Tanzania
[2] Department of Epidemiology and Biostatistics, Muhimbili University of Health and Allied Sciences, Dar es Salaam, Tanzania
[3] Research, Training, and Consultancy Unit, Chato Zonal Referral Hospital, Geita, Tanzania
[4]Department of Microbiology and Immunology, Muhimbili University of Health and Allied Sciences, Dar es Salaam, Tanzania

***Email:** roza.ernest@yahoo.com (RE)

Abstracts

Background: Urinary tract infection is common in under-five children presenting with fever with significant consequences leading to bacteremia, dehydration, kidney scarring, and renal failure. The incidence of Urinary tract infection varies with age, gender, health condition, and geographic location. There is a lack of studies addressing these issues, particularly concerning children. This study determined the proportion of urinary tract infections, bacterial aetiology, antimicrobial susceptibility patterns, and factors associated with urinary tract infection among under-five children.

Materials and methods: The cross-sectional analytical study was conducted at the District Hospital in Mwanza Region, North-Western Tanzania, between March and April 2023. A convenient non-probability sampling technique was used to collect urine samples from children under five years old with signs and symptoms of urinary tract infection. Written informed consent was obtained from all parents or guardians. Participants' information was collected using a pretested structured questionnaire. Urine samples were processed at Sekou Toure Regional Referral Hospital. STATA version 15.0 was used for data analysis. Factors associated with Urinary tract infection were determined using a Modified Poisson Model.

Result: The study recruited 368 under-five children; 194 (52.7%) were males. The median age (interquartile range) was 24 (13-36) months. Of all, 28.8% (95% CI-24.3-33.6) had urinary tract infections. One hundred and six pathogens were isolated, the majority being *Escherichia coli*, 37 (34.9%), and *Staphylococcus aureus*, 26 (24.5%). *Escherichia coli* were susceptible to cefepime, piperacillin-tazobactam, nitrofurantoin, and meropenem, ranging from 81.1% to 97.3%. *Staphylococcus aureus* was susceptible to nitrofurantoin (96.2%) and ciprofloxacin (92.3%). Multidrug resistance was observed in 33.0% of isolates, Methicillin-Resistance *Staphylococcus aureus* in 23.1% of *Staphylococcus aureus*, and extended-spectrum beta-lactamases in 25% of gram-negative bacteria. Urinary tract infection was associated with vomiting, age, dysuria, abdominal pain, nappy use, and uncircumcision in males.

Conclusion: Our study found a relatively high proportion of urinary tract infections among under-five children. *Escherichia coli* and *Staphylococcus aureus* were the common isolated organisms susceptible to nitrofurantoin, ciprofloxacin, meropenem, cefepime, and piperacillin-tazobactam, and least susceptible to commonly used antibiotics. Empirical treatment of urinary tract infection should be reviewed periodically based on the antibiotic susceptibility pattern.

Keywords: Urinary Tract Infection, Antimicrobial susceptibility pattern, under-fives children

Introduction

Urinary tract infection (UTI) is common in under-five children presenting with fever with significant consequences leading to bacteremia, dehydration, kidney scarring, and renal failure (1). Early diagnosis is essential to preserve and maintain the proper functioning of the kidneys and prevent any further decline in their function (2,3). After acute respiratory infections, UTI has been reported as the second most typical site of infection in the community, and incidence varies with age, gender, and health condition. Studies conducted in Nigeria and Tanzania have reported the prevalence of UTI in under-five children ranging from 7.4% to 43.6% (1,4,5).

UTI in children is primarily caused by bacteria, but other pathogens such as viruses, fungi, and parasites can also be responsible. The most common bacteria associated with UTI in children include gram-negative pathogens such as *Escherichia coli, Klebsiella, Proteus, Enterobacter, Pseudomonas,* and *Serratia*. Gram-positive pathogens associated with UTI in children include

group B *streptococcus, enterococcus* spp, and *Staphylococcus aureus*. In addition to bacterial causes, non-bacterial causes include viruses causing viral haemorrhagic cystitis and Candida infection, particularly in individuals with compromised immune systems (4,6,7). The bacteria causing UTI have varied antibiotic susceptibility patterns, most resistant to commonly used antibiotics (4,8).

The risk factors for acquiring UTI in under-five children are multiple and differ with geographical locations, settings, and seasons (9). The commonly reported factors include female gender, poor hygiene, poverty, malnutrition, and recurrent infections (10,11). Other predisposing factors include age, uncircumcision in males (12), genitourinary anomalies, indwelling urinary catheters, and previous antibiotic use (13).

Urine culture is rarely performed as part of patient management in rural areas with inadequate diagnostic resources. Treatment is empirical, which could encourage the development and spread of antimicrobial-resistant bacteria (14). Antimicrobial resistance (AMR) has become a significant concern worldwide (14). Recent research has shown a high prevalence of resistance to commonly used antibiotics (14–16). Antimicrobial resistance is also prevalent in Tanzania, with reports indicating multi-drug resistance among urinary pathogens associated with UTIs (17).

Extended-spectrum beta-lactamases (ESBL) producers contribute a high resistance rate to all beta-lactam antibiotics and are more common in hospitals than in the community or outpatient settings (17,18). ESBL-producing bacteria prevalence of infection or colonisation among children under five years ranges from 17% to 35% (18–21). Studies in Tanzania reported that ESBL producers' prevalence ranges from 18% to 45%; Carbapenems were reported as the drugs to treat ESBL-producing bacteria (17,22). However, they are expensive and not widely available in Tanzania.

Therefore, increasing knowledge of the aetiologic profile and antimicrobial susceptibility patterns of pathogens associated with UTI in under-fives is essential in medical practices. Understanding the associated factors also helps identify at-risk groups and institute preventive measures. This research paper explored the etiologic profile, antimicrobial susceptibility pattern, and factors associated with UTI among under-five children in Magu District Hospital, Mwanza-Tanzania.

Materials and Methods

Study design, setting, and population

We conducted a cross-sectional analytical study design between 8th March and 14th April 2023 at Magu District Hospital in Mwanza Region, North-Western Tanzania. Magu district is located between 33° and 34° East of Greenwich and between 2°10′ and 2°50′ South of the Equator. Its total area is 3075 km2, of which Lake Victoria waters cover 1725 km2 (56.1%). The average annual rainfall is between 700 and 1000 mm. As of 2012, the population of Magu district was 299,759 people. Magu District Hospital is a referral hospital in the district. Over 40% of the patient population seeking services is under-fives children. The laboratory procedures were conducted at Sekou Toure Regional Referral Hospital Laboratory.

The study involved under-five children with a fever seeking healthcare services. We recruited children with fever who the attending clinician suspected to have UTI based on signs and symptoms such as fever of 38 degrees Celsius or above, pain or burning when urinating, frequent urination, discoloured urine, lower abdominal pain, or vomiting. The study did not include children who used catheters within 72 hours because catheter insertion may directly inoculate microorganisms into the urinary bladder.

Sample Size and Sampling Technique

We estimated the minimum sample size of 355 under five children using the Kish Leslie formula considering the 11.4% previously reported prevalence of UTI among under-five children (23). A non-probability convenient sampling technique was used where all febrile under-five children attending Magu District Hospital were recruited in the study after obtaining consent from the parent/guardian.

Data collection

The participants' socio-demographic (age, sex, and place of residence), clinical characteristics (vomiting, dysuria, body temperature, abdominal pain, and chronic disease), and risk factors (nutrition status, history of recurrent UTI, male circumcision, duration of fever, and nappy use) were collected using a structured and pre-tested questionnaire. Axillary temperature was measured using a digital thermometer. The children's body weight was measured using a digital weighing scale. The nutrition status of the children was determined by calculating their weight-for-age z-scores (WAZ), which represent how many standard deviations their weight is from the median weight of a healthy reference population of the same age and sex. To assess their

nutritional status, a WAZ below -3 standard deviations was used to indicate malnutrition, while a WAZ between -2 and +2 standard deviations was used to indicate a normal nutritional status (well-nourished) (24).

Sample collection

A urine specimen for each participant was collected. After receiving adequate instruction, the study participant's parents or guardians assisted with the urine collection. Approximately 10ml of urine specimens were collected into a sterile, wide-mouth, leak-proof, screw-capped plastic container pre-labelled with the participant's identification number, age, date, and time. The specimen was transported daily to Sekou Toure Regional Referral Hospital laboratory for processing within two hours and kept in a cool box. Immediate inoculation of samples was done on arrival at the laboratory. If a delay was inevitable, specimens were refrigerated at 4°C not more than 12 hours.

Definition of terms

For the purpose of the current study, multidrug resistance (MDR) was defined as resistance to at least one antimicrobial agent in three or more antimicrobial categories. Methicillin-resistant *Staphylococcus aureus* was defined as an isolate of staphylococcus aureus with an inhibition zone of less than or equal to 21mm diameter around the 30 µg cefoxitin disk on antimicrobial susceptibility disk diffusion method, and Extended-spectrum beta-lactamases producers was defined as gram negative bacteria that shows an increase in the inhibition zone towards the amoxicillin-clavulanic acid disk in either the cefotaxime disk or ceftazidime disk after placing. Ceftazidime (30µg), cefotaxime (30µg), and amoxicillin-clavulanic acid (20/10 µg) disks in a straight line 20 mm apart on the Muller Hinton plate, with amoxicillin-clavulanic acid in the center.

Urine Culture

We performed a quantitative urine culture to determine the significant bacteriuria. Urine samples were inoculated into Cysteine Lactose-Electrolyte Deficient (CLED) (Sigma Alorich, India) agar plates using a calibrated wire loop (0.001mL). Culture plates were incubated under aerobic conditions at 37°C for 24 hours. Bacterial growth of $\geq 10^5$ colony-forming unit (CFU)/mL of urine was considered significant bacteriuria. Specimens yielding fewer than 10^5

CFU/mL were considered insignificant or potentially contaminated. In instances of mixed bacterial growth, subculturing was conducted to isolate each organism separately.

Bacteria identification

We used conventional methods to identify isolated bacteria, including colony characteristics, gram staining reaction, and biochemical testing. Sulphur Indole Motility (SIM) (Himedia, India), Kligler Iron Agar test (Oxoid Ltd, UK), urease test (Oxoid Ltd, UK), and citrate utilisation test (Oxoid Ltd, UK) were used to identify gram-negative bacteria. Oxidase test (Himedia, India) was used to identify *pseudomonas* and *Acinetobacter*. Catalase and coagulase tests were used to identify gram-positive bacteria.

Antimicrobial susceptibility testing

Antimicrobial susceptibility testing was performed using the Kirby-Bauer disk diffusion method adhering to Clinical and Laboratory Standards Institute (CLSI) guidelines (25). The selection of antibacterial disks was based on CLSI 2022 recommendations and availability. The bacterial inoculum, equivalent to 0.5 McFarland standards, was uniformly spread onto Mueller–Hinton agar plates (Himedia India) using a sterile cotton swab applicator. Antibiotic disks for gram-positive bacteria were penicillin G (10 μg), nitrofurantoin (300 μg), ciprofloxacin (5μg), trimethoprim-sulfamethoxazole (1.25/23.75 μg), gentamicin (10 μg), cefoxitin (30 μg), and clindamycin (30 μg) (Himedia, India).

The antimicrobial disks for Enterobacteriaceae included piperacillin-tazobactam (100/10 μg), ampicillin (10 μg), nitrofurantoin (300 μg), amoxicillin-clavulanate (20/10 μg), gentamicin (10 μg), cefotaxime (30 μg), ciprofloxacin (30 μg), meropenem (10 μg), piperacillin (100 μg), cefepime (30 μg), amikacin (30 μg), trimethoprim-sulfamethoxazole (1.25/23.75 μg), ceftazidime (30 μg), and ceftriaxone (30 μg) (Himedia, India). Antimicrobial disks for *Pseudomonas* spp and *Acinetobacter* were gentamicin (10 μg), meropenem (10 μg), ceftazidime (30 μg), piperacillin-tazobactam (100/10 μg), ciprofloxacin (5 μg), and amikacin (30 μg) (Himedia, India).

Extended-Spectrum Beta-Lactamase Detection

Gram-negative bacteria producing ESBL was first suspected from the result of antimicrobial susceptibility testing based on the diameter of the zone of inhibition for the three third-generation cephalosporins: cefotaxime (30μg), ceftriaxone (30μg) and ceftazidime (30μg). The

following breakpoints were used to suspect ESBL production: ceftriaxone ≤ 25mm, ceftazidime ≤ 22mm, and cefotaxime ≤ 27mm.

We confirmed ESBL production using the Double Disk Synergy method (26). Ceftazidime (30µg), cefotaxime (30µg), and amoxicillin-clavulanic acid (20/10 µg) disks (Himedia, India) were placed in a straight line 20 mm apart on the plate, with amoxicillin-clavulanic acid in the center. An increase in the inhibition zone towards the amoxicillin-clavulanic acid disk in either the cefotaxime disk or ceftazidime disk was a positive result for ESBL enzyme production; the isolate was reported as an ESBL producer.

Detection of methicillin-resistant *Staphylococcus aureus*

Methicillin-resistant *Staphylococcus aureus* (MRSA) was phenotypically determined using a cefoxitin (30 µg) disk (Oxoid Ltd, UK) per CLSI guidelines (25). A cefoxitin disk was applied and incubated at 37^0C for 18 to 24 hours. A less or equal to 21 mm inhibition zone diameter around the cefoxitin disk indicated MRSA.

Quality assurance

Media and reagents were checked by verifying the expiration date, storage conditions, and visual inspection of any signs of contamination, such as discolouration, turbidity, or particulate matter. The media quality was assessed using sterility and performance tests using reference strains. Reference strains for culture and susceptibility testing included *P. aeruginosa* (ATCC 27853), *S. aureus* (ATCC 25923), and *E. coli* (ATCC 25922). Additionally, *E. coli* (ATCC 25922) and *K. pneumonia* (ATCC 700603) ESBL-positive strains were used to control ESBL production. *S. aureus* (ATCC 43300) was used as a positive control for MRSA.

Data analysis

Data analysis was done using STATA version 15 StataCorp.2017. Frequency and proportions were used to summarise the characteristics of study populations for categorical variables, whereas median and interquartile range were used to summarise continuous variables. Pearson's chi-square test was used to compare a categorical variable to the status of UTI. The modified Poisson model was performed to determine factors associated with UTI. The multivariable analysis involved variables with a p-value ≤ 0.2 to determine the adjusted prevalence ratio and 95% confidence intervals. A p-value < 0.05 was used in both models to acknowledge statistical significance.

Ethical approval

The study obtained ethical clearance number MUHAS-REC-12-2022-1484 from the Muhimbili University of Health and Allied Sciences Senate Research and Publications Committee. In addition, we got permission for data collection from the offices of the Regional and District Medical Officers. Parents or guardians were informed about the study objectives and consented on behalf of their children before inclusion in the study. Results of urine culture and antimicrobial susceptibility tests were timely communicated to the attending clinician for patient management. Participant identification numbers were used in the study instead of names to ensure confidentiality.

Results

Socio-demographic and clinical characteristics

A total of 368 under-five children were enrolled in the study. Males counted for more than half of the participants, 194 (52.7%). The study participants' median age (interquartile range) was 24 (13-36) months. Most participants, 296 (80.4%), lived in rural areas. Malnutrition was observed in 27 (7.3%) and 29 (14.9%) males were already circumcised. Most participants, 201 (54.6%), had a fever for 3 to 7 days, and the majority, 348 (94.6%), had a 38-39 ^{0}C temperature. Abdominal pain was observed in 153 (41.6%), while 61 (16.6%) had dysuria. Vomiting was observed in 84 (22.8%) participants, and 24 (6.5%) had a history of recurrent UTI (Table 1).

The proportion of UTI among under-five children

UTI was detected in 28.8% (106/368, 95% CI -24.3-33.6), more in children below 24 months, 53 (35.1%), than in children aged 24 months and above, 53 (24.4%) (p = 0.026). UTI was significantly higher in children with dysuria, 30 (49.2%) compared to those without dysuria, 76 (24.8%) ($p < 0.001$). The proportion of UTI was higher among children with a fever of 3-7 days 68 (33.8%) compared to those with a fever of ≤ 2 days 33 (21.3%) and greater than 7 days 5 (41.7%) ($p = 0.021$). Children presented with abdominal pain were more diagnosed with UTI, 81 (52.9%), than those without abdominal pain, 25 (11.6%) ($p < 0.001$). The proportion of UTI was found to be higher among children who used nappies, 56 (34.8%), than in children who did not use nappies, 50 (24.2%) (p=0.026). Uncircumcised male children had more UTI, 55 (33.3%) than circumcised children, 3 (10.3%) (p = 0.030). The proportion of UTI was significantly higher in children presented with vomiting 37 (44.0%) than in those without vomiting 69 (24.3%) ($p < 0.001$). The proportion of UTI was higher in children with a body

temperature above 39°c, 11 (55.0%) than in children with a body temperature between 38-39 ^{O}C, 95 (27.3%) (*p*=0.008). There was no difference in UTI proportion based on sex, history of recurrent UTI, place of residence, nutrition status, or chronic disease, and the p-value was greater than 0.05 (Table 1).

Table 1: Proportion of UTI among the febrile under-five children attending Magu District Hospital in Mwanza region, March to April 2023

Variable	Description	Frequency N (%) *	UTI negative n (%)**	UTI positive n (%) ***	P value
**Age group (months)**	<24	151(41.0)	98 (64.9)	53 (35.1)	0.026
	≥24	217(59.0)	164 (75.6)	53 (24.4)	
**Sex**	Male	194 (52.7)	136 (70.1)	58 (29.9)	0.625
	Female	174 (47.3)	126 (72.4)	48 (27.6)	
**Place of residence**	Urban	72 (19.6)	52 (72.6)	20 (27.8)	0.830
	Rural	296 (80.4)	210 (70.9)	86 (29.1)	
**Duration of fever (days)**	≤2	155 (42.1)	122 (78.8)	33 (21.3)	0.021
	3-7	201 (54.6)	133 (66.2)	68 (33.8)	
	>7	12 (3.3)	7 (58.3)	5 (41.7)	
**Axillary Temperature**	38-39	348 (94.6)	253 (72.7)	95 (27.3)	0.008
	>39	20 (5.4)	9 (45)	11 (55.0)	
**Dysuria**	Yes	61 (16.6)	31 (50.8)	30 (49.2)	< 0.001
	No	307 (83.4)	231 (75.2)	76 (24.8)	
**Vomiting**	Yes	84 (22.8)	47 (56)	37 (44.0)	< 0.001
	No	284 (77.2)	215 (75.7)	69 (24.3)	
**Abdominal pain**	Yes	153 (41.6)	72 (47.1)	81 (52.9)	< 0.001
	No	215 (58.4)	190 (88.4)	25 (11.6)	
**Recurrent UTI**	Yes	24 (6.6)	17 (70.8)	7 (29.2)	0.968
	No	344 (93.5)	245 (71.2)	99 (28.8)	
**Use of nappy**	Yes	161 (43.8)	105 (65.2)	56 (34.8)	0.026
	No	207 (56.2)	157 (75.8)	50 (24.2)	
**Chronic disease**	Yes	8 (2.2)	6 (75)	2 (25.0)	0.810
	No	360 (97.8)	256 (71.1)	104 (28.9)	
**Nutrition status**	Malnourished	27 (7.3)	17 (63)	10 (37.0)	0.326
	Well-nourished	341 (92.7)	245 (71.8)	96 (28.2)	
**Male circumcision (n = 194)**	Yes	29 (14.9)	26 (89.7)	3 (10.3)	0.030
	No	165 (85.1)	110 (66.7)	55 (33.3)	

*UTI=Urinary Tract Infection, *Frequency and percentage of participants calculated as number within the variable category divided by the total sample size of the variable,*

*** proportion of patients within the variable category with no Urinary Tract Infection calculated as number of divided by the total number of individuals in the variable category*

**** proportion of patients within the variable category with Urinary Tract Infection calculated as number of cases divided by the total number of individuals in the variable category*

Uropathogens bacteria causing UTI

A total of 106 pathogenic bacteria strains were isolated from 106 urine specimens of febrile under-five children. No multiple infection was obtained with significant growth of bacteria counts of ≥10^5 CFU/ml. Gram-negative bacteria constituted 80 (75.48%), while gram-positive bacteria counted 26 (24.52%). *E. coli* was the most common isolated pathogen, 37 (35%), followed by *S.aureus* 26/106 (25%) (Table 2).

Table 2: Frequency of bacteria isolates among children under five years attending Magu District Hospital in Mwanza region, March to April 2023

Isolated bacteria	Frequency	Percentage
Escherichia coli	37	34.9
Staphylococcus aureus	26	24.5
Enterobacter spp	10	9.4
Klebsiella spp	12	11.3
Acinetobacter spp	5	4.7
Morganella morganii	5	4.7
Proteus spp	4	3.8
Pseudomonas aureginosa	4	3.8
Providencia spp	2	1.9
Citrobacter spp	1	0.9

Factors Associated with Urinary Tract Infection

In univariate analysis, age, vomiting, dysuria, body temperature, abdominal pain, use of nappy, and uncircumcision were associated with UTI. Children below 24 months (CPR 1.4; 95% CI: 1.0–1.9, $P = 0.03$), who used nappies (CPR 1.4; 95% CI: 1.0–1.9, $P = 0.026$), and those presented with vomiting (CPR 1.8; 95% CI: 1.3–2.5, $P < 0.001$), dysuria (CPR 1.9; 95% CI: 1.4–2.7, $P < 0.001$), and body temperature above 39°C (CPR 2.0; 95% CI: 1.4–2.7, $P = 0.02$) were more likely to have UTI than their counterparts, with the prevalence ranging between 1.4 and 2.0, $p < 0.05$. Children with abdominal pain had 4.6 times more UTI than those without (CPR 4.6; 95%CI:3.1-6.8, $p < 0.001$). Uncircumcised children had 3.2 times more chances of acquiring UTI than circumcised (CPR 3.2; 95% CI: 1.1–9.6, $P = 0.035$). Sex, duration of fever, history of recurrent UTI, place of residence, nutrition status, and a history of having chronic disease were not associated with UTI.

Eight factors were included in the multivariable analysis. Age below 24 months, abdominal pain, vomiting, dysuria, body temperature above 39°C, use of nappy, and uncircumcision were independently associated with UTI. Children below 24 months had 1.5 times the prevalence of

acquiring UTI compared to those aged 24 months and above (APR= 1.5, 95% CI = 1.1–2.1, *P* = 0.010). Furthermore, children with abdominal pain had 3.9 times the prevalence of having a UTI than those without abdominal pain (APR = 3.9, 95% CI = 2.6–5.9, *P* < 0.001). Having vomiting, dysuria, or a body temperature above 39°C increased the prevalence of having UTI by 1.6 to 1.7 times. Using a nappy increased the prevalence of acquiring UTI by 1.2 times more than not using a nappy (APR 1.2; 95% CI: 0.6–2.4, *P* = 0.003). Uncircumcised children had 2.5 times the prevalence of acquiring UTI than circumcised (APR 2.5; 95% CI: 0.4–8.2, *P* = 0.002) (Table 3).

Table 3: Factors associated with UTI among febrile children under five years in Magu District -Mwanza region, March to April 2023

Variable	UTI negative n (%)	UTI positive n (%)	Bivariate analysis		Multivariate analysis	
			CPR (95% CI)	*P* value	APR (95%CI)	*P* value
**Age group (months)**						
<24	98 (64.9)	53 (35.1)	1.4 (1.0-1.9)	0.030	1.5 (1.1-2.1)	0.010
≥24	164(75.6)	53 (24.4)	1			
**Sex**						
Male	136 (70.1)	58 (29.9)	1.0 (0.8-1.5)	0.625		
Female	126 (72.4)	48 (27.6)	1			
**Place of residence**						
Urban	72 (72.2)	20 (27.8)	1			
Rural	296 (78.9)	86 (29.1)	1.0 (0.7-1.6)	0.831		
**Duration of fever (days)**						
≤2	122 (78.8)	33 (21.3)	1			
3-7	133 (66.2)	68 (33.8)	1.5 (1.1-2.1)	0.020	1.4 (0.9-1.9)	0.057
>7	7 (58.3)	5 (41.7)	1.5 (0.7-2.9)	0.275	1.5 (0.7-2.9)	0.179
**Temperature**						
38-39	253 (72.7)	95 (27.3)	1			
>39	9 (45.0)	11 (55.0)	2.0 (1.3-3.1)	0.002	1.7 (1.1-2.8)	0.020
**Dysuria**						
Yes	31 (50.8)	30 (49.2)	1.9 (1.4-2.7)	< 0.001	1.6 (1.2-2.3)	0.010
No	231 (75.2)	76 (24.8)	1			
**Vomiting**						
Yes	47 (56.0)	37 (44.0)	1.8 (1.3-2.5)	< 0.001	1.6 (1.2-2.2)	0.002
No	215 (75.7)	69 (24.3)	1			
**Abdominal pain**						
Yes	72 (47.1)	81 (52.9)	4.6 (3.1-6.8)	< 0.001	3.9 (2.6-5.9)	< 0.001
No	190 (88.4)	25 (11.6)	1			
**Recurrent UTI**						
Yes	17 (70.8)	7 (29.2)	1.0 (0.5-1.9)	0.968		
No	245 (71.2)	99 (28.8)	1			
**Use of nappy**						
Yes	105 (65.2)	56 (34.8)	1.4 (1.0-1.9)	0.026	1.2 (0.6-2.4)	0.003
No	157 (75.8)	50 (24.2)	1			
**Chronic disease**						
Yes	6 (75)	2 (25.0)	0.9 (0.3-2.9)	0.815		

No	256 (71.1)	104 (28.9)	1			
**Nutrition status**						
Malnourished	17 (63)	10 (37.0)	1.3 (0.8-2.2)	0.302		
Well-nourished	245 (71.8)	96 (28.2)	1			
**Male circumcision** (n = 194)						
Yes	26 (89.7)	3 (10.3)	1			
No	110 (66.7)	55 (33.3)	3.2 (1.1-9.6)	0.035	2.5 (0.4-8.2)	0.002

UTI: urinary tract infection, CPR: Crude Prevalence Ratio, APR: Adjusted Prevalence Ratio

Antimicrobial susceptibility patterns of isolated bacteria

Escherichia coli were susceptible to cefepime, piperacillin-tazobactam, nitrofurantoin, and meropenem, ranging from 81.1% to 97.3%, while least susceptible to trimethoprim-sulfamethoxazole (37.8%), gentamycin (32.4%), and ampicillin (18.9%). *Staphylococcus aureus* was susceptible to nitrofurantoin (96.2%) and ciprofloxacin (92.3%), and least susceptible to penicillin (11.5%). Susceptibility to ciprofloxacin ranges from 40% (*Acinetobacter* spp) to 100% *Pseudomonas aeruginosa*, *Citrobacter* spp, *Morganella morganii*, *Proteus* spp, and *Providencia* spp. Susceptibility to meropenem ranges from 20% *Acinetobacter* to 100% *Enterobacter* species. Trimethoprim-sulfamethoxazole susceptibility ranges from (37.8% *E. coli)* to 70% (*S. aureus)* (Table 4).

Table 4: Antimicrobial Susceptibility patterns of bacteria causing UTI among children under five years attending Magu District Hospital in Mwanza region, March to April 2023.

**Bacteria isolates**	**N**	**Pattern**	**NIT**	**CIP**	**SXT**	**GEN**	**AMP**	**AMC**	**CTX**	**CRO**	**CAZ**	**MRP**	**FEF**	**TZP**	**AK**	**CL**
		S	32(86.5)	28(75.7)	14(37.8)	12(32.4)	7(18.9)	25(67.6)	25(67.5)	29(78.4)	27(72.9)	36(97.3)	30(81.1)	32(86.5)	28(75.7)	NA
	37	I	2(5.4)	5(13.5)	8(21.6)	1(2.7)	13(35.1)	6(16.2)	8(21.6)	4(10.8)	4(10.8)	0(0)	4(10.8)	4(10.8)	6(16.2)	NA
E. coli		R	3(8.1)	4(10.8)	15(40.5)	24(64.9)	17(45.9)	6(16.2)	4(10.8)	4(10.8)	6(16.2)	1(2.7)	3(8.1)	1(2.7)	3(8.1)	NA
Klebsiella spp	12	S	11(91.7)	10(83.3)	8(66.7)	3(25.0)	(0.0)	7(58.3)	6(50.0)	7(58.4)	8(66.7)	11(91.7)	10(83.3)	10(83.3)	8(66.7)	NA
		I	1(8.3)	1(8.3)	0(0)	3(25.0)	2(16.7)	3(25.0)	3(25.0)	1(8.3)	0(0)	0(0)	1(8.3)	1(8.3)	0(0)	NA
		R	0(0)	1(8.3)	4(33.3)	6(50.0)	10(83.3)	2(16.7)	3(25.0)	4(33.3)	4(33.3)	1(8.3)	0(0)	1(8.3)	4(33.3)	NA
Enterobacter spp	10	S	8(80.0)	9(90.0)	6(60.0)	3(30.0)	3(30.0)	10(90.0)	6(60.0)	7(70.0)	9(90.0)	10(100.0)	8(80.0)	9(90.0)	9(90.0)	NA
		I	1(10.0)	1(10.0)	1(10.0)	0()	1(10.0)	1(10.0)	4(40.0)	2(20.0)	0(0)	0(0)	2(20.0)	0(0)	0(0)	NA
		R	1(10.0)	0()	3(30.0)	7(70.0)	6(60.0)	0(0)	0(0)	1(10.0)	1(10.0)	0(0)	0(0)	1(10.0)	1(10.0)	NA
Pseudomonas aeruginosa	4	S	NA	4(100.0)	NA	0(0.0)	NA	NA	NA	NA	1(25.0)	4(100.0)	4(100.0)	4(100.0)	4(100.0)	NA
		I	NA	0(0.0)	NA	0(0.0)	NA	NA	NA	NA	2(50.0)	0(0.0)	0(0.0)	0(0.0)	0(0.0)	NA
		R	NA	0(0.0)	NA	4(100.0)	NA	NA	NA	NA	1(25.0)	0(0.0)	0(0.0)	0(0.0)	0(0.0)	NA
Acinetobacter spp	5	S	NA	5(100.0)	NA	1(20.0)	NA	NA	NA	NA	1(20.0)	5(100.0)	5(100.0)	4(80.0)	4(80.0)	NA
		I	NA	0(0)	NA	0(0)	NA	NA	NA	NA	1(20.0)	0(0)	0(0)	1(20.0)	0(0)	NA
		R	NA	0(0)	NA	4(80.0)	NA	NA	NA	NA	3(60.0)	0(0)	0(0)	0(0)	1(20.0)	NA
Morganella spp	5	S	3(60.0)	5(100)	4(80.0)	2(40.0)	1(20.0)	3(60.0)	3(60.0)	3(60.0)	4(80.0)	4(80.0)	5(100.0)	5(100.0)	5(100.0)	NA
		I	1(20)	0(0)	0(0)	0(0)	0(0)	1(20)	1(20)	0(0)	0(0)	1(20)	0(0)	0(0)	0(0)	NA
		R	1(20)	0(0)	1(20)	3(60)	4(80)	1(20)	1(20)	2(40.0)	1(20)	0(0)	0(0)	0(0)	0(0)	NA
Proteus spp	4	S	3(75.0)	4(100.0)	3(75.0)	1(25.0)	0.0	3(75.0)	2(50.0)	3(75.0)	4(100.0)	3(75.0)	4(100.0)	4(100.0)	3(75.0)	NA
		I	0(0)	0(0)	0(0)	0(0)	0(0)	0(0)	1(25.0)	0(0)	0(0)	0(0)	0(0)	0(0)	0(0)	NA
		R	1(25.0)	0(0)	1(25.0)	3(75.0)	4(100)	1(25.0)	1(25.0)	1(25.0)	0(0)	1(25.0)	0(0)	0(0)	1(25.0)	NA
Other bacteria spp	3	S	3(100.0)	3(100.0)	0(0)	1(33.3)	0(0.0)	2(66.7)	(66.7)	(66.7)	(100.0)	(100.0)	(100.0)	(100.O)	(100.0)	NA
		I	0(0)	0(0)	2(66.7)	1(33.3)	0(0)	0(0)	0(0)	1(33.3)	0(0)	0(0)	0(0)	0(0)	0(0)	NA
		R	0(0)	0(0)	1(33.3)	1(33.3)	3(100.0)	1(33.3)	1(33.3)	0(0)	0(0)	0(0)	0(0)	0(0)	0(0)	NA
Staphylococcus aureus	26	S	25(96.2)	24(92.3)	1(3.8)	19(73.1)	NA	NA	NA	NA	NA	NA	NA	NA	NA	20(76.9)
		I	0(0)	0(0)	2 (7.7)	0(0.0)	NA	NA	NA	NA	NA	NA	NA	NA	NA	0(0)
		R	1(3.8)	2(7.7)	22(84.5)	7(26.9)	NA	NA	NA	NA	NA	NA	NA	NA	NA	6(23.1)

spp=Species, NIT=Nitrofurantoin, CIP=Ciprofloxacin, SXT = Trimethoprim-sulfamethoxazole, GEN=Gentamycin, AMP=Ampicillin, AMC=Amoxy clavulanic acid, CXT=Cefotaxime, CRO=Ceftriaxone, CAZ=Ceftazidime, MRP=Meropenem, FEF=Cefepime, AK=Amikacin, TZP = Piperacillin Tazobactam, CL=clindamycin, NA=Not applicable, other bacteria spp=*Providencia* spp (2), and *Citrobacter* spp (1).

Multi-drug resistant bacterial strains causing UTI

MDR was observed in 35/106 (33.0%) isolated organisms. A high rate of MDR was observed in *S. aureus* 11 (31.4%), *E.coli* 7(20.0%), and *Klebsiella* spp (20.0%). A high MDR above 50% was observed in combination of Ampicillin, Gentamycin, and Trimethoprim-sulfamethoxazole (Table 5).

Table 5: Patterns of MDR bacteria among children under five years attending Magu District Hospital in Mwanza region, March to April 2023.

**Antimicrobial agent**	**n (%)**	***Acinetobacter* spp**	***E. coli***	***Enterobacter* spp**	***Klebsiella* spp**	***S. aureus***	***Pseudomonas aeruginosa***	***Proteus* spp**	***Morganella* spp**
AMP, GEN, SXT	18 (51.4)	2	3	1	3	8	0	0	1
AMP, GEN, CIP	5 (14.3)	0	2	0	0	3	0	0	0
AMP, CIP, NI, AMC	3 (8.6)	1	0	0	1	0	0	1	0
AMP, CIP, MERO	2 (5.7)	0	1	0	1	0	0	0	0
AMP, GEN, CIP, NI	2 (5.7)	1	0	0	1	0	0	0	0
TZP, GEN, CAZ	5 (14.3)	0	1	0	1	0	3	0	0
**MDR, n (%)**	35/106 (33.0)	4 (11.4)	7(20.0)	1 (2.9)	7 (20.0)	11 (31.4)	3 (8.6)	1 (2.9)	1 (2.9)

AMP=Ampicillin, GEN=Gentamycin, SXT= Trimethoprim-sulfamethoxazole, CIP= Ciprofloxacin, NI=Nitrofurantoin, MERO=Meropenem, TZP= Piperacillin Tazobactam, CAZ=Ceftazidime, AMC= Amoxy clavulanic acid.

The proportion of ESBL producers and MRSA strains

The MRSA was detected in 6/26 (23.1%) of the *S. aureus*. ESBL production was detected in 19/76 (25%) of gram-negative bacteria. Predominant ESBL-producing isolates were *E. coli, Klebsiella spp* and *Acinetobacter spp* spp (Figure 1).

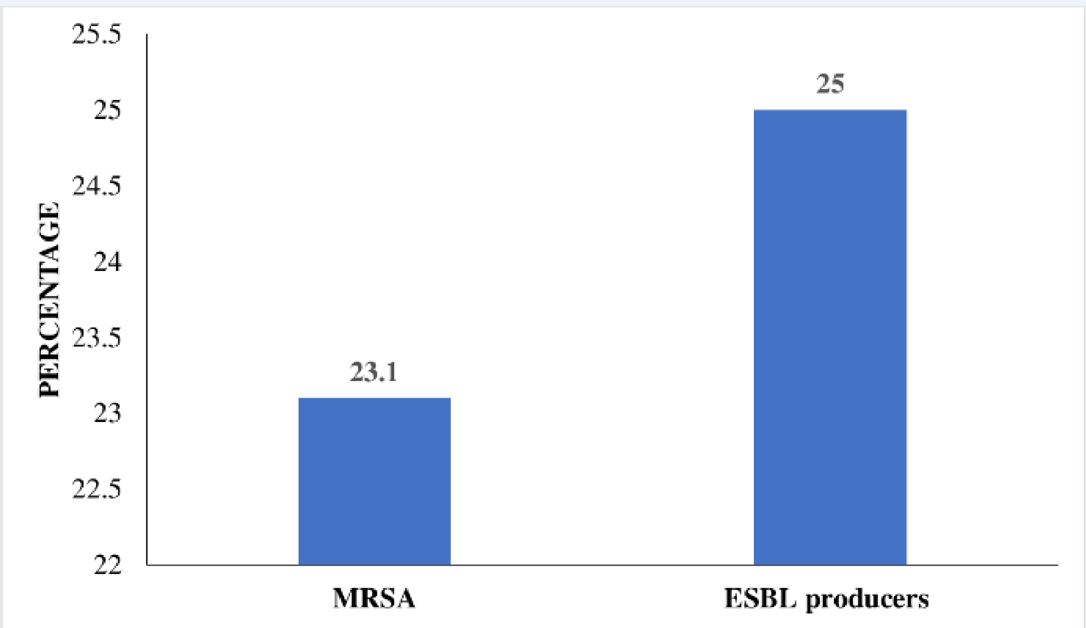

Fig 1: ESBL producers, and MRSA isolates recovered from the urine culture specimens of the febrile under-five children attending Magu District Hospital in Mwanza region, March to April 2023

Discussions

This study determined the proportion of UTI, bacterial aetiology, antimicrobial susceptibility patterns, and factors associated with UTI among under-five children. Our study found that 28.8% (95% CI -24.3-33.6) had culture-proven UTI. The proportion was lower than 39.7% reported by Festo et al. (2011) in a study conducted among under-fives in a consultant hospital in Mwanza, northwestern Tanzania (27), and 32.5% reported by Francine et al. in Cameroon (6). This study reported a higher proportion of UTI than in Kenya 11.9% (28), Mwanza Tanzania-16.8% (8), Bagamoyo-16.4% (4), Kilimanjaro-11.4% (23), and Nigeria 27.8% (16). Different risk factors can explain this variation between the current and previous studies among the population under the study, such as low hygienic standards and malnutrition (10). Additionally, only children with fever were included in this study, which may account for the higher prevalence than that conducted at Kilimanjaro, which included children with or without symptoms of UTI (23).

The common causative agent of UTI in under-five children was *E. coli*, followed by *Staphylococcus aureus*. Similar results were reported in previous studies conducted in Nigeria (2) and Cameroon (6). A study in Mwanza (8) on UTI in children also reported *E. coli* as the common isolate causing UTI. Another study at Muhimbili National Hospital, Dar es Salaam, Tanzania, reported *E. coli* as the common isolate, followed by *Klebsiella* spp, *and Staphylococcus aureus* (15). The bacterial spectrum obtained in this study is nearly identical to that conducted on the prevalence of UTI among children in Kilimanjaro, Tanzania, which identified *E. coli, Staphylococcus aureus, Klebsiella* spp, *Proteus*, and *Pseudomonas spp* as the common aetiology of UTI (23).

Most isolates were sensitive to nitrofurantoin, ciprofloxacin, meropenem, cefepime, and piperacillin tazobactam. These results agree with similar studies conducted in Mwanza (8), Kilimanjaro (23), Dar es Salaam in Tanzania (15), and Nigeria (1) on the susceptibility patterns. The high susceptibility of isolated bacteria to meropenem may be attributed to the fact that the injectable beta-lactam antibiotic is not frequently available in Tanzanian pharmacies. As a result, meropenem is less routinely administered in Tanzania than other more widely available antibiotics. Ciprofloxacin, on the other hand, is not commonly used in children under the age of 12 years. In addition, nitrofurantoin is not recommended for use in infants younger than six weeks instead, a first-generation cephalosporin such as cephalexin may be given until the infant reaches six weeks of age.

The current study shows that isolated bacteria were somewhat sensitive to ceftriaxone, amoxicillin-clavulanate, and ceftazidime, consistent with other studies conducted in Mwanza (8) and Dar es Salaam Tanzania (15), and Ethiopia (29). The findings suggest these commonly used antibiotics to treat various bacterial infections have variations of effectiveness against certain bacteria. The susceptibility may vary depending on the specific strain and resistance mechanisms, particularly in the case of extended-spectrum beta-lactamase (ESBL)--producing organisms.

The antibiotics frequently prescribed for empirical treatment, including trimethoprim-sulfamethoxazole, piperacillin, ampicillin, gentamycin, and penicillin demonstrated resistance. These findings agree with results obtained from previous studies conducted in Mwanza (8) and Bagamoyo in Tanzania (4), and Kenya (28). However, different findings were reported in the study conducted at Muhimbili National Hospital that most *E. coli* were sensitive to gentamycin (15). The disparity in resistance rates may be attributed to the fact that these antibiotics are

readily accessible at the primary healthcare level and are, therefore, being given empirically based on clinical features, accelerating antimicrobial resistance. The results of this study highlight the significance of performing a urine culture and an antimicrobial susceptibility test on febrile children.

In this study, the overall prevalence of ESBL-producing bacteria was comparatively lower than what has been reported in previous studies conducted in Tanzania, Mwanza (30) and Dar es Salaam (22). Furthermore, the study identified the most predominant ESBL-producing isolates as *E. coli*, followed by *Klebsiella spp* and *Acinetobacter spp*. This pattern is consistent with findings from other studies conducted in Ethiopia (18). The proportion of MRSA was also lower than in previous studies conducted in Nigeria (31) and India (32). Differences in geographical locations can explain lower proportions.

The current study found that children under 24 months had a higher risk of acquiring a UTI than those older than 24 months. Similar findings were reported by previous studies conducted in Mwanza (8) and Kilimanjaro in Tanzania (23). The finding may be explained by the fact that, children under the age of 24 months are at risk of acquiring UTI due to underdeveloped immune systems, wearing of nappies for children under two years, and urinary tract abnormalities (10).

This study found that the risk of urinary tract infections in non-circumcised children was high, consistent with previous studies conducted in Dhaka (10). Another study conducted by Cason DL et al found that circumcision can help prevent recurrent urinary tract infections in hospitalized infants. A study conducted in Indonesia reported that, circumcision was associated with a decreased incidence of UTI in children (33). Additionally, a study conducted in Ethiopia found that uncircumcised male children have a higher risk of acquiring UTI compared to those who are circumcised (13).

This study revealed that symptoms of vomiting, difficulty urination, body temperature greater or equal to 39 degrees centigrade, and abdominal pain were associated with UTI in febrile children under five years. These findings align with the previous studies conducted in Mwanza (8) and Nigeria (34,35). Similar study populations, risk factors, and environmental factors may have contributed to the consistency of these findings. This study also reported that a history of recurrent UTI, chronic disease, nutrition status, and duration of fever did not show a significant association with UTI in children under five years, which agrees with other reports from Nigeria (1), England (2), and Kilimanjaro-Tanzania (23).

Limitations of the study

We were unable to ascertain the prevalence of UTI and assess antimicrobial susceptibility patterns among children who had used antibiotics in the past two weeks due to challenges in recall bias.

Conclusions

We observed a relatively high proportion of under-five children with UTI, mainly due to *Escherichia coli* and *Staphylococcus aureus*. Several factors, including a history of vomiting, dysuria, nappy use, uncircumcision, and abdominal pain could independently predict UTI in children. The isolated organisms showed high resistance to commonly prescribed antibiotics for the treatment of UTI. This highlights the growing resistance to commonly used antibiotics and emphasizes the need for continuous epidemiologic surveillance in primary healthcare facilities in Tanzania. In addition, periodic reviews of empirical treatment for UTI should be done based on the prevailing antibiotic susceptibility patterns.

Acknowledgements

We want to convey our sincere gratitude to the Management of Sekou-Toure Regional Referral Hospital and Magu District Hospital for allowing us to conduct our research under their purview. We thank Mr. Castory Dome, Mr. Philipo Shokolo, and Mr. Leoncy Kapinga for their technical assistance and directives during data and specimen collection. We express our gratitude to our study respondents who willingly participated and cooperated fully, thus facilitating the successful completion of this study.

Author Contributions

Conceptualization: RE, NL, MM

Data Curation: RE

Formal Analysis: RE, MM

Investigation: RE, NL, MM

Methodology: RE, NL, MM

Supervision: NL, SY, AJ, MM

Validation: RE, NL, SY, AJ, MM

Visualization: RE, NL, SY, AJ, MM

Writing – Original Draft Preparation: RE, NL, MM

Writing – Review & Editing: RE, NL, SY, AJ, MM

Abbreviations

ATCC: American Type Culture Collection, CFU: Colony-Forming Unit, CLSI: Clinical and Laboratory Standards Institute, ESBL: Extended Spectrum Beta Lactamase, MDR: Multi-Drug Resistant, MRSA: Methicillin-Resistant *Staphylococcus Aureus*, UTI: Urinary Tract Infection, WHO: World Health Organization.

References

1. Ibeneme C, Oguonu T, Okafor H, Ikefuna A, Ozumba U. Urinary tract infection in febrile under five children in Enugu, South Eastern Nigeria. *Nigerian Journal of Clinical Practice*, 2014; 17(5), p.624. doi:https://doi.org/10.4103/1119-3077.141430.
2. Westwood ME, Whiting PF, Cooper J, Watt IS, Kleijnen J. Further investigation of confirmed urinary tract infection (UTI) in children under five years: a systematic review. *BMC Pediatrics*, 2005; 5(1). doi:https://doi.org/10.1186/1471-2431-5-2.
3. Brien KM, Stanton N, Edwards A, Hood K, Butler CC. Prevalence of urinary tract infection (UTI) in sequential acutely unwell children presenting in primary care: exploratory study. 2011;554268; doi:https://doi.org/10.3109/02813432.
4. Sangeda RZ, Paul F, Mtweve DM. Prevalence of urinary tract infections and antibiogram of uropathogens isolated from children under five attending Bagamoyo District Hospital in Tanzania: A cross-sectional study. *F1000Research*, 2021;10, p.449. doi:https://doi.org/10.12688/f1000research.52652.1
5. Alex Fortunatus, Magufwa. Epidemiology of Urinary Tract Infection among febrile Children under five years in morogoro municipality, Tanzania. 2016.
6. Kouemo F, Guy Pascal Ngaba, Christiane D, Pradel L, Cécile Okalla Ebongue, Désiré Dieudonné Adiogo. Etiologic Profile and Sensitivity Pattern of Germs Responsible for Urinary Tract Infection Among Under-five Children in Douala, Cameroon: A Hospital-Based Study. *Avicenna Journal of Clinical Microbiology and Infection*, 2019;6(2), 10;pp.49–56. doi:https://doi.org/10.34172/ajcmi.
7. Dm K, Mosha F, Moyo S, Mi M. Etiology and Antimicrobial Susceptibility Patterns of Bacterial Agents Causing Urinary Tract Infection in Children under Five years , dar es Salaam . 2020;2(1).
8. Msaki BP, Mshana SE, Hokororo A, Mazigo HD, Morona D. Prevalence and predictors of urinary tract infection and severe malaria among febrile children attending Makongoro health centre in Mwanza city, North-Western Tanzania. *Archives of Public Health*, 2012;70(1). doi:https://doi.org/10.1186/0778-7367-70-4
9. Renko M, Salo J, Ekstrand M, Pokka T, Pieviläinen O, Uhari M *et al*. Meta-analysis of the Risk Factors for Urinary Tract Infection in Children. *Pediatric Infectious Disease Journal*, 41(10), pp.787–792. doi:https://doi.org/10.1097/inf.0000000000003628
10. Ferdaus J, Moinuddin G, Islam MT, Rashid MHO, Shawly MR, Alim A. Risk Factors of urinary tract infection in preschool children in Dhaka, Bangladesh. *Bangladesh Medical Journal*, 2017;45(3), pp.134–137. doi:https://doi.org/10.3329/bmj.v45i3.3312

11. Mohamed F. Prevalence and factors associated with urinary tract infection among febrile children admitted at Dodoma regional referral hospital. 2019.
12. Shaikh N, Morone NE, Bost JE, Farrell MH. Prevalence of urinary tract infection in childhood: a meta-analysis. *The Pediatric infectious disease journal*, [online] 2008;27(4), pp.302–8. doi:https://doi.org/10.1097/INF.0b013e31815e412,
13. Mekonnen S, Tesfa T, Tadesse Shume, Fikru Tebeje, Kedir Urgesa, Fitsum Weldegebreal. Bacterial profile, their antibiotic susceptibility pattern, and associated factors of urinary tract infections in children at Hiwot Fana Specialized University Hospital, Eastern Ethiopia. *PLOS ONE*, 2023;18(4), pp.e0283637–e0283637. doi:https://doi.org/10.1371/journal.pone.0283637.
14. Byarugaba DK. Antimicrobial resistance in developing countries and responsible risk factors. *International Journal of Antimicrobial Agents*, 2004;24(2), pp.105–110. doi:https://doi.org/10.1016/j.ijantimicag.
15. Fredrick F, Joel Msafiri Francis, Maulidi Fataki, Maselle SY. Aetiology, antimicrobial susceptibility and predictors of urinary tract infection among febrile under-fives at Muhimbili National Hospital, Dar es Salaam-Tanzania. *African Journal of Microbiology Research*,2013; 7(12), pp.1029–1034. doi:https://doi.org/10.5897/ajmr12.1866
16. Jiya N M, FWACP, Ibitoye PK, FWACP, Jiya F B M. Urinary tract infection and malaria co-morbidity in febrile children with sickle-cell anaemia in Sokoto, Nigeria 2012;20(1):299–305.
17. Said Aboud, Robinson Mdegela. Situation analysis and recommendations on Antibiotic Use and Resistance in Tanzania 2015;(March):1–83.
18. Tola MA, Abera NA, Gebeyehu YM, Dinku SF, Tullu KD. High prevalence of extended-spectrum beta-lactamase-producing Escherichia coli and Klebsiella pneumoniae fecal carriage among children under five years in Addis Ababa, Ethiopia. *PLOS ONE*, (2021), 16(10), p.e0258117. doi:https://doi.org/10.1371/journal.pone.0258117.
19. Nuru Letara, James Samwel Ngocho, Karami N, Msuya E, Balthazar Nyombi, Kassam, NA *et al*. Prevalence and patient related factors associated with Extended-Spectrum Beta-Lactamase producing Escherichia coli and Klebsiella pneumoniae carriage and infection among pediatric patients in Tanzania. *Scientific Reports*, 2021; 11(1). doi:https://doi.org/10.1038/s41598-021-02186-2
20. Onduru OG, Mkakosya RS, Rumisha SF, Aboud S. Carriage Prevalence of Extended-Spectrum β-Lactamase Producing Enterobacterales in Outpatients Attending Community Health Centers in Blantyre, Malawi. *Tropical Medicine and Infectious Disease*, 2021; 6(4) p.179. doi:https://doi.org/10.3390/tropicalmed6040179
21. Birgy A, Cohen R, Levy C, Bidet P, Courroux C, Benani M, *et al*. Community faecal carriage of extended-spectrum beta-lactamase-producing Enterobacteriaceaein french children. *BMC Infectious Diseases*, 2012;12(1). doi:https://doi.org/10.1186/1471-2334-12-315.
22. Tellevik MG, Blomberg B, Kommedal Ø, Maselle SY, Langeland N, Moyo S.J. High Prevalence of Faecal Carriage of ESBL-Producing Enterobacteriaceae among Children in Dar es Salaam, Tanzania. *PLOS ONE*, 2016;11(12), p.e0168024. doi:https://doi.org/10.1371/journal.pone.0168024
23. Gidabayda J, Philemon R, S Abdallah M, M Saajan A, Temu T, Kunjumu *et al*. Prevalence, Aetiology, and Antimicrobial Susceptibility Patterns of Urinary Tract

Infection Amongst Children Admitted at Kilimanjaro Christian Medical Centre, Moshi, Tanzania. *East African Health Research Journal*, 2017;1(1), pp.53–61. doi:https://doi.org/10.24248/eahrj.v1i1.388

24. Assessment on how to Calculate Weight-for-Height Z-Score (WHZ) in C hildren 0 − 59 Months of Age. :6–9

25. CLSI. Performance Standards for Antimicrobial Susceptibility Testing. M100, 30th ed. 2022; 40(1).

26. Abdelmoktader A, Talal A, Far E. Methods of ESBLs Detection in Clinical Microbiology Lab. 2019;1–8.

27. Festo E, Kidenya BR, Aldofina Hokororo, Mshana. Predictors of Urinary tract infection among febrile children attending at Bugando Medical Centre Northwestern, Tanzania. *Archives of Clinical Microbiology*, 2011;2(5).

28. Masika WG, Meara WP, Holland TL, Armstrong. Contribution of urinary tract infection to the burden of febrile illnesses in young children in rural Kenya. *PLOS ONE*, 2017;12(3), p.e0174199. doi:https://doi.org/10.1371/journal.pone.0174199

29. Marami D, Balakrishnan S, Seyoum B. Prevalence, Antimicrobial Susceptibility Pattern of Bacterial Isolates, and Associated Factors of Urinary Tract Infections among HIV-Positive Patients at Hiwot Fana Specialized University Hospital, Eastern Ethiopia. *Canadian Journal of Infectious Diseases and Medical Microbiology*, 2019, pp.1–8. doi:https://doi.org/10.1155/2019/6780354

30. Ahmed M, Moremi N, Mirambo MM, Hokororo A, Mushi MF, Seni J *et al*. Multi-resistant gram negative enteric bacteria causing urinary tract infection among malnourished underfives admitted at a tertiary hospital, northwestern, Tanzania. *Italian Journal of Pediatrics*, 2015;41(1). doi:https://doi.org/10.1186/s13052-015-0151-5

31. Abubakar U, Sulaiman SAS. Prevalence, trend and antimicrobial susceptibility of Methicillin Resistant Staphylococcus aureus in Nigeria: a systematic review. *Journal of Infection and Public Health*, 2018;pp.763–770. doi:https://doi.org/10.1016/j.jiph.

32. Arora S, Devi P, Arora U, Devi B. Prevalence of Methicillin-resistant Staphylococcus Aureus (MRSA) in a Tertiary Care Hospital in Northern India. *Journal of Laboratory Physicians*, 2010;2(02), pp.078–081. doi:https://doi.org/10.4103/0974-2727.72154.

33. Irfan Wahyudi, Putu Angga Risky Raharja, Gerhard Reinaldi Situmorang and Arry Rodjani. Circumcision reduces urinary tract infection in children with antenatal hydronephrosis: Systematic review and meta-analysis. *Journal of Pediatric Urology*, 2023;19(1), pp.66–74. doi:https://doi.org/10.1016/j.jpurol.2022.10.029

34. Okunola P, Ibadin M, Ofovwe G. Predicting Urinary Tract Infection in Malaria Co-morbidity among Under Fives. *Nigerian Hospital Practice*, 2011;7(3-4). doi:https://doi.org/10.4314/nhp.v7i3-4.67124

35. Estefanía J, Regalado A, Brito MO, Enrique L. Prevalence of urinary tract infection and associated factors in hospitalized patients aged 0 to 5 years . 2021;22(1):1–8.

POINT TO POINT RESPONSE TO EDITOR AND REVIEWERS' COMMENTS

Editor's comments
When submitting your revision, we need you to address these additional requirements.
1. Please ensure that your manuscript meets PLOS ONE's style requirements, including those for file naming. The PLOS ONE style templates can be found at https://journals.plos.org/plosone/s/file?id=wjVg/PLOSOne_formatting_sample_main_body.pdf and https://journals.plos.org/plosone/s/file?id=ba62/PLOSOne_formatting_sample_title_authors_affiliations.pdf

Response: We have tried our best to write the manuscript following PLOS ONE's style

2. We suggest you thoroughly copyedit your manuscript for language usage, spelling, and grammar. If you do not know anyone who can help you do this, you may wish to consider employing a professional scientific editing service. Whilst you may use any professional scientific editing service of your choice, PLOS has partnered with both American Journal Experts (AJE) and Editage to provide discounted services to PLOS authors. Both organizations have experience helping authors meet PLOS guidelines and can provide language editing, translation, manuscript formatting, and figure formatting to ensure your manuscript meets our submission guidelines. To take advantage of our partnership with AJE, visit the AJE website (http://learn.aje.com/plos/) for a 15% discount off AJE services. To take advantage of our partnership with Editage, visit the Editage website (www.editage.com) and enter referral code PLOSEDIT for a 15% discount off Editage services. If the PLOS editorial team finds any language issues in text that either AJE or Editage has edited, the service provider will re-edit the text for free. Upon resubmission, please provide the following: The name of the colleague or the details of the professional service that edited your manuscript. A copy of your manuscript showing your changes by either highlighting them or using track changes (uploaded as a *supporting information* file). A clean copy of the edited manuscript (uploaded as the new *manuscript* file)"

Response: The manuscript has been thoroughly checked for language usage, spelling and grammar using Grammarly Premium

3. In your Data Availability statement, you have not specified where the minimal data set underlying the results described in your manuscript can be found. PLOS defines a study's minimal data set as the underlying data used to reach the conclusions drawn in the manuscript and any additional data required to replicate the reported study findings in their entirety. All PLOS journals require that the minimal data set be made fully available. For more information about our data policy, please see http://journals.plos.org/plosone/s/data-availability. Upon re-submitting your revised manuscript, please upload your study's minimal underlying data set as either Supporting Information files or to a stable, public repository and include the relevant URLs, DOIs, or accession numbers within your revised cover letter. For a list of acceptable

repositories, please see http://journals.plos.org/plosone/s/data-availability#loc-recommended-repositories. Any potentially identifying patient information must be fully anonymized. Important: If there are ethical or legal restrictions to sharing your data publicly, please explain these restrictions in detail. Please see our guidelines for more information on what we consider unacceptable restrictions to publicly sharing data: http://journals.plos.org/plosone/s/data-availability#loc-unacceptable-data-access-restrictions. Note that it is not acceptable for the authors to be the sole named individuals responsible for ensuring data access. We will update your Data Availability statement to reflect the information you provide in your cover letter.

Response: The study's minimal underlying data set has been uploaded as a Supporting Information files

4. We note that you have indicated that data from this study are available upon request. PLOS only allows data to be available upon request if there are legal or ethical restrictions on sharing data publicly. For more information on unacceptable data access restrictions, please see http://journals.plos.org/plosone/s/data-availability#loc-unacceptable-data-access-restrictions.
In your revised cover letter, please address the following prompts:
a) If there are ethical or legal restrictions on sharing a de-identified data set, please explain them in detail (e.g., data contain potentially sensitive information, data are owned by a third-party organization, etc.) and who has imposed them (e.g., an ethics committee). Please also provide contact information for a data access committee, ethics committee, or other institutional body to which data requests may be sent.
b) If there are no restrictions, please upload the minimal anonymized data set necessary to replicate your study findings as either Supporting Information files or to a stable, public repository and provide us with the relevant URLs, DOIs, or accession numbers. For a list of acceptable repositories, please see http://journals.plos.org/plosone/s/data-availability#loc-recommended-repositories.
We will update your Data Availability statement on your behalf to reflect the information you provide.
Response: The study's minimal underlying data set has been uploaded as a Supporting Information files

5. Please include your full ethics statement in the 'Methods' section of your manuscript file. In your statement, please include the full name of the IRB or ethics committee who approved or waived your study, as well as whether or not you obtained informed written or verbal consent. If consent was waived for your study, please include this information in your statement as well.

Response: The study obtained ethical clearance number MUHAS-REC-12-2022-1484 from the Muhimbili University of Health and Allied Sciences Senate Research and Publication Committee. This has been addressed in lines 228 to 230.

Comments from reviewer 1

1.I am not sure how UTI often leads to septicemia, dehydration. Sometimes from kidney infection bacteria may have chance to enter blood stream and cause bacteremia. Line #47-48

“Urinary tract infection (UTI) is common in under-five children presenting with fever with significant consequences leading to septicemia, dehydration, kidney scarring”

Response: The sentence has been revised in line 55 of the introduction section.

2. I suggest authors to describe the source and study population including Eligibility criteria for selection of the study participants. In this study it was mentioned that under five children with fever who visited the health care setting. Fever can indicate server condition (Acute febrile illness). Were there any additional criteria to select? What other laboratory tests were performed? Were infant included?

Response: We involved children aged 2 months and above who met the inclusion criteria. We have to revise and write: We recruited children with fever who the attending clinician suspected to have UTI based on signs and symptoms such as fever of 38°C or above, pain or burning when urinating, frequency urination, discoloured urine, lower abdominal pain, or vomiting.. The eligibility criteria have been added; line 106 to 111

3. Was there multiple infections?

Response: No multiple infection was obtained with significant growth of bacteria counts of $\geq 10^5$ CFU/ml. We have rephrased the result section to reflect this finding line 275 to 276.

4. The description of AST in the manuscript may need revision example ‘highly susceptible’ as far as I know interpretation of AST result is Resistant, Intermediate or susceptible based on cut point. “E. *coli* were highly susceptible to cefepime”

Response: This has been addressed in table 4 of the manuscript, interpretation of AST which includes resistant, intermediate and sensitive were added in the table.

5. Was the tool (questionnaire) used in the study validate? Briefly describe

Response: Yes, the questionnaire used for data collection was validated, and this is explained in the data collection section, line 124.

6. It was indicated that the urine specimens were stored at 4°C until they tested. For how long were they stored? How far regional lab is from the hospital where study participants were recruited. The integrity of the specimen is key for recovery of bacteria.

Response: Samples were stored in the refrigerator for not more than 12 hours; we have indicated this in the sample collection section line 141.

7. Describe how antibiotics used in the study were selected

Response: Descriptions on how antibiotics used in the study were selected has been added in line 174 to 175**.**

8. Did authors use only CLED for identification of Gram-positive and Gram-negative bacteria? How S. aureus and Micrococcus was differentiated? Overall, the types of culture media used for AST were not indicated.

Response: We differentiated Staphylococcus aureus from Micrococcus species by observing colony characteristics and performing a gram stain. The type of culture used for AST has been indicated in lines 175-177

9. As the definition of multi-drug resistance may vary it advisable to include operational definition for multi-drug resistance.
Response: We have added in the materials and methods a subsection of the definition of terms. The definition of multi-drug resistance has been included in lines 143-153

10. The sample size for was not indicted in the main method section.
Response: The sample has been indicated in methodology line 114

11. Consider including 95% CI for culture confirmed UTI.
Response: 95% CI has been added to the culture confirmed UTI in the abstract, results and discussion sections

12. Figure 1, Y-axis is not labelled. Also include frequency and % on the top of each bar
Response: Figure one was changed to a table 2 which indicates frequency and percentage

13. It is difficult to understand the results presented in Table 3. Consider including R, I, and S
Response: Table 3 has been revised for clarity and changed to table 4 since we added another table

Minor comments
1. Line# 135 "…..Identify…..' the first letter should not be capital
2. 'Gram-positive' and 'gram-positive' is interchangeably used. It is good to stick to one. 'Gram' or 'gram'
Response: The first letter of "identify" has been written in lowercase. "Gram positive" and "gram positive" have been revised to "gram positive." However, when starting a sentence, "Gram" is written with a capital letter

Comments from Review 2
Comments for Reviewer #2 were on the sticky note on the PDF file. We have addressed all comments and modified the manuscripts
In title you said etiological profile, is it include fungi?
Response: The title has been revised to reflect the bacterial etiology; line 1

Abstrct background:Where UTI is common infection?. Avoid use of short form in abstract. Better to be consistence as per title or should modify the title accordingly. Foristance febrile underfive,antibiotic vs antimicrobial,proportion of UTI vs etiology profile
Response: We rephrased the sentences for clarity and to ensure consistency, and short-form words were omitted; lines 17-23

Abstract methodology: Why only febrile? Is fever the only criteria to be suspected as UTI for under five? Operationalize this. The method is not written well, rewrite again

Response: The materials and methods subsection in the abstract has been rewritten for clarity; Lines 25-32

Abstract results : rewrite the result very well. Like of all 28.8% had Lab, confirmed means? do you mean culture confirmed? write the organism name in italic. Is circumcision a common practice for both male and female or for only for male? is those for female taken as nor not applicable? Rewrite bacteria in italic. Is suceptibility pattern for overall? or *E.. coli* & *S. aurues*

Response: The result subsection of the abstract has been rewritten for clarity. Bacteria have been written in italics; the susceptibility patterns are for *E.coli* and *S. aureus*; Lines 34-43

Introduction

Add One paragraph for common cause of UTI from different literature.

Response: A paragraph detailing the common causes of UTI has been included; Line 62-69

what does the renal functions of the kidney mean?

Response: The sentence has been revised to reflex that, Early diagnosis is essential to preserve and maintain the proper functioning of the kidneys and prevent any further decline in their function. Line 56-57

uncircumcision in male?

Response: Yes uncircumcission in male, The sentence has been paraphrased; Line 43&73

The prevalence of UTI among under-five children ranges from 7.4 % to 43.6% where this studies conducted

Response: The reported prevalence was indicated from Nigeria and Tanzania; Line 59-60

The risk factors for acquiring UTI in under-five children are multiple and differ with geographical locations, settings, and seasons. Add reference

Response: Reference number 9 has been added; Line 71

Antimicrobial resistance (AMR) has become significant concern worldwide.add reference

Response: Reference number 14 was added; Line 77

Can you generalize with your specific hospital data about the treatment? or can leads you to change treatment guidelines?

Response: The sentence has been ommited, since the same information were discussed in line 89-94

Materials and methods

Line 91, Can you clarify on primary health care and district hospital? in your title it says primary health care whereas the study is conducted in district hospital? Besides this your study area description don't indicate as whether it is urban or rural but in reasoning or gab analysis your said that rural community lacks attention?
Response: Yes, in Tanzania, a district hospital is considered a primary healthcare facility. The Magu district, where our research was conducted, is situated in the rural parts of the region. However, when classifying the demographic data, we categorized urban as individuals from the town area of Magu, and rural as those from other areas outside the town; Line 103

why only children with fever?
Response: Fever is a common symptom of various illnesses, the aim of recruiting children with fever ensured that,study participants have a higher likelihood of having the targeted condition; Line 106-109

The total sample size should be indicated here.
Response: The total sample size was indicated; Line 114

In Data collection your sociodemographic is limited why?
Response: The scope and objectives of this study prioritized these sociodemographic variables; Line 121

Is Sekou Toure Regional Referral Hospital different from above hospital?
Response: Yes, the study was conducted at Magu District Hospital. However, the samples were analyzed at Sekou Toure Regional Referral Hospital since the District hospital lacked the capacity to process culture samples

Line 131- 132, write more how do you handle in case of mixed growth
Response: Handling of mixed growth of bacteria has been indicated; Line 161-162

Line 135, Identify should start with small letter
Response: The word Identify written starting with small letter Line 165

is this the only biochemical test? how do you identify like *P. aerigenosa* & *Acitenobacter* spp
Response: Oxidase test used to identify *P. aerigenosa* & *Acitenobacter* spp has been added Line 168-169

Antimicrobial susceptibility testing: Can we report erthromycin for organisms from UTI? this shows you haven't followed the CLSI properly.
Response: Erthromycin disk has been removed from this manuscript

Extended-Spectrum Beta-Lactamase Detection: Is this for the three or for one? if for one you have write cefotaxime or cftriaxone or ceftazidime. is the increase of zone of inhibition towards CAZ or CXT or both?

Response: The sentence has been rephrased Line 192-193, 199-201

Quality assurance: As indicated in the quideline, on which guidelines?
Response: The sentence" as indicated in the quideline was ommited since checking expiration date and assess reagents does not need any guideline.

Data analysis: ≤ 0.2, this cut of value most of the time is 0.25. Do you have some justification for it?
Response: The choice of a cut-off value in data analysis can depend on the specific field of study, the nature of the data, and the research question being addressed, This cut-off value allows for broader inclusion of variables that may have some potential association with the outcome of interest. Line 223

Ethical approve: include ethical number
Response: Ethical clearance number MUHAS-REC-12-2022-1484 has been added Page 228

Results:
Socio-demographic and clinical characteristics: so you you justify as this study is conducted in rural area: No need of repeating 368
Response: The study was conducted in Magu District, which is primarily a rural area. However, during the recruitment of participants, some individuals residing in the town were included, categorizing them as urban participants. Additionally, participants from villages near Magu town were also included, classifying them as rural area participants. The sentence has been paraphrased. Line 241.

The proportion of UTI among under-five children: why you classify the age only in two category? do you have justification?
Response: Dividing age into two categories was made in order to compare the usage of nappies between two distinct age groups: children under 24 months and those above 24 months. Line 247-248

I think the use of p-value is not important here as the title is the proportion of UTI
Response: The p-value is used in proportion tests to assess the statistical significance of the observed sample proportion compared to a hypothesized population proportion. It helps determine whether the difference between the observed proportion and the hypothesized proportion is statistically significant or simply due to random chance Line 248

what does it means 0-2 days? is there a zero day?
Response: The sentence has been rewritten as ≤2 days; Line 251

is there a means that you know a child of five days have abdominal pain or not? similarly for others
Response: The doctor reported this after obtaining the child's clinical history from the parents or guardian and conducting a thorough physical examination; Line 244

Put p in italic.

Response: P value has been written in italic Line 247-260

is UTI only expected only at high temp what about lower temp?

Response: According to the World Health Organization (WHO), a body temperature of 38 degrees Celsius is generally considered a threshold for fever in both adults and children. Therefore, our studies specifically recruited children under the age of five with a fever of 38 degrees Celsius and above. Line 258-259

The line with overall is not correct. put it at the end of the table and the table header should have a line on the top and bottom. why only positive? remove p-value from here? is the p-value from chi-square of logistic regression?. 0.2 should be written 0-2. Axillary temperature of ≥39 should be written well. Place of residence, why it comes last? why not on socio-demographic? font style and size should be similar

Response: The line with overall proportion has been removed ,The line was added on top and bottom of table header, Negative n (%) column has been added in the table. P value used is from chi square and it tells as if the difference in proportion is significant or not significant. Duration of fever 0.2 was written well as <2; An axillary temperature of ≥39 was written well; The place of residence variable was shifted to demographic factors; Font size was setted Table 1

Modify the title "Bacteria causing UTI among under-five children". you can *staphylococcus aureus* write in short form once you write in long and short form then after.

Response: The title has been modified to" Bacterial uropathogens isolated from Urinary Tract Infection positive under five children. *Staphylococcus aureus* has been written in short form.

Figure : think over febrile children. modify the organism name on the figure. remove the key from main figure and indicate each organism separately. Give title for x-and y-axis. Indicate number and % on bar graph.

Response: The figure was presented in Table including frequency and per cent of all isolates, Table 2

Factors associated with UTI: This sections seems more of discussion. try to write it properly again. what is CPR, and APR? is this common? I know COR & AOR? where candidate or associated? include OR,95%CI & P-value for each cells which is candidate.

Response: CPR =Crude Prevalence Ratio, APR=Adjusted Prevalence Ratio. These are used when the prevalence of the outcome is greater than 15. Our prevalence in this study was 28.8; hence, we used CPR and APR instead of COR&AOR. CPR and APR have been included. Lines 343 - 349

Table 2: Characteristics should be variables, Duration of fever, 0.2 should be 0-2, why uppercase for some variable? check for all. Male circumcision, how do handle for those female here?

Response: Characteristics have been changed to variable, Duration of fever 0.2 changed to ≤2, Upper case was changed to lower case in YES and NO. Females were excluded from the data as analysis was done. Table 3

write uniformly for all antibiotics you write in lower case for some and uppercase here
Response: Antibiotic was written in lowercase; Line 312

Tabel 3: consistence issue. when we think susceptibility we have to think about Sensitive, Intermediate and Resistance both your table doesn't indicate this one. S.a not scientific similarly for the others. Key, this should be out of table and as keynote:
Response: The table title has been revised as sesntivity pattern for consistency. The pathogens names has been written in scientific form and the Key shifted out of the table; Table 4

MDR, ESBL and MRSA Should be defined in your method
Response: Defination of MDR, ESBL and MRSA has been added in the methods new susbsection of definition of terms line 143-153

Table 4, your title lucks consistency. I think MRSA and the like is should not be presented in one table. present with other way like pie chart. MDR, This should be presented separately & should be shown how it comes.
Response: MRSA and ESBL producers bacteria have been presented in a histogram in Figure 1 and MDR has been presented separately in Table 5

Discussion
I think it better you start with the strength and weakness of the study. rather than summarization of your finding. Your discussion poorly organized doesn't discus about the most important part of the study, ESBL, MRSA & MDR. Abserve consistency
Response: Discussion has been revised

Better show the number for comparison and put your study in CI. Similarly for the others include. Is this the only reason? what about nitrofurantoin?
Response: The number for comparisons has been added, and CI has been added in Line 343 to 349. Nitrofurantoin has been included in the discussion, Line 371 to 373.

"High resistance rates were observed to antibiotics commonly used in rural settings", what does it mean? Provide reference
Response: The sentence has been rewritten as 'The antibiotics frequently prescribed for empirical treatment Line 381

Do you have scientific background or reference for this? " as this study was conducted in rural areas where UTI is more likely to be community-acquired, whereas, in urban areas, UTI is more likely to be acquired at the hospital from possibly admitted patients.

Response: The sentence has been removed as there is no proper reference to support its validity

put reference at the end of the sentence?
Response: The reference has been moved to the end of the sentence Line 384 and 400

Indicate which age group? Wearing of nappies, you assessed for all age?
Response: Wearing nappies werer assessed in children under 24 months; Line 402

you have to discuss circumcision separately and give emphasis even if your study subjects is both male and females. compare and contrast with other finding.
Response: Circumcision has been discussed in separate paragraph; Line 404-410

what does it mean? is this limitation of your study?if yes then write in the limitation section
Response: Limitation section has been added; Line 419-422

Conclusion and recommendation: Write as conclusion or separately. following the journal guideline. Not clear it is miss-leading; what does it mean? why only on AMR? looking at your title
Response: The section conclusion and recommmednation has been written as conclusion and content revised for clarity; Lines 425-432

Acknowledgements: we should start with capital letter. Who do you refer in “gratitude to respondents who willingly participated”?
We’ has been wrritten starting with capital letter and the sentence has been rephrased for clarity; Lines 438-439

Reference: follow the journal guideline for referencing
References has been reviewed as per journal guideline; Line 460-577
