# Supplementary material for: Bacterial aetiology, antimicrobial susceptibility patterns, and factors associated with urinary tract infection among under-five children at primary health facility, North-Western Tanzania
Source: PLoS One. 2024 May 6;19(5):e0303369. doi: 10.1371/journal.pone.0303369 (PMC11073726; doi:10.1371/journal.pone.0303369)
Supplement: Attachment — Submitted filename: Response to Reviewers.docx [file pone.0303369.s003.docx]

**POINT TO POINT RESPONSE TO EDITOR AND REVIEWERS’ COMMENTS**

**Editor’s comments**

When submitting your revision, we need you to address these additional requirements.

1. Please ensure that your manuscript meets PLOS ONE's style requirements, including those for file naming. The PLOS ONE style templates can be found at <https://journals.plos.org/plosone/s/file?id=wjVg/PLOSOne_formatting_sample_main_body.pdf> and <https://journals.plos.org/plosone/s/file?id=ba62/PLOSOne_formatting_sample_title_authors_affiliations.pdf>

**Response:** We have tried our best to write the manuscript following PLOS ONE’s style

2. We suggest you thoroughly copyedit your manuscript for language usage, spelling, and grammar. If you do not know anyone who can help you do this, you may wish to consider employing a professional scientific editing service. Whilst you may use any professional scientific editing service of your choice, PLOS has partnered with both American Journal Experts (AJE) and Editage to provide discounted services to PLOS authors. Both organizations have experience helping authors meet PLOS guidelines and can provide language editing, translation, manuscript formatting, and figure formatting to ensure your manuscript meets our submission guidelines. To take advantage of our partnership with AJE, visit the AJE website (<http://learn.aje.com/plos/>) for a 15% discount off AJE services. To take advantage of our partnership with Editage, visit the Editage website ([www.editage.com](http://www.editage.com)) and enter referral code PLOSEDIT for a 15% discount off Editage services.  If the PLOS editorial team finds any language issues in text that either AJE or Editage has edited, the service provider will re-edit the text for free. Upon resubmission, please provide the following: The name of the colleague or the details of the professional service that edited your manuscript. A copy of your manuscript showing your changes by either highlighting them or using track changes (uploaded as a *supporting information* file). A clean copy of the edited manuscript (uploaded as the new *manuscript* file)”

**Response:** The manuscript has been thoroughly checked for language usage, spelling and grammar using Grammarly Premium

3. In your Data Availability statement, you have not specified where the minimal data set underlying the results described in your manuscript can be found. PLOS defines a study's minimal data set as the underlying data used to reach the conclusions drawn in the manuscript and any additional data required to replicate the reported study findings in their entirety. All PLOS journals require that the minimal data set be made fully available. For more information about our data policy, please see <http://journals.plos.org/plosone/s/data-availability>. Upon re-submitting your revised manuscript, please upload your study’s minimal underlying data set as either Supporting Information files or to a stable, public repository and include the relevant URLs, DOIs, or accession numbers within your revised cover letter. For a list of acceptable repositories, please see <http://journals.plos.org/plosone/s/data-availability#loc-recommended-repositories>. Any potentially identifying patient information must be fully anonymized. Important: If there are ethical or legal restrictions to sharing your data publicly, please explain these restrictions in detail. Please see our guidelines for more information on what we consider unacceptable restrictions to publicly sharing data: <http://journals.plos.org/plosone/s/data-availability#loc-unacceptable-data-access-restrictions>. Note that it is not acceptable for the authors to be the sole named individuals responsible for ensuring data access. We will update your Data Availability statement to reflect the information you provide in your cover letter.

**Response:** The study’s minimal underlying data set has been uploaded as a Supporting Information files

4. We note that you have indicated that data from this study are available upon request. PLOS only allows data to be available upon request if there are legal or ethical restrictions on sharing data publicly. For more information on unacceptable data access restrictions, please see <http://journals.plos.org/plosone/s/data-availability#loc-unacceptable-data-access-restrictions>. In your revised cover letter, please address the following prompts:

a) If there are ethical or legal restrictions on sharing a de-identified data set, please explain them in detail (e.g., data contain potentially sensitive information, data are owned by a third-party organization, etc.) and who has imposed them (e.g., an ethics committee). Please also provide contact information for a data access committee, ethics committee, or other institutional body to which data requests may be sent.

b) If there are no restrictions, please upload the minimal anonymized data set necessary to replicate your study findings as either Supporting Information files or to a stable, public repository and provide us with the relevant URLs, DOIs, or accession numbers. For a list of acceptable repositories, please see <http://journals.plos.org/plosone/s/data-availability#loc-recommended-repositories>.

We will update your Data Availability statement on your behalf to reflect the information you provide.

**Response:** The study’s minimal underlying data set has been uploaded as a Supporting Information files

5. Please include your full ethics statement in the ‘Methods’ section of your manuscript file. In your statement, please include the full name of the IRB or ethics committee who approved or waived your study, as well as whether or not you obtained informed written or verbal consent. If consent was waived for your study, please include this information in your statement as well.

**Response:** The study obtained ethical clearance number MUHAS-REC-12-2022-1484 from the Muhimbili University of Health and Allied Sciences Senate Research and Publication Committee. This has been addressed in lines 228 to 230.

**Comments from reviewer 1**

1.I am not sure how UTI often leads to septicemia, dehydration. Sometimes from kidney infection bacteria may have chance to enter blood stream and cause bacteremia. Line #47-48 “Urinary tract infection (UTI) is common in under-five children presenting with fever with significant consequences leading to septicemia, dehydration, kidney scarring”

**Response**: The sentence has been revised in line 55 of the introduction section.

2. I suggest authors to describe the source and study population including Eligibility criteria for selection of the study participants. In this study it was mentioned that under five children with fever who visited the health care setting. Fever can indicate server condition (Acute febrile illness). Were there any additional criteria to select? What other laboratory tests were performed? Were infant included?

**Response:** We involved children aged 2 months and above who met the inclusion criteria. We have to revise and write: We recruited children with fever who the attending clinician suspected to have UTI based on signs and symptoms such as fever of 38^o^C or above, pain or burning when urinating, frequency urination, discoloured urine, lower abdominal pain, or vomiting.. The eligibility criteria have been added; line 106 to 111

3. Was there multiple infections?

**Response:** No multiple infection was obtained with significant growth of bacteria counts of ≥10⁵ CFU/ml. We have rephrased the result section to reflect this finding line 275 to 276.

4. The description of AST in the manuscript may need revision example ‘highly susceptible’ as far as I know interpretation of AST result is Resistant, Intermediate or susceptible based on cut point. “E*. coli* were highly susceptible to cefepime”

**Response:** This has been addressed in table 4 of the manuscript, interpretation of AST which includes resistant, intermediate and sensitive were added in the table.

5. Was the tool (questionnaire) used in the study validate? Briefly describe

**Response:** Yes, the questionnaire used for data collection was validated, and this is explained in the data collection section, line 124.

6. It was indicated that the urine specimens were stored at 4^o^C until they tested. For how long were they stored? How far regional lab is from the hospital where study participants were recruited. The integrity of the specimen is key for recovery of bacteria.

**Response:** Samples were stored in the refrigerator for not more than 12 hours; we have indicated this in the sample collection section line 141.

7. Describe how antibiotics used in the study were selected

**Response:** Descriptions on how antibiotics used in the study were selected has been added in line 174 to 175**.**

8. Did authors use only CLED for identification of Gram-positive and Gram-negative bacteria? How S. aureus and Micrococcus was differentiated? Overall, the types of culture media used for AST were not indicated.

**Response:** We differentiated Staphylococcus aureus from Micrococcus species by observing colony characteristics and performing a gram stain. The type of culture used for AST has been indicated in lines 175-177

9. As the definition of multi-drug resistance may vary it advisable to include operational definition for multi-drug resistance.

**Response:** We have added in the materials and methods a subsection of the definition of terms. The definition of multi-drug resistance has been included in lines 143-153

10. The sample size for was not indicted in the main method section.

**Response:** The sample has been indicated in methodology line 114

11. Consider including 95% CI for culture confirmed UTI.

**Response:** 95% CI has been added to the culture confirmed UTI in the abstract, results and discussion sections

12. Figure 1, Y-axis is not labelled. Also include frequency and % on the top of each bar

**Response:** Figure one was changed to a table 2 which indicates frequency and percentage

13. It is difficult to understand the results presented in Table 3. Consider including R, I, and S

**Response:** Table 3 has been revised for clarity and changed to table 4 since we added another table

Minor comments

1. Line# 135 “…..Identify…..’ the first letter should not be capital

2. ‘Gram-positive’ and ‘gram-positive’ is interchangeably used. It is good to stick to one. ‘Gram’ or ‘gram’

**Response:** The first letter of "identify" has been written in lowercase. "Gram positive" and "gram positive" have been revised to "gram positive." However, when starting a sentence, "Gram" is written with a capital letter

**Comments from Review 2**

Comments for Reviewer #2 were on the sticky note on the PDF file. We have addressed all comments and modified the manuscripts

In title you said etiological profile, is it include fungi?

**Response:** The title has been revised to reflect the bacterial etiology; line 1

Abstrct background:Where UTI is common infection?. Avoid use of short form in abstract. Better to be consistence as per title or should modify the title accordingly. Foristance febrile underfive,antibiotic vs antimicrobial,proportion of UTI vs etiology profile

**Response:** We rephrased the sentences for clarity and to ensure consistency, and short-form words were omitted; lines 17-23

Abstract methodology: Why only febrile? Is fever the only criteria to be suspected as UTI for under five? Operationalize this. The method is not written well, rewrite again

**Response:** The materials and methods subsection in the abstract has been rewritten for clarity; Lines 25-32

Abstract results : rewrite the result very well. Like of all 28.8% had Lab, confirmed means? do you mean culture confirmed? write the organism name in italic. Is circumcision a common practice for both male and female or for only for male? is those for female taken as nor not applicable? Rewrite bacteria in italic. Is suceptibility pattern for overall? or *E.. coli* & *S. aurues*

**Response:** The result subsection of the abstract has been rewritten for clarity. Bacteria have been written in italics; the susceptibility patterns are for *E.coli* and *S. aureus*; Lines 34-43

**Introduction**

Add One paragraph for common cause of UTI from different literature.

**Response:** A paragraph detailing the common causes of UTI has been included; Line 62-69

what does the renal functions of the kidney mean?

**Response:** The sentence has been revised to reflex that, Early diagnosis is essential to preserve and maintain the proper functioning of the kidneys and prevent any further decline in their function. Line 56-57

uncircumcision in male?

**Response:** Yes uncircumcission in male, The sentence has been paraphrased; Line 43&73

The prevalence of UTI among under-five children ranges from 7.4 % to 43.6% where this studies conducted

**Response:** The reported prevalence was indicated from Nigeria and Tanzania; Line 59-60

The risk factors for acquiring UTI in under-five children are multiple and differ with

geographical locations, settings, and seasons. Add reference

**Response:** Reference number 9 has been added; Line 71

Antimicrobial resistance (AMR) has become significant concern worldwide.add reference

**Response:** Reference number 14 was added; Line 77

Can you generalize with your specific hospital data about the treatment? or can leads you to change treatment guidelines?

**Response:** The sentence has been ommited, since the same information were discussed in line 89-94

**Materials and methods**

Line 91, Can you clarify on primary health care and district hospital? in your title it says primary health care whereas the study is conducted in district hospital? Besides this your study area description don't indicate as whether it is urban or rural but in reasoning or gab analysis your said that rural community lacks attention?

**Response:** Yes, in Tanzania, a district hospital is considered a primary healthcare facility. The Magu district, where our research was conducted, is situated in the rural parts of the region. However, when classifying the demographic data, we categorized urban as individuals from the town area of Magu, and rural as those from other areas outside the town; Line 103

why only children with fever?

**Response:** Fever is a common symptom of various illnesses, the aim of recruiting children with fever ensured that,study participants have a higher likelihood of having the targeted condition; Line 106-109

The total sample size should be indicated here.

**Response:** The total sample size was indicated; Line 114

In Data collection your sociodemographic is limited why?

**Response:** The scope and objectives of this study prioritized these sociodemographic variables; Line 121

Is Sekou Toure Regional Referral Hospital different from above hospital?

**Response:** Yes, the study was conducted at Magu District Hospital. However, the samples were analyzed at Sekou Toure Regional Referral Hospital since the District hospital lacked the capacity to process culture samples

Line 131- 132, write more how do you handle in case of mixed growth

**Response:** Handling of mixed growth of bacteria has been indicated; Line 161-162

Line 135, Identify should start with small letter

**Response:** The word Identify written starting with small letter Line 165

is this the only biochemical test? how do you identify like *P. aerigenosa* & *Acitenobacter* spp

**Response:** Oxidase test used to identify *P. aerigenosa* & *Acitenobacter* spp has been added Line 168-169

Antimicrobial susceptibility testing: Can we report erthromycin for organisms from UTI? this shows you haven't followed the CLSI properly.

**Response:** Erthromycin disk has been removed from this manuscript

Extended-Spectrum Beta-Lactamase Detection: Is this for the three or for one? if for one you have write cefotaxime or cftriaxone or ceftazidime. is the increase of zone of inhibition towards CAZ or CXT or both?

**Response:** The sentence has been rephrased Line 192-193, 199-201

Quality assurance: As indicated in the quideline, on which guidelines?

**Response:** The sentence” as indicated in the quideline was ommited since checking expiration date and assess reagents does not need any guideline.

Data analysis: ≤ 0.2, this cut of value most of the time is 0.25. Do you have some justification for it?

**Response:** The choice of a cut-off value in data analysis can depend on the specific field of study, the nature of the data, and the research question being addressed, This cut-off value allows for broader inclusion of variables that may have some potential association with the outcome of interest. Line 223

Ethical approve: include ethical number

**Response:** Ethical clearance number MUHAS-REC-12-2022-1484 has been added Page 228

**Results:**

Socio-demographic and clinical characteristics: so you you justify as this study is conducted in rural area: No need of repeating 368

**Response: The study was conducted in Magu District, which is primarily a rural area. However, during the recruitment of participants, some individuals residing in the town were included, categorizing them as urban participants. Additionally, participants from villages near Magu town were also included, classifying them as rural area participants.** The sentence has been paraphrased. Line 241.

The proportion of UTI among under-five children: why you classify the age only in two category? do you have justification?

**Response: Dividing age into two categories** was made in order to compare the usage of nappies between two distinct age groups: children under 24 months and those above 24 months. Line 247-248

I think the use of p-value is not important here as the title is the proportion of UTI

**Response:** The p-value is used in proportion tests to assess the statistical significance of the observed sample proportion compared to a hypothesized population proportion. It helps determine whether the difference between the observed proportion and the hypothesized proportion is statistically significant or simply due to random chance Line 248

what does it means 0-2 days? is there a zero day?

**Response:** The sentence has been rewritten as ≤2 days; Line 251

is there a means that you know a child of five days have abdominal pain or not? similarly for others

**Response:** The doctor reported this after obtaining the child's clinical history from the parents or guardian and conducting a thorough physical examination; Line 244

Put p in italic.

**Response:** P value has been written in italic Line 247-260

is UTI only expected only at high temp what about lower temp?

**Response: According to the World Health Organization (WHO), a body temperature of 38 degrees Celsius is generally considered a threshold for fever in both adults and children. Therefore, our studies specifically recruited children under the age of five with a fever of 38 degrees Celsius and above.** Line 258-259

The line with overall is not correct. put it at the end of the table and the table header should have a line on the top and bottom. why only positive? remove p-value from here? is the p-value from chi-square of logistic regression?. 0.2 should be written 0-2. Axillary temperature of ≥39 should be written well. Place of residence, why it comes last? why not on socio-demographic? font style and size should be similar

**Response:** The line with overall proportion has been removed ,The line was added on top and bottom of table header, Negative n (%) column has been added in the table. P value used is from chi square and it tells as if the difference in proportion is significant or not significant. Duration of fever 0.2 was written well as <2; An axillary temperature of ≥39 was written well; The place of residence variable was shifted to demographic factors; Font size was setted Table 1

Modify the title “Bacteria causing UTI among under-five children”. you can *staphylococcus aureus* write in short form once you write in long and short form then after.

**Response:** The title has been modified to” Bacterial uropathogens isolated from Urinary Tract Infection positive under five children. *Staphylococcus aureus* has been written in short form.

Figure : think over febrile children. modify the organism name on the figure. remove the key from main figure and indicate each organism separately. Give title for x-and y-axis. Indicate number and % on bar graph.

**Response:** The figure was presented in Table including frequency and per cent of all isolates, Table 2

Factors associated with UTI: This sections seems more of discussion. try to write it properly again. what is CPR, and APR? is this common? I know COR & AOR? where candidate or associated? include OR,95%CI & P-value for each cells which is candidate.

**Response:** CPR =Crude Prevalence Ratio, APR=Adjusted Prevalence Ratio. These are used when the prevalence of the outcome is greater than 15. Our prevalence in this study was 28.8; hence, we used CPR and APR instead of COR&AOR. CPR and APR have been included. Lines 343 - 349

Table 2: Characteristics should be variables, Duration of fever, 0.2 should be 0-2, why uppercase for some variable? check for all. Male circumcision, how do handle for those female here?

**Response:** Characteristics have been changed to variable, Duration of fever 0.2 changed to ≤2, Upper case was changed to lower case in YES and NO. Females were excluded from the data as analysis was done. Table 3

write uniformly for all antibiotics you write in lower case for some and uppercase here **Response:** Antibiotic was written in lowercase; Line 312

Tabel 3: consistence issue. when we think susceptibility we have to think about Sensitive, Intermediate and Resistance both your table doesn't indicate this one. S.a not scientific similarly for the others. Key, this should be out of table and as keynote:

**Response:** The table title has been revised as sesntivity pattern for consistency. The pathogens names has been written in scientific form and the Key shifted out of the table; Table 4

MDR, ESBL and MRSA Should be defined in your method

**Response:** Defination of MDR, ESBL and MRSA has been added in the methods new susbsection of definition of terms line 143-153

**Table 4,** your title lucks consistency. I think MRSA and the like is should not be presented in one table. present with other way like pie chart. MDR, This should be presented separately & should be shown how it comes.

**Response:** MRSA and ESBL producers bacteria have been presented in a histogram in Figure 1 and MDR has been presented separately in Table 5

**Discussion**

I think it better you start with the strength and weakness of the study. rather than summarization of your finding. Your discussion poorly organized doesn't discus about the most important part of the study, ESBL, MRSA & MDR. Abserve consistency

**Response:** Discussion has been revised

Better show the number for comparison and put your study in CI. Similarly for the others include. Is this the only reason? what about nitrofurantoin?

**Response:** The number for comparisons has been added, and CI has been added in Line 343 to 349. Nitrofurantoin has been included in the discussion, Line 371 to 373.

“High resistance rates were observed to antibiotics commonly used in rural settings”, what does it mean? Provide reference

**Response:** The sentence has been rewritten as ‘The antibiotics frequently prescribed for empirical treatment Line 381

Do you have scientific background or reference for this? “ as this study was conducted in rural areas where UTI is more likely to be community-acquired, whereas, in urban areas, UTI is more likely to be acquired at the hospital from possibly admitted patients.

**Response:** The sentence has been removed as there is no proper reference to support its validity

put reference at the end of the sentence?

**Response:** The reference has been moved to the end of the sentence Line 384 and 400

Indicate which age group? Wearing of nappies, you assessed for all age?

**Response:** Wearing nappies werer assessed in children under 24 months; Line 402

you have to discuss circumcision separately and give emphasis even if your study subjects is both male and females. compare and contrast with other finding.

**Response:** Circumcision has been discussed in separate paragraph; Line 404-410

what does it mean? is this limitation of your study?if yes then write in the limitation section

**Response:** Limitation section has been added; Line 419-422

Conclusion and recommendation: Write as conclusion or separately. following the journal guideline. Not clear it is miss-leading; what does it mean? why only on AMR? looking at your title

**Response:** The section conclusion and recommmednation has been written as conclusion and content revised for clarity; Lines 425-432

Acknowledgements: we should start with capital letter. Who do you refer in “gratitude to respondents who willingly participated”?

We’ has been wrritten starting with capital letter and the sentence has been rephrased for clarity; Lines 438-439

Reference: follow the journal guideline for referencing

References has been reviewed as per journal guideline; Line 460-577
