# Supplementary material for: Bacterial aetiology, antimicrobial susceptibility patterns, and factors associated with urinary tract infection among under-five children at primary health facility, North-Western Tanzania
Source: PLoS One. 2024 May 6;19(5):e0303369. doi: 10.1371/journal.pone.0303369 (PMC11073726; doi:10.1371/journal.pone.0303369)
Supplement: Attachment — Submitted filename: Response to Reviewers Comments.docx [file pone.0303369.s005.docx]

**POINT TO POINT RESPONSE TO EDITOR AND REVIEWERS’ COMMENTS**

**Editor’s comments**

Please review your reference list to ensure that it is complete and correct. If you have cited papers that have been retracted, please include the rationale for doing so in the manuscript text, or remove these references and replace them with relevant current references. Any changes to the reference list should be mentioned in the rebuttal letter that accompanies your revised manuscript. If you need to cite a retracted article, indicate the article’s retracted status in the References list and also include a citation and full reference for the retraction notice.

**Response:** All references have been reviewed and errors corrected

**Comments from Reviewer #1**

Thank you for addressing most of my comments. Comment 8 was not fully addressed I don’t think morphology and gram staining is sufficient to differentiate *Staphylococcus aureus* from *Micrococcus* you may indicate this in limitations.

Response: Thank you for the comment. We have rephrased the paragraph related to previous comment 8 to indicate that We differentiated *S. aureus* from other *Staphylococcus* and *Micrococcus* species by coagulase tests. Line 164-165

**Comments from Review # 2**

Comments from Reviewer #2 were on the sticky note on the PDF file. We have extracted the comments from the PDF file and addressed all of them as follows:

1. **Materials and methods in the abstract:**
i. Method only; remove material
ii. Bring “*District Hospital in Mwanza Region, North-Western Tanzania, between March and April 2023* to the objective to make it SMART and remove from the material and methods*.*
iii. Still, the statistical analysis is not clearly written. OR, and CI, cut of a p-value.

**Response:** We have addressed the comments related to the materials and methods in the abstract section as suggested. Line 23-30

2. **Results in the abstract:**
i. Make the urinary tract infections culture-confirmed
ii. Multidrug resistance was observed in 33.0% of isolates, Methicillin Staphylococcus aureus in 23.1% of Staphylococcus aureus, and extended-spectrum beta-lactamases in 25% of gram-negative bacteria. Make this in separate sentences as they are different, and the sentence does not make sense.

**Response:** we have revised to indicate culture-confirmed urinary tract infection and separated the sentences as indicated for clarity. line 33-40

iii. Urinary tract infection was associated with vomiting, age, dysuria, abdominal pain, nappy use, and uncircumcision in males. Should be indicated which category associated with UTI, age, for instance, which range?, vomiting presence or absence, abdominal pain presence or absence similarly in conclusion.

**Response:** We have revised the sentence in the abstract result section to clearly indicate the categories associated with UTI. Line 39-40

3. **Conclusion in abstract**

what are commonly prescibed antibioitcs? Is your recomendation only the suspetibitly pattern? What about prevention and control based on your findings from associated factors?

**Response:** The conclusion has been revised to include the list of common antibiotics and recommendations regarding associated factors. Line 41-45

4. **Keywords:**

Add some words like ethiology agent, associated factros.

**Response:** We have added the words aetiologic agent and associated factors to the list of keywords. Line 49

5. Use a square bracket for all references.

**Response:** We have used square brackets for all references

6. Studies conducted in Nigeria and Tanzania have reported the prevalence of UTI in under-five children ranging from 7.4% to 43.6% (1,4,5). Better use some national data or meta-analysis data from Africa or WHO report. why you focused on Nigeria and Tanzania?

**Response**: We have included reference # 4 with a systematic review and meta-analysis data. Line 57

7. First time, write long and short forms and use short forms throughout the document.

**Response:** The comment has been accepted and addressed in the entire manuscript

8. In the study design, setting, and population, the study involved under-five children with a fever seeking healthcare services. Do you think all UTI patients presented with fever? What is the standard for under-five children to be suspected of UTI? Unless otherwise, this leads to a selection bias.

**Response:** We have revised the paragraph for clarity. Line 101 - 104

9. We recruited children with fever who the attending clinician suspected to have UTI based on signs and symptoms such as fever of 38 degrees Celsius or above, pain or burning when urinating, frequent urination, discoloured urine, lower abdominal pain, or vomiting. this contradict with the above sentence.

**Response:** As per the response to comment # 8 above, we have revised the paragraph for clarity and to avoid contraction. Line 101-104

10. The study did not include children who used catheters within 72 hours because catheter insertion may directly inoculate microorganisms into the urinary bladder. reference is needed which stanard recomend this?

**Response:** We have added reference # 23 which indicates the effect of catheterization on UTI. Line 106

11. Risk factors: do you think nappy use works for all?

**Response:** Nappy use as a risk factor for UTI may not work for all but has been indicated as a factor in other study

12. Definition of terms: Make in bullect with bold for the title. also include for UTI.

**Response:** We have written the sentence in the definition of terms as suggested and included the definition of UTI. Line 137-147

13. Do you collect all the specimens with midstream? What about for catheters?

**Response:** For the purpose of this study, we instructed parents and guardians to collect midstream urine. We did not use a catheter for urine collection.

14. In Table 1: *Frequency and percentage of participants calculated as number within the variable category divided by the total sample size of the variable, ** proportion of patients within the variable category with no Urinary Tract Infection calculated as number of divided by the total number of individuals in the variable category. *** proportion of patients within the variable category with Urinary Tract Infection calculated as number of cases divided by the total number of individuals in the variable category. I don't think this is important, remove from table to *.

**Response:** The table legend has been removed as suggested. Line 260

15. In Table 4, Morganella spp. You said M. morgannii in the above makes it uniform.

**Response:** We have changed to M. morganii for uniformity. Line 312

16. In table 4: Give the title keynote: NIT= Nutrofurantion; it is better to use NIT-Nutrofurantion.

**Response:** The Table 4 legend has been corrected as suggested. Line 313-315

17. Discussion: use the figure and country; double referencing is not important. similarly correct for others. all prevalence figures from another study should be included in all the discussion. Unless otherwise it is difficult to now whether higher, lower or inline.

**Response:** The discussion section has been re-worked to address the comment accordingly
